# Management Strategies for Cutaneous Adverse Events Caused by Kinase Inhibitors in Oncology: A Systematic Review and Meta-Analysis

**DOI:** 10.3390/cancers18203263

**Published:** 2026-10-10

**Authors:** Renáta Árok, Fanni Adél Meznerics, Andrea Lancz, Anna Sára Lengyel, Anna Walter, Péter Hegyi, András Bánvölgyi, Norbert Kiss

**Affiliations:** 1 Department of Pharmacy, Budapest Bajcsy-Zsilinszky Hospital and Clinic, 1106 Budapest, Hungary; 2 Centre for Translational Medicine, Semmelweis University, 1085 Budapest, Hungary; 3 Department of Dermatology, Venereology and Dermatooncology, Faculty of Medicine, Semmelweis University, 1085 Budapest, Hungary; 4 Department of Internal Medicine, Markusovszky University Teaching Hospital, 9700 Szombathely, Hungary; 5 HCEMM-SU Translational Dermatology Research Group, Semmelweis University, 1094 Budapest, Hungary; 6 Institute for Translational Medicine, Medical School, University of Pécs, 7624 Pécs, Hungary; 7 Institute of Pancreatic Diseases, Semmelweis University, 1083 Budapest, Hungary

**Keywords:** kinase inhibitors, cutaneous adverse events, systematic review, meta-analysis, cancer therapy

## Abstract

Background: The use of targeted therapies in oncology has increased significantly, but associated cutaneous adverse events often impair quality of life and adherence. Despite numerous available interventions, there is no unified protocol, and many patients discontinue therapy due to adverse events. This systematic review and meta-analysis aimed to evaluate preventive and reactive interventions for cutaneous adverse events in adult cancer patients receiving kinase inhibitors. Methods: A search was performed in MEDLINE (via PubMed), EMBASE, and CENTRAL (The Cochrane Central Register of Controlled Trials), using predefined search terms. Outcomes were incidence, severity of cutaneous adverse events (grades 1–4 and 3–4), quality of life and adherence to oncologic therapy. Results: After screening 35,072 records, 9 studies (1853 patients, per protocol) met the inclusion criteria for the meta-analysis, while 52 were included in the systematic review. Preventive topical agents significantly reduced the incidence of grade 1–4 cutaneous adverse events (OR, 0.55; 95% CI, 0.38–0.80; *p* = 0.012; I^2^ = 16%), whereas systemic interventions showed no significant benefit in this category. Conversely, for grade 3–4 events, systemic interventions were associated with a significantly reduced incidence (OR, 0.24; 95% CI, 0.14–0.41; *p* = 0.002, I^2^ = 0%), while topical agents had no significant effect. For reactive interventions, no consistent evidence of effectiveness was found, with high heterogeneity and a limited number of studies. Conclusions: In patients receiving kinase inhibitors, systemic antibiotics were associated with a reduction in grade 3–4 cutaneous adverse events, while pooled topical interventions significantly reduced grade 1–4 events. However, the effectiveness of individual topical agents, including urea, remains uncertain.

## Simple Summary

Cutaneous adverse events frequently occur in patients receiving kinase inhibitors and can impair quality of life, reduce treatment adherence, and threaten the continuation of anticancer therapy. Although many preventive and reactive treatments are used, it remains unclear which approaches are most effective. This systematic review and meta-analysis evaluated these strategies in adults receiving kinase inhibitors. Preventive systemic antibiotics were associated with a reduction in severe cutaneous adverse events, while preventive topical treatments as a group reduced events across all severity grades. Differences between the included studies made it difficult to assess individual treatments, and evidence for reactive treatments remained limited. Further well-designed studies using standardized methods are needed to determine which treatments are most effective for specific types of cutaneous adverse events.

## 1. Introduction

Cancer remains a major global health burden, with an estimated 20.7 million new cancer cases and 9.7 million cancer deaths worldwide in 2022 [1]. Over the past two decades, targeted therapies—particularly kinase inhibitors—have become an important component of cancer treatment, now accounting for 31% of FDA-approved oncology drugs. These agents are widely used in the treatment of cancers such as lung, colorectal, head, and neck malignancies and are generally less toxic than conventional cytotoxic agents but can cause specific side effects, such as cutaneous adverse events [2]. This is often related to the inhibition of receptors involved in skin homeostasis, such as EGFR, which is expressed in keratinocytes, sebaceous glands, and the epithelium of hair follicles, and it regulates keratinocyte proliferation and differentiation and supports epidermal barrier maintenance and wound healing. Inhibition of these receptors can disrupt epidermal integrity and trigger mobilization of inflammatory cells, potentially leading to characteristic cutaneous adverse events, although the exact pathophysiology remains unclear [3,4].

Cutaneous adverse events of kinase inhibitors are classified according to the CTCAE (Common Terminology Criteria for Adverse Events) criteria [5] developed by the National Cancer Institute (NCI) of the National Institutes of Health (NIH). This classification utilizes a standardized five-point scale to assess the severity of side effects, providing unique clinical descriptions for each type, allowing consistent assessment and comparison. Although these adverse events can be severe [6,7], they are rarely life-threatening. However, severe adverse events negatively affect quality of life and general well-being [8], reduce patient adherence, and compromise treatment efficacy, with 76% of the patients requiring dose modification or discontinuation of kinase inhibitor therapy when they occur [9,10,11].

Despite the availability of multiple preventive and therapeutic strategies, there is no consensus on the optimal approach to the management of these events [12]. To address this gap, we conducted a systematic review and meta-analysis to identify the most effective and well-tolerated interventions for the prevention and treatment of kinase inhibitor-induced cutaneous adverse events. We hypothesize that the implementation of evidence-based prevention and treatment strategies for the management of cutaneous adverse events would improve patient adherence and optimize clinical outcomes in patients receiving kinase inhibitor-based targeted therapy.

## 2. Materials and Methods

We conducted our systematic review and meta-analysis in accordance with the Cochrane Handbook [13] and report our results based on the recommendations of the PRISMA 2020 guidelines [14,15]. The protocol of the study was registered on PROSPERO (registration number CRD42023485089), and we fully adhered to it.

### 2.1. Eligibility Criteria

Trials were included that enrolled adult oncology patients treated with tyrosine kinase inhibitor-targeted therapy, excluding the pediatric population. Randomized controlled clinical trials, and prospective and retrospective cohort study types were included, and literature reviews, case studies, case series, case reports, background information, expert opinions, non-EBM guidelines, abstracts, comments, letters to the editor, and protocols were excluded. Language was not a limiting factor in the selection.

Articles were also required to address the prevention or management of cutaneous adverse events in adult cancer patients treated with kinase inhibitors targeting various receptors (EGFR, ALK, MET, FGFR, VEGFR, JAK, TRK, RET, BCR-ABL), alone or in combination with other treatments.

### 2.2. Search Strategy and Selection Process

On 21 November 2023, a systematic search was conducted in three databases—MEDLINE (via PubMed), EMBASE, and CENTRAL (The Cochrane Central Register of Controlled Trials)—using a predefined search key, which is available in the Supplementary Material (Figure S1). Articles were screened for duplicates, which were automatically and manually removed using the EndNote 21 (Clarivate Analytics, Philadelphia, PA, USA) citation and reference management tool [16] based on overlapping publication years, authors and titles. Remaining records were manually screened by title and abstract, followed by full-text review using Rayyan (Cambridge, MA, USA) [17]. Selection was performed by two independent reviewers (R.Á. and A.L.) who were blinded to the assessments of the other reviewer. Conflicts were resolved, and final articles were selected by consensus after the two reviewers were unblinded.

### 2.3. Data Extraction

Data from each eligible article were collected independently by two authors (R.Á. and A.L.). The information extracted was first author, year of publication, DOI reference, study type, type of adverse event, study population with the patient characteristics, types of interventions, intervention duration, follow-up duration and types of outcomes. Preventive or therapeutic treatments for established cutaneous adverse events (reactive treatment) were recorded, alone or in combination with other interventions. Outcomes assessed included efficacy (e.g., incidence of cutaneous adverse events, response to therapy, improvement of cutaneous adverse events, number of lesions of skin eruptions observed), change in quality of life, and adherence to the oncologic therapy.

### 2.4. Study Risk of Bias Assessment and Certainty of Evidence

The risk of bias was independently assessed by two authors (R.Á. and A.L.) according to the recommendations of the Cochrane Collaboration, using the Risk of Bias Tool 2 (RoB2) for randomized controlled trials of interventions [3,4,10,18,19,20,21,22,23,24,25,26,27,28,29,30,31,32,33,34,35,36,37,38,39,40], Risk Of Bias In Non-randomized Studies of Interventions (ROBINS-I) [9,41,42,43,44,45,46,47,48,49,50] and Methodological Index for Non-Randomized Studies (MINORS) for single-arm studies of interventions [51,52,53,54,55,56,57,58,59,60,61,62,63,64,65,66,67,68,69,70,71,72,73,74]. Disagreements were resolved by consensus. The quality of included studies was assessed using GRADEpro GDT [75].

### 2.5. Synthesis Methods

Odds ratios (ORs) were used as the effect size measure, along with 95% confidence intervals (CIs). To calculate both study-specific and pooled odds ratios, we extracted or calculated the number of patients in each group and the number of those who experienced the event of interest, based on data available in the included studies. When multiple types of adverse events (AEs) were reported within a study, we selected the type of AE that had the highest number of events in the intervention group. This approach ensured that we captured the most commonly occurring AE, thereby maximizing the amount of event information contributed by each study to the pooled analysis. Where intervention-specific meta-analyses were not feasible because of the limited number of studies, interventions were grouped into broader systemic and topical categories for quantitative synthesis. Pooled ORs based on raw data were calculated by the Mantel–Haenszel method [76,77]. To treat the zero cell count, we used the exact Mantel–Haenszel method (without continuity correction) (as recommended in the literature [78,79]). In the forest plots, for a cell count of 0, the individual study OR with a 95% CI was calculated by adding 0.5 as a continuity correction (it was used only for visualization in the forest plot). To estimate the heterogeneity variance measure (τ2), the Paule–Mandel method [80] (recommended by Veroniki et al. [81]) was used with the Q profile method for confidence intervals. For subgroup analysis, we used a fixed-effects “plural” model (aka. mixed-effects model). To assess the difference between subgroups, we used a “Cochrane Q” test (an omnibus test) between subgroups [82]. The null hypothesis was rejected at a significance level of 5%. All statistical analyses were performed with R (R Core Team 2024, v4.4.2 [83]) using the meta (Schwarzer 2019, v7.0.0 [84]) package for basic meta-analysis calculations and plots.

## 3. Results

### 3.1. Study Selection

**Figure 1:**
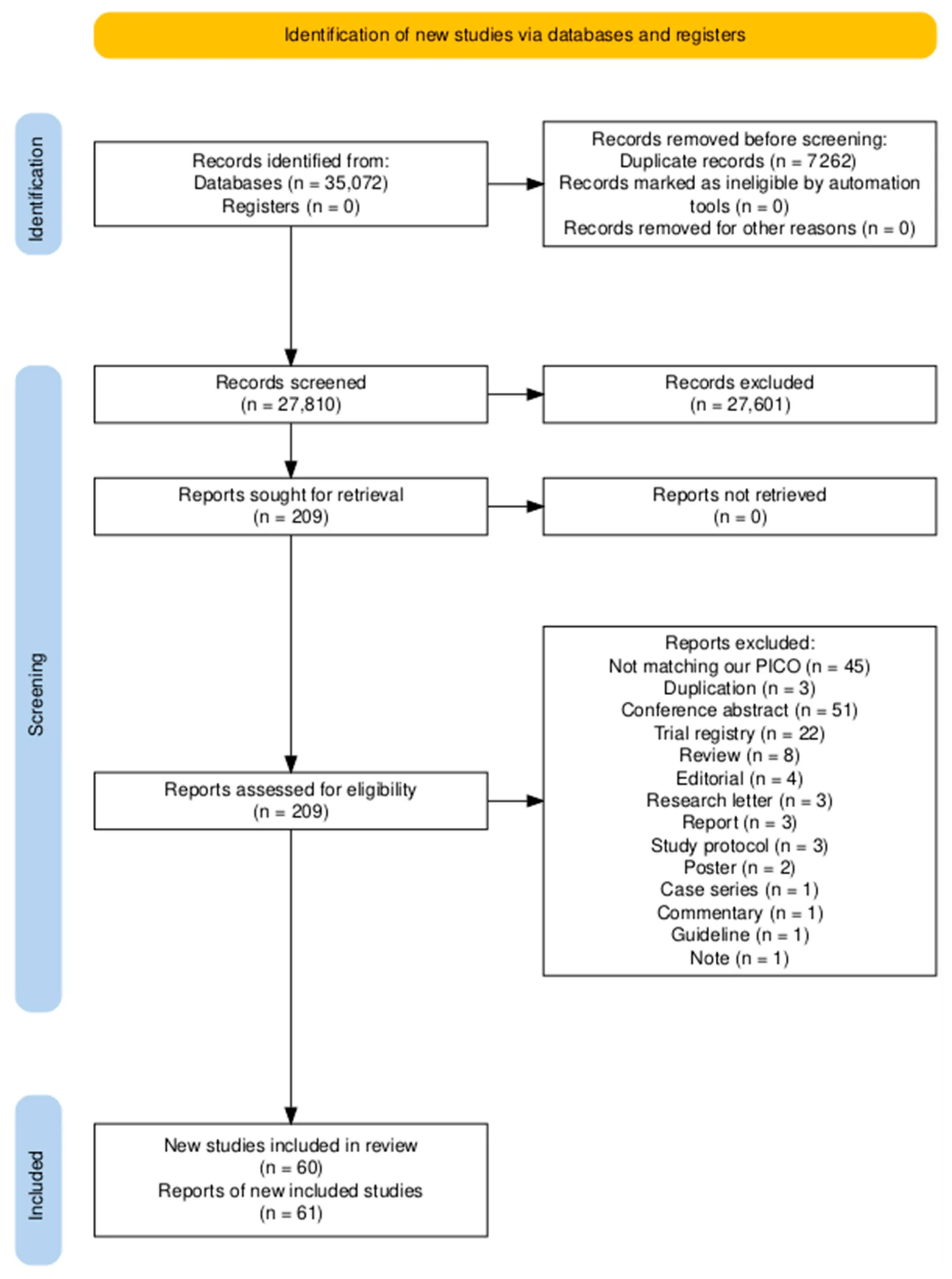
PRISMA 2020 flow diagram of the search and screening process [85].

The search yielded 35,072 records, reduced to 27,810 articles after automatic and manual duplicate removal. The remaining records were screened by title and abstract, leaving 209 articles for full-text review. Of these, 148 were excluded for the reasons detailed in Figure 1, resulting in 61 eligible articles [3,4,9,10,18,19,20,21,22,23,24,25,26,27,28,29,30,31,32,33,34,35,36,37,38,39,40,41,42,43,44,45,46,47,48,49,50,51,52,53,54,55,56,57,58,59,60,61,62,63,64,65,66,67,68,69,70,71,72,73,74].

Nine studies [10,19,21,22,23,27,28,30,35] were included in the meta-analysis; all evaluated preventive interventions and were randomized controlled trials. The remaining 52 articles [3,4,9,18,20,24,25,26,29,31,32,33,34,36,37,38,39,41,42,43,44,45,46,47,48,49,50,51,52,53,54,55,56,57,58,59,60,61,62,63,64,65,66,67,68,69,70,71,72,73,74] could not be evaluated or pooled. They differed substantially in interventions, study designs, follow-up periods, and reported outcomes, and many did not provide comparable incidence or severity data suitable for quantitative synthesis. Therefore, these studies were assessed in the systematic review.

### 3.2. Basic Characteristics of Included Studies

Characteristics of included articles are detailed in Table S1 [3,4,9,10,18,19,20,21,22,23,24,25,26,27,28,29,30,31,32,33,34,35,36,37,38,39,40,41,42,43,44,45,46,47,48,49,50,51,52,53,54,55,56,57,58,59,60,61,62,63,64,65,66,67,68,69,70,71,72,73,74].

### 3.3. Quantitative Analysis

Intervention-specific meta-analyses were not feasible for most treatments because only a limited number of studies evaluated each individual intervention. Analyses according to specific types of cutaneous adverse events were also explored; however, further stratification resulted in small and fragmented subgroups that did not allow meaningful pooled estimates or a sensitivity analysis based on an alternative adverse-event selection approach.

**Figure 2:**
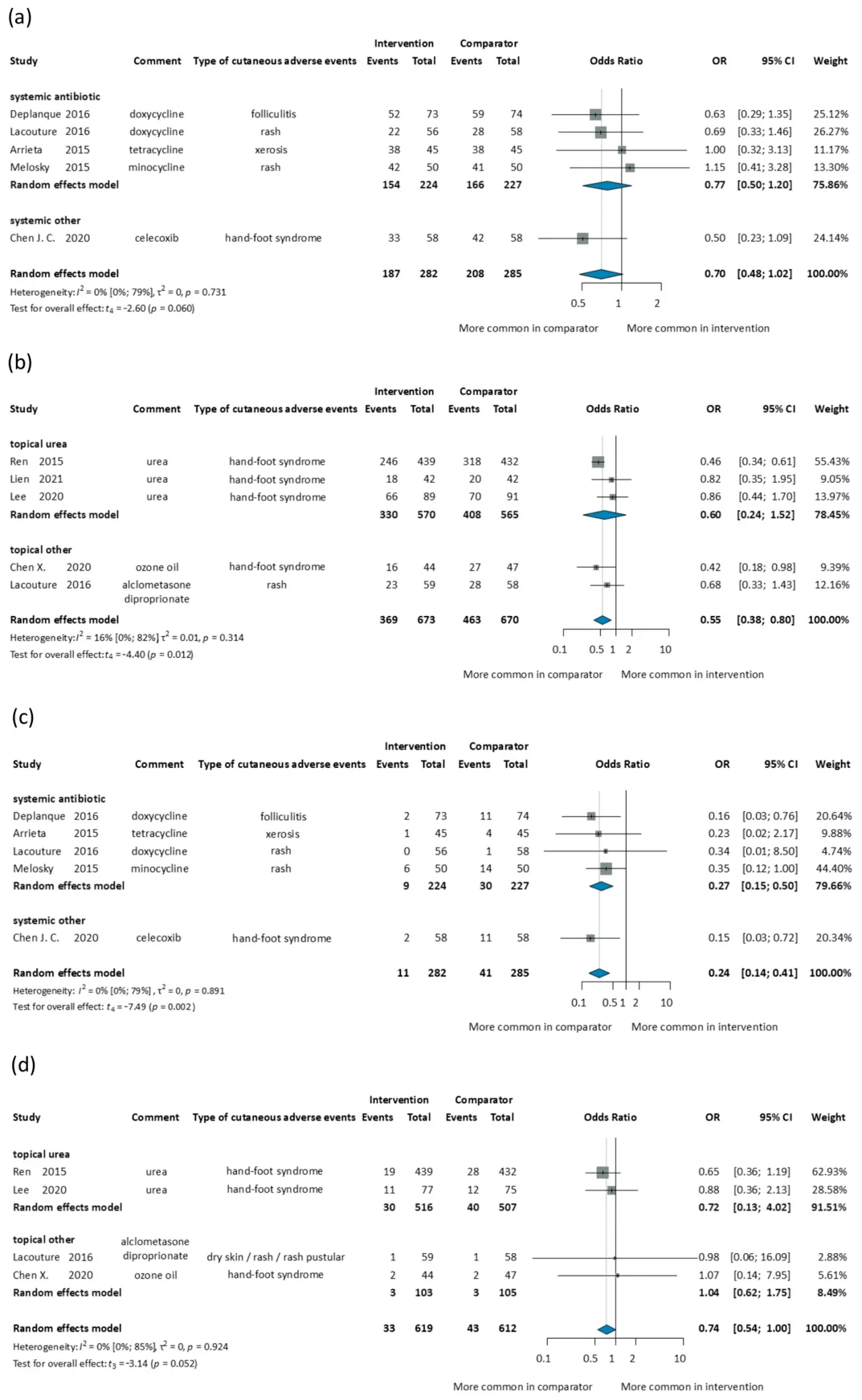
Forest plots showing the incidence of cutaneous adverse events of varying severity (grades 1–4) associated with preventive medications in controlled studies; (**a**) systemic interventions for grade 1–4 adverse events [10,19,21,23,27]; (**b**) topical interventions for grade 1–4 adverse events [22,27,28,30,35]; (**c**) systemic interventions for grade 3–4 adverse events [10,19,21,23,27]; (**d**) topical interventions for grade 3–4 adverse events [22,27,28,35]. Squares represent study-specific effect estimates, with their size reflecting study weight; horizontal lines represent 95% confidence intervals, diamonds represent pooled effect estimates, and the vertical line represents the line of no effect (OR = 1).

The overall analysis indicated that systemic interventions, including tetracyclines and celecoxib, did not significantly reduce the incidence of grade 1–4 cutaneous adverse events (OR, 0.70; 95% CI, 0.48–1.02; *p* = 0.060; I^2^ = 0%). When analyzed separately, tetracyclines also showed a non-significant effect compared to the control group (OR, 0.77; 95% CI, 0.50–1.20) (Figure 2a). In contrast, the overall analysis of topical interventions, including topical urea, ozone oil and steroid (alclometasone dipropionate), demonstrated a statistically significant effect when compared with controls (OR, 0.55; 95% CI, 0.38–0.80; *p* = 0.012; I^2^ = 16%). However, the urea subgroup did not show a significant effect in reducing adverse events (OR, 0.60; 95% CI, 0.24–1.52) (Figure 2b).

In terms of grade 3–4 adverse events, the overall effect of all systemic interventions significantly reduced the incidence of grade 3–4 adverse events (OR, 0.24; 95% CI, 0.14–0.41; *p* = 0.002, I^2^ = 0%). The systemic tetracycline subgroup had a significantly reduced incidence of grade 3–4 adverse events (OR, 0.27; 95% CI, 0.15–0.50) (Figure 2c). In contrast, the overall effect of topical interventions did not significantly reduce grade 3–4 adverse events (OR, 0.74; 95% CI, 0.54–1.00), and the topical urea group could not be analyzed separately due to insufficient data (Figure 2d).

The heterogeneity and limited number of studies precluded a reliable assessment of the effectiveness of reactive treatments.

### 3.4. Qualitative Analysis

A summary of the studies included in the systematic review is presented in Table S2 (Supplementary Materials). Studies demonstrated different levels of evidence and focused on a broad spectrum of preventive and reactive systemic and topical interventions.

### 3.5. Risk of Bias Assessment and Certainty of Evidence

The RoB2 assessment indicated a low risk of bias or some concerns in randomized controlled trials. Non-randomized comparative studies had a critical risk of bias, while single-arm studies showed poor to moderate methodological quality. The results of the risk of bias assessments are shown in Figures S2 and S3 (RoB2 and ROBINS-I) and Table S3 (MINORS) in the Supplementary Materials. The GRADE assessment indicated a very low certainty of evidence for the quantitative outcome of incidence, as detailed in the Summary of Findings table (Table S4).

## 4. Discussion

Despite their efficacy, anti-tumor kinase-inhibitor treatments are often associated with cutaneous adverse events that can significantly reduce adherence to oncologic therapy. The most frequently reported cutaneous adverse events resulting from kinase-inhibitor therapies include acneiform rash (75–90%), dry skin (xerosis) (35–80%), itching (pruritus) (50%), nail bed inflammation (paronychia) (10–62%), hand–foot skin reaction (HFSR) (9–62%), and inflammation of the oral mucosa (mucositis) (5–15%) [8,86,87,88].

In terms of prevention, our results showed that systemic interventions, particularly systemic tetracyclines, were associated with a reduced incidence of grade 3–4 cutaneous adverse events related to kinase-inhibitor-based targeted oncologic therapies. Although data did not permit assessment of effects on specific event types, included studies consistently reported reductions in acneiform rash, folliculitis and xerosis when antibiotics were used prophylactically. Pooled topical interventions were associated with a significant reduction in grade 1–4 cutaneous adverse events; however, these interventions were clinically heterogeneous, and the urea subgroup alone did not reach statistical significance. The effect of systemic interventions on grade 1–4 events did not reach statistical significance, although the result suggested a possible reduction in risk. This may indicate a clearer effect on severe cutaneous adverse events and may be consistent with the moderate efficacy of tetracyclines reported in acne vulgaris [89]. Topical interventions do not appear to be sufficient to prevent the more severe cutaneous adverse events.

The results of our meta-analysis provide additional support for current guideline recommendations, which include systemic antibiotic use to mitigate severe cutaneous adverse events caused by kinase inhibitors [8,86]. While previous guidelines primarily emphasized general skin care as a preventive topical treatment strategy, our findings suggest a potential benefit of topical interventions overall; however, the effectiveness of individual topical agents remains uncertain.

Due to the limited and highly heterogeneous evidence base, a quantitative analysis of reactive treatment strategies was not feasible; therefore, the available treatments were summarized qualitatively. The available evidence supporting reactive management strategies is limited and varies substantially across interventions. The included studies differed substantially with regard to the kinase inhibitors associated with the cutaneous adverse events, the types of toxicity treated (e.g., acneiform rash, hand–foot skin reaction, and paronychia), the severity threshold at which reactive treatment was initiated, and the scales used for outcome assessment. Treatment initiation ranged from mild toxicity to grade ≥2 or grade 3 events. The interventions themselves were also diverse, including antibiotics, corticosteroids, NSAIDs, retinoids, polyphenols, vitamins and their derivatives, cardiovascular agents, traditional Chinese medicines, and non-pharmacological approaches such as laser therapy or surgical interventions. Treatments were administered orally, topically, or using a combination of both routes. Furthermore, some studies evaluated monotherapy, whereas others used combination treatment. Many studies lacked a control group, while in others the treatment provided to the control group was not clearly defined, such as “best supportive care.” Reported outcomes included complete and partial response, changes in adverse-event severity, time to improvement, changes in quality of life assessed using different instruments, treatment compliance, modification of the kinase inhibitor dose, and pain. To facilitate interpretation, these diverse outcomes were categorized into three broad domains: efficacy, quality of life, and compliance. Collectively, these differences substantially limited direct comparisons between interventions, and pooling the studies would therefore not have provided a clinically meaningful summary effect. Nevertheless, these studies provide clinically relevant information on the management of established cutaneous toxicities and were therefore retained in the qualitative synthesis.

Within the qualitative synthesis, several recurring management approaches were identified across specific types of cutaneous adverse events, including both preventive and reactive strategies. For acneiform rash [8,86,87,88], oral tetracyclines are commonly recommended together with topical antibiotics, corticosteroids, and general skin care. Alternative options such as traditional Chinese medicine [29] and Japanese honeysuckle extract [31] may be beneficial. As pruritus often co-occurs, effective rash control may relieve itching [8]; additional therapies include antihistamines, antiepileptics, doxepin, aprepitant, and topical menthol (1–3%) [8,86]. Paronychia prevention involves gentle hygiene and the use of topical antiseptics, antibiotics, or corticosteroids [8,86,87]; in severe cases, systemic antibiotics or surgery may be required. Topical betaxolol showed promise in two studies, although evidence remains inconclusive [47,74]. Hand–foot skin reactions [8,87,88] may respond to non-steroidal anti-inflammatory drugs and topical agents such as corticosteroids, keratolytics, hydrocolloid patches with ceramide [36], and heparin-based ointments [64]. Among systemic options, 300 mg/day of vitamin E may help [54], while perindopril was ineffective [65]. Xerosis may be managed with non-irritant moisturizers such as urea, colloidal oatmeal, or petroleum-based creams [86].

### 4.1. Strengths and Limitations

Our study has several strengths and limitations. It is the first comprehensive meta-analysis to evaluate the efficacy of preventive strategies for kinase inhibitor-induced cutaneous adverse events. We incorporated randomized controlled trials as well as prospective and retrospective observational studies, encompassing a large and diverse patient population. However, the included studies used heterogeneous interventions and methodologies. Only nine studies were eligible for quantitative analysis, most with small sample sizes. Moreover, the certainty of evidence for the main quantitative outcome was very low according to the GRADE assessment, which further limits the strength of clinical recommendations. Non-randomized comparative studies had a critical risk of bias, while single-arm studies showed poor to moderate methodological quality. In addition, underreporting of adverse events in the original trials may have led to incomplete outcome capture. Another limitation is that selecting a single adverse event when multiple outcomes were reported may have introduced outcome-selection bias.

### 4.2. Implications for Practice

Timely translation of research into clinical practice is essential to maintain up-to-date treatment guidelines [90,91]. Our findings suggest a potential preventive benefit of systemic antibiotics for severe cutaneous adverse events and of topical interventions for grade 1–4 events, although the effectiveness of individual topical agents remains uncertain. Current guidelines primarily provide recommendations for the management of specific cutaneous adverse events [8,86,87,88], whereas our meta-analysis provides pooled estimates of preventive effects across different kinase inhibitors and adverse events. Our findings therefore add quantitative evidence to existing guideline recommendations, although their interpretation should remain cautious because the certainty of evidence in our meta-analysis was very low. Effective prevention may improve quality of life and reduce the risk of discontinuing oncologic therapy.

### 4.3. Implications for Research

Further randomized controlled trials with standardized methodologies are needed to accurately characterize the spectrum of cutaneous adverse events associated with targeted therapies and to develop evidence-based preventive and therapeutic strategies tailored to each reaction. Future studies should employ standardized study designs focusing on a specific type of cutaneous adverse event induced by a given anticancer agent, with clearly defined interventions, routes of administration, follow-up periods, and study arms, including an appropriate control group. In particular, in studies evaluating reactive treatment strategies, baseline clinical severity should be standardized using a uniform grading scale, and a consistent primary endpoint should be used, such as improvement by at least one grade within a predefined time period. Other outcomes may be assessed as secondary endpoints, including time to improvement, quality-of-life scores, adherence to oncological therapy, and the need for dose modification. Greater standardization would facilitate comparisons between studies and enable future quantitative synthesis. Furthermore, identifying patient subgroups at highest risk during kinase inhibitor therapy will be essential to enable more personalized and effective supportive care.

## 5. Conclusions

In patients receiving kinase inhibitors, systemic antibiotics were associated with a reduction in grade 3–4 cutaneous adverse events. Pooled topical interventions reduced grade 1–4 cutaneous adverse events; however, the effectiveness of individual topical agents, including urea, remains uncertain.

## Data Availability

No new data were created in this study. The data used in this systematic review and meta-analysis are available in the full-text articles included in the review.

## Supplementary Materials

The following supporting information can be downloaded at: https://www.mdpi.com/article/10.3390/cancers18203263/s1, Table S1: Characteristics of included studies; Table S2: Summary of the results of studies included only in the systematic review; Table S3: Risk of bias assessment with MINORS tool of non-randomized single-arm studies; Table S4: GRADE assessment for controlled studies; Figure S1: Search key; Figure S2: Risk of bias assessment with RoB2 tool of randomized controlled trials of interventions for outcomes of (A) efficacy, (B) quality of life, and (C) adherence to oncological therapy; Figure S3: Risk of bias assessment with ROBINS-I tool of non-randomized more-arm studies.

## Abbreviations

EGFR	Epidermal Growth Factor Receptor.
ALK	Anaplastic Lymphoma Kinase.
MET	Hepatocyte Growth Factor Receptor.
FGFR	Fibroblast Growth Factor Receptor.
VEGFR	Vascular Endothelial Growth Factor Receptor.
JAK	Janus Kinase.
TRK	Tropomyosin Receptor Kinase.
RET	REarranged during Transfection proto-oncogene.
BCR-ABL	Fusion oncoprotein in chronic myeloid leukemia (CML).
