# Supplementary material for: Management Strategies for Cutaneous Adverse Events Caused by Kinase Inhibitors in Oncology: A Systematic Review and Meta-Analysis
Source: Cancers (Basel). 2026 Oct 10;18(20):3263. doi: 10.3390/cancers18203263 (PMC13702333; doi:10.3390/cancers18203263)
Supplement: Supplementary file 1 [file cancers-18-03263-s001.zip › cancers-4543067-supplementary.pdf]

Supplementary Materials

R. Árok, F. A. Meznerics, A. Lancz, A. S. Lengyel, A. Walter, P. Hegyi, A. Bánvölgyi, N. Kiss: Management strategies for cutaneous adverse events caused by kinase inhibitors in oncology: A Systematic Review and Meta-Analysis.

Table S1. Characteristics of included studies[1-61] 3
Table S2. Summary of the results of studies included only in the systematic review [2,4,5,10-30,32,33,35-41,43-61] 91
Table S3. Risk of bias assessment with MINORS tool of non-randomized single-arm studies 138
Table S4. GRADE assessment for controlled studies 140

Figure S1. Search key 2
Figure S2. Risk of bias assessment with RoB2 tool of randomized controlled trials of interventions for outcomes of (A) efficacy, (B) quality of life, (C) adherence to oncological therapy 136
Figure S3. Risk of bias assessment with ROBINS-I tool of non-randomized more-arm studies 137

((kinas* AND inhibitor*) OR imatinib OR gefitinib OR erlotinib OR sorafenib OR sunitinib OR lapatinib OR pazopanib OR icotinib OR crizotinib OR vandetanib OR cabozantinib OR regorafenib OR trametinib OR afatinib OR apatinib OR alectinib OR ceritinib OR cobimetinib OR lenvatinib OR osimertinib OR neratinib OR brigatinib OR pyrotinib OR binimetinib OR anlotinib OR fruquintinib OR lorlatinib OR larotrectinib OR dacomitinib OR erdafitinib OR entrectinib OR tepotinib OR capmatinib OR pemigatinib OR ripretinib OR avapritinib OR tucatinib OR selumetinib OR pralsetinib OR selpercatinib OR almonertinib OR rociletinib OR olmutinib OR temsirolimus OR everolimus OR dovitinib OR acalatrutinib OR axitinib OR dasatinib OR ibrutinib OR nilotinib OR ponatinib OR tofacitinib OR derazantinib OR futibatinib OR zeteletinib OR mivavotinib OR asciminib OR aumolertinib OR bafetinib OR binimetinib OR bosutinib OR catequentinib OR ensartinib OR entospletinib OR fedratinib OR fruquintinib OR gilteritinib OR infigratinib OR itacitinib OR lazertinib OR lestaurtinib OR linsitinib OR lorlatinib OR mobocertinib OR momelotinib OR defactinib OR nebtabrutinib OR olverembatinib OR pacritinib OR pexidartinib OR pirtobrutinib OR quizartinib OR remibrutinib OR repotrectinib OR ruxolitinib OR savolitinib OR sitravatinib OR sotuletinib OR sunvozertinib OR surufatinib OR tivantinib OR vimseltinib OR vodobatinib OR zanubrutinib)

AND

((adverse* OR side) AND (event* OR effect* OR react*) OR (toxic*))

AND

(skin OR derm* OR cutan* OR mucocutan*)

Figure S1. Search key

Table S1. Characteristics of included studies[1-61]

Study	Year	Patient number of intervention group [ITT (PP)]	Patient number of control group [ITT (PP)]	Age of intervention group [year, median (range)]	Age of control group [year, median (range)]	Type of study	Targeted therapy	Type of malignancy	Type of cutaneous adverse events	Cutaneous adverse events criteria	Type of intervention (preventive or reactive)	Way of administration	Intervention	Control	Dosage	Endpoint	Intervention duration	Follow-up duration/assessment
**Characteristics of included studies in the meta-analysis**																		
Arrieta et al. [1]	2015	53 (45)	54 (45)	56.6 ± 14[*§]	57.5 ± 14[*§]	randomized, open-label trial	afatinib	non-small cell lung cancer	acneiform rash, xerosis, mucositis, pruritus, paronychia, skin	NCI-CTCAE 4.0	preventive	oral	tetracycline	no treatment (general dermatological recommendations)	intervention: tetracycline 250 mg/12 hours; control: basic dermatological advice (e.g. sunscreen, moisturiser, steroids)	efficacy	4 weeks	baseline, 2 weeks, 4 weeks

**Study**	**Year**	**Patient number of intervention group [ITT (PP)]**	**Patient number of control group [ITT (PP)]**	**Age of intervention group [year, median (range)]**	**Age of control group [year, median (range)]**	**Type of study**	**Targeted therapy**	**Type of malignancy**	**Type of cutaneous adverse events**	**Cutaneous adverse events criteria**	**Type of intervention (preventive or reactive)**	**Way of administration**	**Intervention**	**Control**	**Dosage**	**Endpoint**	**Intervention duration**	**Follow-up duration/assessment**
									fissure, folliculitis									
Chen J.C. et al.[2]	2020	58 (58)	58 (58)	51.3 ± 11.7[†§]	57.4 ± 11.1[†§]	randomized, prospective controlled clinical trial	sorafenib	hepatocellular carcinoma	hand-foot skin reaction, rash, alopecia	NCI-CTCAE 3.0	preventive	oral	celecoxib	no treatment	celecoxib: 200 mg twice daily (starting at the same time as sorafenib)	efficacy	12 weeks	various (monthly evaluation)

Study	Year	Patient number of intervention group [ITT (PP)]	Patient number of control group [ITT (PP)]	Age of intervention group [year, median (range)]	Age of control group [year, median (range)]	Type of study	Targeted therapy	Type of malignancy	Type of cutaneous adverse events	Cutaneous adverse events criteria	Type of intervention (preventive or reactive)	Way of administration	Intervention	Control	Dosage	Endpoint	Intervention duration	Follow-up duration/assessment
Chen X. et al. [3]	2020	49 (44)	50 (47)	53,2 ± 11.7[*§]	52,4 ± 11.6[*§]	randomized controlled study	sorafenib	hepatocellular carcinoma	hand-foot skin reaction, rash	NCI-CTCAE 3.0	preventive	oral	ozone oil	urea ointment	intervention: ozone oil 1 mL 3 times a day on affected area; control: 10% urea ointment	efficacy	12 weeks	14 weeks (biweekly evaluation)
Deplanque et al. [4]	2016	73 (73)	74 (74)	64 ± 11[§]	64 ± 11[§]	randomized, prospective, phase II trial (CY	erlotinib	non-small cell lung cancer	folliculitis, skin lesions, xerosis, paro	NCI-CTCAE 3.0	preventive	oral	doxycycline	placebo	doxycycline: 100 mg/day	efficacy	16 weeks	various (treatment period + 1-month post-treatment period; max.

Study	Year	Patient number of intervention group [ITT (PP)]	Patient number of control group [ITT (PP)]	Age of intervention group [year, median (range)]	Age of control group [year, median (range)]	Type of study	Targeted therapy	Type of malignancy	Type of cutaneous adverse events	Cutaneous adverse events criteria	Type of intervention (preventive or reactive)	Way of administration	Intervention	Control	Dosage	Endpoint	Intervention duration	Follow-up duration/assessment
						TAR)			nychia									13.5 months)
Lacouture et al. [5]	2016	56 (56)	58 (58)	65.5 (36–88)*	67.0 (41–85)*	triple cohort, randomized, phase II clinical study (ARCHER	dacomitinib	non-small cell lung cancer	rash, dry skin, dermatitis acneiform, paronychia, rash maculopapul	NCI-CTCAE 4.0	preventive	oral and topical	doxycycline	placebo	doxycycline: 100 mg twice a day	efficacy	4 weeks	8 + 4 weeks

Study	Year	Patient number of intervention group [ITT (PP)]	Patient number of control group [ITT (PP)]	Age of intervention group [year, median (range)]	Age of control group [year, median (range)]	Type of study	Targeted therapy	Type of malignancy	Type of cutaneous adverse events	Cutaneous adverse events criteria	Type of intervention (preventive or reactive)	Way of administration	Intervention	Control	Dosage	Endpoint	Intervention duration	Follow-up duration/assessment
						1042)			ar, rash pustular, fissure									
Lee et al. [6]	2020	148 (130)	141 (117)	60 (53–71)†	62 (55–71)†	randomized, controlled study	sorafenib	hepatocellular carcinoma	hand-foot skin reaction	not mentioned	preventive	topical	urea-based cream	placebo	urea: 20% cream, twice daily on affected area	efficacy	12 weeks	12 weeks
Lien et	2021	arm A: 43 (41);	43 (42)	70.26 ± 7.98†§	70.06 ±	randomized, expe	sorafenib	hepatocellular carci	hand-foot skin	NCI-CTCA	preventive	topical	arm A: hydration cream;	best supportive	urea: 10% cream, twice a day on affected area	efficacy	8 weeks	8 weeks

Study	Year	Patient number of intervention group [ITT (PP)]	Patient number of control group [ITT (PP)]	Age of intervention group [year, median (range)]	Age of control group [year, median (range)]	Type of study	Targeted therapy	Type of malignancy	Type of cutaneous adverse events	Cutaneous adverse events criteria	Type of intervention (preventive or reactive)	Way of administration	Intervention	Control	Dosage	Endpoint	Intervention duration	Follow-up duration/assessment
al. [7]		arm B: 43 (42)			9.95†§	rimental study		noma	reaction	E v4.0			arm B: urea-based cream	care and moisturizing cream				
Melosky et al. [8]	2016	50 (50)	50 (50)	64.9 (for all population)	randomized, three-arm phase	erlotinib	hon-small cell lung cancer	papulopustular/acneiform rash	adapted grading system developed by Perez-	preventive	oral	minocycline	reactive treatment or no treatment	minocycline: 100 mg twice a day	efficacy	4 weeks	12 weeks	

Study	Year	Patient number of intervention group [ITT (PP)]	Patient number of control group [ITT (PP)]	Age of intervention group [year, median (range)]	Age of control group [year, median (range)]	Type of study	Targeted therapy	Type of malignancy	Type of cutaneous adverse events	Cutaneous adverse events criteria	Type of intervention (preventive or reactive)	Way of administration	Intervention	Control	Dosage	Endpoint	Intervention duration	Follow-up duration/assessment
					III trial				Soler et al., based on NCI-CTCAE v3.0									
Ren et al. [9]	2015	439 (432)	432 (432)	51.8 (20.4–86.4)	52.0 (20.2–84.3)	randomized, controlled, open-	sorafenib	hepatocellular carcinoma	hand-foot skin reaction, rash, dry skin,	NCI-CTCAE v3.0	preventive	topical	urea-based cream	best supportive care	urea: 10% cream, third times a day on the affected area	efficacy	12 weeks	14 weeks

Study	Year	Patient number of intervention group [ITT (PP)]	Patient number of control group [ITT (PP)]	Age of intervention group [year, median (range)]	Age of control group [year, median (range)]	Type of study	Targeted therapy	Type of malignancy	Type of cutaneous adverse events	Cutaneous adverse events criteria	Type of intervention (preventive or reactive)	Way of administration	Intervention	Control	Dosage	Endpoint	Intervention duration	Follow-up duration/assessment
						label trial			erythema									
**Characteristics of included studies in the systematic review**																		
Alexandrescu et al. [10]	2007	11 (10)	NA	59.5 (37-83)*	NA	open-label study	erlotinib, sorafenib, cetuximab, panitumumab	colon, renal cell, pancreatic carcinoma	acneiform or papular eruption	NCI-CTCAE v3.0	reactive (grade ≥2)	topical	colloidal oatmeal lotion	no control	applied topically to affected areas three times a day for at least 7 days	efficacy	1 week (at least)	not detailed
Amitay-Lai	2020	Arm 1: 31 (21, day	Arm 3: 30	Arm 1: 66 (40-90)	Arm 3: 71	prospective, rand	erlotinib, gefitinib,	head/neck squa	papulopustula	NCI-CTCA	preventive	topical	chloramphenicol 3% + prednisol	placebo (aqua	chloramphenicol 3%, prednisolone 0.5%	efficacy	4 weeks (30	at week 2 (14 days) and

Study	Year	Patient number of intervention group [ITT (PP)]	Patient number of control group [ITT (PP)]	Age of intervention group [year, median (range)]	Age of control group [year, median (range)]	Type of study	Targeted therapy	Type of malignancy	Type of cutaneous adverse events	Cutaneous adverse events criteria	Type of intervention (preventive or reactive)	Way of administration	Intervention	Control	Dosage	Endpoint	Intervention duration	Follow-up duration/assessment
sh et al. [11]		14; 17, day 30) Arm 2: 30 (23, day 14; 19, day 30)	(25, day 14; 21, day 30)	(day 14); Arm 2: 62 (42-92) (day 14)	(41-87) (day 14)	omized, double-blind, placebo-controlled trial (NCT01256437)	cetuximab, panitumumab	mous cell, colon, lung carcinoma	r rash	E v4.03, NOL			one 0.5% (Arm 1), chloramphenicol 3% (Arm 2) (and sunscreen)	cream) (Arm 3)	topically once a day for 3 weeks and every other day thereafter to complete a total overall treatment of 30 days (and sunscreen in outdoors)		days)	week 4 (30 days)

Study	Year	Patient number of intervention group [ITT (PP)]	Patient number of control group [ITT (PP)]	Age of intervention group [year, median (range)]	Age of control group [year, median (range)]	Type of study	Targeted therapy	Type of malignancy	Type of cutaneous adverse events	Cutaneous adverse events criteria	Type of intervention (preventive or reactive)	Way of administration	Intervention	Control	Dosage	Endpoint	Intervention duration	Follow-up duration/assessment
Bavetta et al. [12]	2021	Group A: 20 (20); Group B: 20 (20)	NA	Group A: 65.84§ (48–83); Group B: 70.63§ (50–81)	NA	prospective pilot study	afatinib, gefitinib, osimertinib, cetuximab, panitumumab	metastatic colorectal, lung, laryngeal and rectal cancer	papulopustular rash	NCI-CTCAE, DLQI, VAS	preventive (group B) and reactive (group A)	oral and topical	polydatin cream (additional minocycline)	no control	1.5% (for group A) and 0.8% (for group B and for group A after 4 weeks) polydatin cream topically applied twice daily for at least 12 consecutive weeks; preventive 100 mg	efficacy, QoL	12 weeks	every 4 weeks during the first 12 weeks (week 0, week 4, week 8, and week 12), and then after 12 more weeks (week 24)

Study	Year	Patient number of intervention group [ITT (PP)]	Patient number of control group [ITT (PP)]	Age of intervention group [year, median (range)]	Age of control group [year, median (range)]	Type of study	Targeted therapy	Type of malignancy	Type of cutaneous adverse events	Cutaneous adverse events criteria	Type of intervention (preventive or reactive)	Way of administration	Intervention	Control	Dosage	Endpoint	Intervention duration	Follow-up duration/assessment
															minocycline once a day			
Bidoli et al. [13]	2010	20 (7)	NA	NI	NA	preliminary study	erlotinib	advanced non-small cell lung cancer (NSCLC)	papulopustular, acne-like rash primarily affecting the face, scalp, and	NCI-CTCAE v3.0	reactive (grade ≥2)	oral	clindamycin, isotretinoin	no control	clindamycin 450 mg/day for days 1–10, then 300 mg/day for days 11–20 orally and isotretinoin 20 mg/day, days 11–20 orally	efficacy, compliance	20 days	on day 0, 15, and 30 with photographic documentation

Study	Year	Patient number of intervention group [ITT (PP)]	Patient number of control group [ITT (PP)]	Age of intervention group [year, median (range)]	Age of control group [year, median (range)]	Type of study	Targeted therapy	Type of malignancy	Type of cutaneous adverse events	Cutaneous adverse events criteria	Type of intervention (preventive or reactive)	Way of administration	Intervention	Control	Dosage	Endpoint	Intervention duration	Follow-up duration/assessment
									upper torso									
Bozkurt Duman et al. [14]	2011	10 (10)	NA	64±9 [§], 64 (44–76)	NA	preliminary clinical study	sorafenib	hepatocellular carcinoma (HCC)	hand-foot syndrome (HFS)	NCI-CTCAE v3.0	reactive	oral	Vitamin E	no control	vitamin E 300 mg once a day orally (without additional topical agents)	efficacy, compliance	continuous until HFS symptoms improved (noted within 10–	at days 15, 20–25, and 30–35, and monthly thereafter; HFS symptoms generally resolved to grade 1 or 0

Study	Year	Patient number of intervention group [ITT (PP)]	Patient number of control group [ITT (PP)]	Age of intervention group [year, median (range)]	Age of control group [year, median (range)]	Type of study	Targeted therapy	Type of malignancy	Type of cutaneous adverse events	Cutaneous adverse events criteria	Type of intervention (preventive or reactive)	Way of administration	Intervention	Control	Dosage	Endpoint	Intervention duration	Follow-up duration/assessment
																	12 days)	by day 20–25
Chayahara et al. [15]	2019	36 (26)	NA	65 (47–82) [†]	NA	randomized, evaluator-blinded, left-right comparative (APPEA	afatinib, gefitinib, cetuximab, panitumumab	head/neck, non-small cell lung, colorectal cancer	acneiform rash	NCI-CTCAE v4.0, IGA	preventive	oral and topical	adapalene gel 0.1% (additional minocycline, and moisturizer)	placebo (other half of face)	adapalene 0.1% gel topically once a day, oral minocycline and moisturizer	efficacy	4 weeks	at week 4 (from photographs)

Study	Year	Patient number of intervention group [ITT (PP)]	Patient number of control group [ITT (PP)]	Age of intervention group [year, median (range)]	Age of control group [year, median (range)]	Type of study	Targeted therapy	Type of malignancy	Type of cutaneous adverse events	Cutaneous adverse events criteria	Type of intervention (preventive or reactive)	Way of administration	Intervention	Control	Dosage	Endpoint	Intervention duration	Follow-up duration/assessment
						RANCE) phase 2 trial (UMIN000016692)												
Chen J.C. et al. [2]	2020	58 (58)	58 (58)	51.3 ± 11.7	57.4 ± 11.1	randomized, open-label,	sorafenib	hepatocellular carcinoma	hand-foot skin reaction, rash,	NCI-CTCAE 3.0	preventive	oral	celecoxib	no treatment	celecoxib: 200 mg twice daily (starting at the same time as sorafenib)	compliance	median 263.5 days	monthly

Study	Year	Patient number of intervention group [ITT (PP)]	Patient number of control group [ITT (PP)]	Age of intervention group [year, median (range)]	Age of control group [year, median (range)]	Type of study	Targeted therapy	Type of malignancy	Type of cutaneous adverse events	Cutaneous adverse events criteria	Type of intervention (preventive or reactive)	Way of administration	Intervention	Control	Dosage	Endpoint	Intervention duration	Follow-up duration/assessment
						prospective controlled clinical trial (NCT02961998)			alopecia									
De Noronha E	2009	19 (19)	NA	68 (47; 80)	NA	prospective descripti	erlotinib, cetuximab	metastatic non-smal	rash (acneiform erup	NCI-CTCAE	reactive (gr	oral and topical	systemic antibiotic (doxycycline, minocycl	no control	basic treatment: topical antibiotic (nadifloxacin,	efficacy, compliance	not detailed	biweekly and at week 4

Study	Year	Patient number of intervention group [ITT (PP)]	Patient number of control group [ITT (PP)]	Age of intervention group [year, median (range)]	Age of control group [year, median (range)]	Type of study	Targeted therapy	Type of malignancy	Type of cutaneous adverse events	Cutaneous adverse events criteria	Type of intervention (preventive or reactive)	Way of administration	Intervention	Control	Dosage	Endpoint	Intervention duration	Follow-up duration/assessment
Menezes et al. [16]						ve study		l cell lung, colorectal cancer	tion that was characterized by erythematous papular and pustular lesions)	v3.0	ade ≥2)		ine), additional systemic isotretinoin and antihistamine, topical antibiotic (nadifloxacin, clindamycin) or combined with benzoyl peroxide,		clindamycin) or combined with benzoyl peroxide, sunscreen, mild skin cleanser, oatmeal cream grade ≥2: plus systemic antibiotic (doxycycline, minocycline) 100 mg daily or low dose isotretinoin and			

Study	Year	Patient number of intervention group [ITT (PP)]	Patient number of control group [ITT (PP)]	Age of intervention group [year, median (range)]	Age of control group [year, median (range)]	Type of study	Targeted therapy	Type of malignancy	Type of cutaneous adverse events	Cutaneous adverse events criteria	Type of intervention (preventive or reactive)	Way of administration	Intervention	Control	Dosage	Endpoint	Intervention duration	Follow-up duration/assessment
													sunscreen, mild skin cleanser, oatmeal cream		antihistamines whenever pruritus was present			
Deplanque et al. [4]	2016	73 (21, month 4; 7, month 12)	74 (25, month 4; 11, month 12)	64 ± 11†§	64 ± 11†§	open-label, randomized, prospective, phase II	erlotinib	non-small cell lung cancer	folliculitis, skin lesions, xerosis, paronychia	NCI-CTCAE 3.0	preventive	oral	doxycycline	placebo	doxycycline: 100 mg/day	QoL, compliance	16 weeks	various (treatment period + 1-month post-treatment period; max. 13.5 months)

Study	Year	Patient number of intervention group [ITT (PP)]	Patient number of control group [ITT (PP)]	Age of intervention group [year, median (range)]	Age of control group [year, median (range)]	Type of study	Targeted therapy	Type of malignancy	Type of cutaneous adverse events	Cutaneous adverse events criteria	Type of intervention (preventive or reactive)	Way of administration	Intervention	Control	Dosage	Endpoint	Intervention duration	Follow-up duration/assessment
						trial (CYTAR) (NCT00531934)												. Evaluation at 14 days, 28 days and 2, 4, 7, 10, 12 months.
Fernández-Guarino et	2006	6 (6)	NA	59.5 ± 19,5§; 63.5 (31; 82)	NA	descriptive study	erlotinib, gefitinib	lung and metastatic renal adenocar	acneiform rash	NCI-CTCAE v3.0	reactive (grade ≥2)	oral	doxycycline	no control	100 mg doxycyline orally once a day	efficacy, compliance	3 weeks (6 weeks in 1 patient)	after 2-3 days, and week 1 and week 3 overall follow-up time:

Study	Year	Patient number of intervention group [ITT (PP)]	Patient number of control group [ITT (PP)]	Age of intervention group [year, median (range)]	Age of control group [year, median (range)]	Type of study	Targeted therapy	Type of malignancy	Type of cutaneous adverse events	Cutaneous adverse events criteria	Type of intervention (preventive or reactive)	Way of administration	Intervention	Control	Dosage	Endpoint	Intervention duration	Follow-up duration/assessment
al. [17]								cinoma										median 8.5 months (3-16)
Fuggetta et al. [18]	2019	34 (34)	NA	75%: >65; (25%) <65	NA	retrospective study	afatinib	non-small cell lung cancer (NSCLC)	rash (interfollicular and follicular-based erythematou	NCI-CTCAE v3.0	preventive	topical	1.5% Polydatin-based cream (GHIMAS) (with additional SPF 30 UVA/UVB non occlusive sunscreen)	no control (comparison with LUX-Lung 3/6 study)	topical application of polydatin-based cream twice daily on the face and body throughout targeted therapy	efficacy, compliance	from one day before starting afatinib treatment until the	weekly for the first month and after every 20 days or as needed

Study	Year	Patient number of intervention group [ITT (PP)]	Patient number of control group [ITT (PP)]	Age of intervention group [year, median (range)]	Age of control group [year, median (range)]	Type of study	Targeted therapy	Type of malignancy	Type of cutaneous adverse events	Cutaneous adverse events criteria	Type of intervention (preventive or reactive)	Way of administration	Intervention	Control	Dosage	Endpoint	Intervention duration	Follow-up duration/assessment
									s papules and pustules)								end of the antitumor therapy	
Gerber et al. [19]	2012	49 (49); group 1: 21, group 2: 23, grou	NA	NI	NA	retrospective, uncontrolled, comparative	erlotinib, cetuximab	colorectal, non-small cell lung, head and neck	papulopustular rash	ERSS or WoMoScore	reactive	oral and topical	mometasone furoate (group 1), prednicarbate + nadifloxacin (group	no control	mometason furoate topically twice a day for 3 weeks; nadifloxacin 1% topically once a day in the morning in	efficacy	3 weeks	after 3 weeks

Study	Year	Patient number of intervention group [ITT (PP)]	Patient number of control group [ITT (PP)]	Age of intervention group [year, median (range)]	Age of control group [year, median (range)]	Type of study	Targeted therapy	Type of malignancy	Type of cutaneous adverse events	Cutaneous adverse events criteria	Type of intervention (preventive or reactive)	Way of administration	Intervention	Control	Dosage	Endpoint	Intervention duration	Follow-up duration/assessment
		p 3: 5				study		cancers, renal cell carcinoma					2), prednicarbate + nadifloxacin + isotretinoin (group 3)		combination with prednicarbate 0.25% topically once a day in the evening for 3 weeks; patients with an ERSS>50 received nadifloxacin 1% and prednicarbate 0.25% topically in combination with the			

Study	Year	Patient number of intervention group [ITT (PP)]	Patient number of control group [ITT (PP)]	Age of intervention group [year, median (range)]	Age of control group [year, median (range)]	Type of study	Targeted therapy	Type of malignancy	Type of cutaneous adverse events	Cutaneous adverse events criteria	Type of intervention (preventive or reactive)	Way of administration	Intervention	Control	Dosage	Endpoint	Intervention duration	Follow-up duration/assessment
															systemic retinoid isotretinoin 10 to 20 mg/d for three weeks			
Goto et al. [20]	2018	35 (25); group 1: 6. group 2: 19	NA	66 (29-88)*	NA	retrospective observational study	afatinib	non-small cell lung cancer (NSCLC) with EGFR	rash, primarily occurring within a median of 7 days after	CTCAE v4.0	preventive and reactive (grade ≥1)	oral and topical	prevention (grade 0) (group 1): minocycline (additional moisturizing agents,	no control	minocycline 50 mg twice a day (100 mg/day) orally used, started within a median of 8 days after afatinib initiation, combined with	efficacy, compliance	median 241 days (95 % CI: 89–325)	229 days (range: 24–763)

Study	Year	Patient number of intervention group [ITT (PP)]	Patient number of control group [ITT (PP)]	Age of intervention group [year, median (range)]	Age of control group [year, median (range)]	Type of study	Targeted therapy	Type of malignancy	Type of cutaneous adverse events	Cutaneous adverse events criteria	Type of intervention (preventive or reactive)	Way of administration	Intervention	Control	Dosage	Endpoint	Intervention duration	Follow-up duration/assessment
								mutations	afatinib initiation, paronychia				sunblock); reactive (grade ≥1) (group 2): minocycline, topical corticosteroids		moisturizing agents and topical corticosteroids for some patients			
Grandeet	2013	51 (51)	NA	67 (34-82)	NA	phase II study	erlotinib, cetuximab, panitu	metastatic colorecta	papulopustular rash,	NCI-CTCAE	preventive	oral and topical	lymecycline, skin moisturizers and	no control	300 mg lymecycline orally once a day; moisturizers	efficacy, QoL	12 weeks (3 months)	every 2 weeks for 3 months then till

Study	Year	Patient number of intervention group [ITT (PP)]	Patient number of control group [ITT (PP)]	Age of intervention group [year, median (range)]	Age of control group [year, median (range)]	Type of study	Targeted therapy	Type of malignancy	Type of cutaneous adverse events	Cutaneous adverse events criteria	Type of intervention (preventive or reactive)	Way of administration	Intervention	Control	Dosage	Endpoint	Intervention duration	Follow-up duration/assessment
al. [21]							mumab	l, metastatic lung cancer	xerosis, paronychia	v3.0; DLQI,			sunscreen		and sunscreen applied to face and neck two times a day; moisturizers with salicylic acid and sulfur applied to back and chest daily; hand and feet cream with hyaluronic acid and phytosterols			the end of the anti-EGFR therapy (median 4 months, range: 2-17)

Study	Year	Patient number of intervention group [ITT (PP)]	Patient number of control group [ITT (PP)]	Age of intervention group [year, median (range)]	Age of control group [year, median (range)]	Type of study	Targeted therapy	Type of malignancy	Type of cutaneous adverse events	Cutaneous adverse events criteria	Type of intervention (preventive or reactive)	Way of administration	Intervention	Control	Dosage	Endpoint	Intervention duration	Follow-up duration/assessment
															applied two times a day			
Hwang et al. [22]	2016	52 (46)	NA	61 (40-83)*	NA	open-label, non-comparative, phase II trial (NCT01593995)	erlotinib	non-small cell lung cancer (NSCLC) and pancreatic cancer (PC)	rash/desquamation, rash/acne, xerosis, pruritus, paronychia	NCI-CTCAE v3.0, SKINDEX-16	reactive (grade ≥2)	topical	Epidermal Growth Factor (EGF) ointment (Daewoong Pharmaceuticals), containing 1 ppm of nepidermin	no control	topical, applied twice daily (oral and intravenous antibiotics, antihistamine drugs, and steroids was allowed, topical not – not detailed)	efficacy, QoL	8 weeks	according to the chemotherapy schedule every 3 or 4 weeks, but was not assessed until at least 1 week after the

Study	Year	Patient number of intervention group [ITT (PP)]	Patient number of control group [ITT (PP)]	Age of intervention group [year, median (range)]	Age of control group [year, median (range)]	Type of study	Targeted therapy	Type of malignancy	Type of cutaneous adverse events	Cutaneous adverse events criteria	Type of intervention (preventive or reactive)	Way of administration	Intervention	Control	Dosage	Endpoint	Intervention duration	Follow-up duration/assessment
																		application of the ointment
Ichiki et al. [23]	2017	29 (29)	NA	66 (45-85)	NA	prospective phase II study (UMIN00018392)	afatinib	non-small cell lung cancer (NSCLC)	oral mucositis, skin rash, paronychia	NCI-CTCAE v4.0	preventive	oral and topical	Kampo medicine (TJ-14), minocycline	no control (comparison with LUX-Lung 3/6	TJ-14: rinse and gargle 2.5 g of TJ-14 in water three times daily; Minocycline: 50 mg orally twice daily: Moisturizing agent (Hirudoid®)	efficacy	at least 4 weeks (range: 4–91 weeks)	at weeks 1, 2, and 4 after the initiation of afatinib, followed by regular intervals

Study	Year	Patient number of intervention group [ITT (PP)]	Patient number of control group [ITT (PP)]	Age of intervention group [year, median (range)]	Age of control group [year, median (range)]	Type of study	Targeted therapy	Type of malignancy	Type of cutaneous adverse events	Cutaneous adverse events criteria	Type of intervention (preventive or reactive)	Way of administration	Intervention	Control	Dosage	Endpoint	Intervention duration	Follow-up duration/assessment
														study)	topically twice a day + >30 SPF sunscreen (prophylactic topical or systemic steroid therapy was not allowed)			
Iimura et al. [24]	2018	49 (20); group 1: 12, group 2: 5,	NA	69 (37-89) †	NA	retrospective study (UMIN0003	gefitinib, erlotinib, afatinib, osimertinib	non-small cell lung cancer (NS	rash (the face and upper body)	NCI-CTCAE v3.0	preventive	oral and topical	non-steroidal anti-inflammatory drugs (NSAIDs) (group	no control	loxoprofen 60 mg orally 2-3 times a day	efficacy	NI	NI

Study	Year	Patient number of intervention group [ITT (PP)]	Patient number of control group [ITT (PP)]	Age of intervention group [year, median (range)]	Age of control group [year, median (range)]	Type of study	Targeted therapy	Type of malignancy	Type of cutaneous adverse events	Cutaneous adverse events criteria	Type of intervention (preventive or reactive)	Way of administration	Intervention	Control	Dosage	Endpoint	Intervention duration	Follow-up duration/assessment
		group 3: 3				0657)		CLC)					1), specifically loxoprofen; steroid (group 2); acetylsalicylic acid (group 3) Additional basic skin care with sunscreen,					

Study	Year	Patient number of intervention group [ITT (PP)]	Patient number of control group [ITT (PP)]	Age of intervention group [year, median (range)]	Age of control group [year, median (range)]	Type of study	Targeted therapy	Type of malignancy	Type of cutaneous adverse events	Cutaneous adverse events criteria	Type of intervention (preventive or reactive)	Way of administration	Intervention	Control	Dosage	Endpoint	Intervention duration	Follow-up duration/assessment
													moisturizer, and topical 1% hydrocortisone.					
Ishikawa et al. [25]	2017	60 (55)	NA	64 (38-78)*	NA	Retrospective study	regorafenib	colorectal cancer	hand-foot skin reaction (HFSR)	NCI-CTCAE v3.0	Preventive	topical	topical steroids (0.05% diflupred nate) and 20% urea-based cream	no control, historical comparison with CORRECT	applied to hands and feet twice daily (morning and evening) from treatment start	efficacy, compliance	throughout the regorafenib treatment cycles (me	NI

Study	Year	Patient number of intervention group [ITT (PP)]	Patient number of control group [ITT (PP)]	Age of intervention group [year, median (range)]	Age of control group [year, median (range)]	Type of study	Targeted therapy	Type of malignancy	Type of cutaneous adverse events	Cutaneous adverse events criteria	Type of intervention (preventive or reactive)	Way of administration	Intervention	Control	Dosage	Endpoint	Intervention duration	Follow-up duration/assessment
														study (Japanese subgroup)			dian: 7.1 weeks)	
Iwasaku et al. [26, 27]	2019; 2023	41 (41)	NA	70 (32-83)	NA	prospective, open-label, phase II trial	dacomitinib	non-small-cell lung cancer (NSCLC)	acneiform rash, dry skin, pruritus, paronych	NCI-CTCAE v5.0	preventive	oral and topical	minocycline, heparinoid moisturizer, medium and very strong topical	no control group; historical comparison	minocycline: orally 100 mg once daily; heparinoid skin moisturizer: twice daily for all areas; Topical	efficacy, compliance	8 weeks (primary evaluation peri	biweekly during the 8-week period

Study	Year	Patient number of intervention group [ITT (PP)]	Patient number of control group [ITT (PP)]	Age of intervention group [year, median (range)]	Age of control group [year, median (range)]	Type of study	Targeted therapy	Type of malignancy	Type of cutaneous adverse events	Cutaneous adverse events criteria	Type of intervention (preventive or reactive)	Way of administration	Intervention	Control	Dosage	Endpoint	Intervention duration	Follow-up duration/assessment
						(SPIRAL-Daco study, jRCTs071190015)			ia, maculopapular rash, erythema multiforme, HSFR, skin infection,				steroids, sunscreen	with ARCHER1050 and ARCHER1042 trials	steroids: twice daily for face (class IV) and other areas (class II); Sunscreen: SPF ≥ 25 (during outdoor activities)		od); patients continued as needed beyond this period, median: 22 days	

Study	Year	Patient number of intervention group [ITT (PP)]	Patient number of control group [ITT (PP)]	Age of intervention group [year, median (range)]	Age of control group [year, median (range)]	Type of study	Targeted therapy	Type of malignancy	Type of cutaneous adverse events	Cutaneous adverse events criteria	Type of intervention (preventive or reactive)	Way of administration	Intervention	Control	Dosage	Endpoint	Intervention duration	Follow-up duration/assessment
									eczema								(8-52)	
Jatoi et al. [29]	2008	31 (30, 15)	30 (29,12)	71 (40-84) [†]	63 (49-84) [†]	randomized, placebo-controlled, double-blinded , phase III study	gefitinib, erlotinib, cetuximab	lung, gastrointestinal and other cancer (not specified)	papulopustular rash	NCI-CTCAE v3.0, SKINDEX-16	preventive	oral	tetracycline	placebo	500 mg tetracycline orally twice a day (supportive treatments was allowed but not detailed)	efficacy	4 weeks	week 4 and week 8 (monthly physician assessment and weekly patient-reported questionnaires)

Study	Year	Patient number of intervention group [ITT (PP)]	Patient number of control group [ITT (PP)]	Age of intervention group [year, median (range)]	Age of control group [year, median (range)]	Type of study	Targeted therapy	Type of malignancy	Type of cutaneous adverse events	Cutaneous adverse events criteria	Type of intervention (preventive or reactive)	Way of administration	Intervention	Control	Dosage	Endpoint	Intervention duration	Follow-up duration/assessment
Jatoi et al. [28]	2021	61 (61) (preventive)	55 (55) (reactive)	63 (53, 68)	61 (53 , 69)	pre-planned analysis of the ReDOS randomized four-arm, phase II trial (NC	regorafenib	metastatic colorectal cancer	hand-foot skin reaction (HFSR)	NCI-CTCAE v4.0	preventive and reactive	topical	0.05% clobetasol cream, applied preemptively (before HFSR development)	0.05 % clobetasol cream, applied reactively (after HFSR onset)	applied as a thin layer to palms and soles twice daily over two cycles (2 x 28 days) following these: (1) preemptive clobetasol 80 mg/day with potential escalation to 160 mg/day; (2) reactive clobetasol	efficacy, QoL	NI	NI

Study	Year	Patient number of intervention group [ITT (PP)]	Patient number of control group [ITT (PP)]	Age of intervention group [year, median (range)]	Age of control group [year, median (range)]	Type of study	Targeted therapy	Type of malignancy	Type of cutaneous adverse events	Cutaneous adverse events criteria	Type of intervention (preventive or reactive)	Way of administration	Intervention	Control	Dosage	Endpoint	Intervention duration	Follow-up duration/assessment
						T02368886)									starting dose 80 mg/day with potential dose escalation to 160 mg/day; (3) preemptive clobetasol 160 mg starting dose; (4) reactive clobetasol 160 mg starting dose			

Study	Year	Patient number of intervention group [ITT (PP)]	Patient number of control group [ITT (PP)]	Age of intervention group [year, median (range)]	Age of control group [year, median (range)]	Type of study	Targeted therapy	Type of malignancy	Type of cutaneous adverse events	Cutaneous adverse events criteria	Type of intervention (preventive or reactive)	Way of administration	Intervention	Control	Dosage	Endpoint	Intervention duration	Follow-up duration/assessment
Jornsamer et al. [30]	2022	8 (8)	8 (8)	64 (56-75)	64 (56-75)	randomized, controlled study	erlotinib, afatinib, gefitinib, dacomitinib, and osimertinib	lung cancer	paronychia	SPOT (Scoring system for Paronychia Related Oncol	reactive	topical	red light-emitting diode (LED) therapy at 633 nm wavelength, combined with standard treatments (e.g.: Proper nail care, daily iodopovi	the untreated extremities serves as control (standard treatment alone)	LED therapy applied to affected nails 2–3 times weekly for 6 weeks, 20 minutes per session (126 J/cm² dose per session)	efficacy, QoL	6 weeks	weeks 0, 2, 4, 6, 8

Study	Year	Patient number of intervention group [ITT (PP)]	Patient number of control group [ITT (PP)]	Age of intervention group [year, median (range)]	Age of control group [year, median (range)]	Type of study	Targeted therapy	Type of malignancy	Type of cutaneous adverse events	Cutaneous adverse events criteria	Type of intervention (preventive or reactive)	Way of administration	Intervention	Control	Dosage	Endpoint	Intervention duration	Follow-up duration/assessment
										ogic Treatment) and a visual analog pain scale			done solution soaks, topical and oral antibiotics, topical corticosteroids, nail surgery, and phenol chemical matricectomy – not detailed)					

Study	Year	Patient number of intervention group [ITT (PP)]	Patient number of control group [ITT (PP)]	Age of intervention group [year, median (range)]	Age of control group [year, median (range)]	Type of study	Targeted therapy	Type of malignancy	Type of cutaneous adverse events	Cutaneous adverse events criteria	Type of intervention (preventive or reactive)	Way of administration	Intervention	Control	Dosage	Endpoint	Intervention duration	Follow-up duration/assessment
Kanazawa et al. [31]	2006	12 (12)	28 (27)	69 (48-80)	64 (42-82)	prospective nonrandomized controlled study	gefitinib	non-small cell lung cancer	hand-foot skin reaction	Not specified	preventive	oral	acetylsalicylic acid	placebo	acetylsalicylic acid: 100 mg/day	efficacy	2 weeks	not specified
Kim E. J. et	2020	29 (29)	NA	61 (31-77)	NA	open-label phase II stud	imatinib (dose ranged prima	gastrointestinal stromal	erythematous maculop	NCI-CTCAE	reactive; (grade ≥2)	oral	prednisolone (additional antihistamines	no control	oral prednisolone, starting at 30 mg/day for 3 weeks, then gradually	efficacy/compliance	15 weeks	every 3 weeks during steroid treatment with

Study	Year	Patient number of intervention group [ITT (PP)]	Patient number of control group [ITT (PP)]	Age of intervention group [year, median (range)]	Age of control group [year, median (range)]	Type of study	Targeted therapy	Type of malignancy	Type of cutaneous adverse events	Cutaneous adverse events criteria	Type of intervention (preventive or reactive)	Way of administration	Intervention	Control	Dosage	Endpoint	Intervention duration	Follow-up duration/assessment
al. [32]						y (NCT03440515)	rily from 300–600 mg daily)	tumors (GIST)	apular rash with pruritus	v4.03			could be used)		tapered over the next 12 weeks (gradual reduction every 3 weeks until the 15th week)			extended follow-up (median was 2 years)
Kim Y.S. et al. [33]	2020	Arm 2: 26 (25); Arm 3: 27 (27)	Arm 1: 27 (23)	Arm 2: 64 (30-79)†; Arm 3: 56 (42-83)†	Arm 1: 57 (43-83)†	randomized, placebo-controlled, doub	afatinib, erlotinib, gefitinib, cetuximab	non-small cell lung, pancreatic and colo	hand-foot syndrome, acneiform rash,	NCI-CTCAE v4.0, SKIN	reactive (grade ≥2)	topical	epidermal growth factor (EGF) ointment: EGF 1 ppm (arm 2); EGF 20	placebo (arm 1)	Arm 2: 1 ppm of EGF topically twice a day Arm 3: 20 ppm of EGF topically twice a day.	efficacy, QoL	at least 1 week	at 2 weeks after the treatment and every 4 weeks thereaft

Study	Year	Patient number of intervention group [ITT (PP)]	Patient number of control group [ITT (PP)]	Age of intervention group [year, median (range)]	Age of control group [year, median (range)]	Type of study	Targeted therapy	Type of malignancy	Type of cutaneous adverse events	Cutaneous adverse events criteria	Type of intervention (preventive or reactive)	Way of administration	Intervention	Control	Dosage	Endpoint	Intervention duration	Follow-up duration/assessment
						le-blind, pilot phase III trial (NCT02284139)		rectal cancer	dry skin, pruritus, paronychia	DEX-16			ppm (arm 3)		(other oral and intravenous – topical not - supplemental medication was allowed, if it was needed – but not detailed)			er until week 8
Lacouture et al. [5]	2016	cohort I: 66 (56); cohort II:	66 (58)	65.5 (36–88)*	67.0 (41–85)*	triple cohort, randomized,	dacomitinib	non-small cell lung cancer	rash, dry skin, dermatitis acne	NCI-CTCAE 4.0,	preventive	oral and topical	doxycycline (cohort I) or alclometasone	placebo	doxycycline: 100 mg twice a day for 4 weeks; alclometasone dipropionate	QoL	4 weeks	8 + 4 weeks

Study	Year	Patient number of intervention group [ITT (PP)]	Patient number of control group [ITT (PP)]	Age of intervention group [year, median (range)]	Age of control group [year, median (range)]	Type of study	Targeted therapy	Type of malignancy	Type of cutaneous adverse events	Cutaneous adverse events criteria	Type of intervention (preventive or reactive)	Way of administration	Intervention	Control	Dosage	Endpoint	Intervention duration	Follow-up duration/assessment
		72 (59)				phase II clinical study (ARCHER 1042) (NCT01465802)			iform, paronychia, rash maculopapular, rash pustular, fissure	SKINDEX-16			(cohort II)		cream 0.05% bedtime daily for 4 weeks (to face, hands, feet, neck, back, and chest)			
Li et	20	26 (26)	NA	66 (46-87)	NA	prospective	sunitinib, sorafe	metastatic	hand-foot skin	NCI-CT	reactive	topical	Topical heparin Z	no control	Topical application twice daily	efficacy, com	variable, until	weekly during treatme

Study	Year	Patient number of intervention group [ITT (PP)]	Patient number of control group [ITT (PP)]	Age of intervention group [year, median (range)]	Age of control group [year, median (range)]	Type of study	Targeted therapy	Type of malignancy	Type of cutaneous adverse events	Cutaneous adverse events criteria	Type of intervention (preventive or reactive)	Way of administration	Intervention	Control	Dosage	Endpoint	Intervention duration	Follow-up duration/assessment
al. [35]	13					non-randomized observational study	nib, axitinib	renal cell carcinoma (MRCC) and castration-resistant prostate cancer	reaction (HFSR)	CAE v4.0	(grade ≥1)		ointment (500 IU/1 g), combined with urea-based cream		until improvement	pliance	improvement	nt and monthly during follow-up visits

Study	Year	Patient number of intervention group [ITT (PP)]	Patient number of control group [ITT (PP)]	Age of intervention group [year, median (range)]	Age of control group [year, median (range)]	Type of study	Targeted therapy	Type of malignancy	Type of cutaneous adverse events	Cutaneous adverse events criteria	Type of intervention (preventive or reactive)	Way of administration	Intervention	Control	Dosage	Endpoint	Intervention duration	Follow-up duration/assessment
								(CRPC)										
Li et al. [34]	2022	14 (7)	16 (14)	67.3 ± 14.8[†§]; 63.1 ± 15.8[*§]	64.8 ± 15.2[†§]; 64.0 ± 16.1[*§]	prospective, randomized pilot study	afatinib	Lung adenocarcinoma	skin toxicity, paronychia, acneiform rash, rash maculopapular	NCI-CTCAE v4.03	preventive	oral	traditional Chinese medicine	placebo	3 packets of the powdered herbal mixture three times a day	efficacy, QoL	12 weeks	18 weeks

Study	Year	Patient number of intervention group [ITT (PP)]	Patient number of control group [ITT (PP)]	Age of intervention group [year, median (range)]	Age of control group [year, median (range)]	Type of study	Targeted therapy	Type of malignancy	Type of cutaneous adverse events	Cutaneous adverse events criteria	Type of intervention (preventive or reactive)	Way of administration	Intervention	Control	Dosage	Endpoint	Intervention duration	Follow-up duration/assessment
Liu T. H. et al. [36]	2022	11 (11)	NA	72±10.95 § (range: 57-94)	NA	retrospective cohort study (A20210 5066)	afatinib, gefitinib, erlotinib, osimertinib	non-small cell lung cancer (NSCLC)	paronychia	NCI-CTCAE v5.0	reactive;	topical	surgical partial matricectomy with electrocautery ablation of the germinal matrix, combined with paraffin and framycetin dressing	conservative treatment effectiveness: topical beta-blockers, topical povidone –	procedure performed under local anesthesia; surgical intervention to remove the affected portion of the nail matrix and ablate tissue	efficacy	variable (surgical: 3.22 ± 1.00 weeks; conservative: 56.56 ± 52.29 weeks)	variable

**Study**	**Year**	**Patient number of intervention group [ITT (PP)]**	**Patient number of control group [ITT (PP)]**	**Age of intervention group [year, median (range)]**	**Age of control group [year, median (range)]**	**Type of study**	**Targeted therapy**	**Type of malignancy**	**Type of cutaneous adverse events**	**Cutaneous adverse events criteria**	**Type of intervention (preventive or reactive)**	**Way of administration**	**Intervention**	**Control**	**Dosage**	**Endpoint**	**Intervention duration**	**Follow-up duration/assessment**
														iodine, topical antibiotic ointment, and steroid ointment				
Liu Z. et al. [37]	2022	Arm A: 46 (46)	Arm C: 46	Arm A: 53.2 ±21.1[§]	Arm C: 54.7±	prospective, open-	erlotinib, gefitinib, icotini	metastatic colorecta	acneiform rash,	NCI-CTCAE	preventive and	oral and topical	**Preventive** (Arm A): honeysuckle.	**Reactive 2** (Arm C	**Preventive** (Arm A): 10g in 200 ml honeysuckle	efficacy	until recovery	every 2-3 weeks and followed for at

Study	Year	Patient number of intervention group [ITT (PP)]	Patient number of control group [ITT (PP)]	Age of intervention group [year, median (range)]	Age of control group [year, median (range)]	Type of study	Targeted therapy	Type of malignancy	Type of cutaneous adverse events	Cutaneous adverse events criteria	Type of intervention (preventive or reactive)	Way of administration	Intervention	Control	Dosage	Endpoint	Intervention duration	Follow-up duration/assessment
		Arm B: 47 (47)	(46)	Arm B: 49.5 ±20.5 §	19.9§	label, randomized, controlled clinical trial	b, cetuximab	l and non-small cell lung cancer	pruritus	v4.03	reactive		**Reactive 1** (Arm B): honeysuckle.	-conventional treatment): minocycline, clindamycin and hydrocortisone	soup orally twice a day. **Reactive 1** (Arm B): when grade ≥1 rash occurred, honeysuckle orally (twice a day) and external (50 g in 1000 ml water; three times a day). **Reactive 2** (Arm C): when grade ≥1 rash			least 6 months

Study	Year	Patient number of intervention group [ITT (PP)]	Patient number of control group [ITT (PP)]	Age of intervention group [year, median (range)]	Age of control group [year, median (range)]	Type of study	Targeted therapy	Type of malignancy	Type of cutaneous adverse events	Cutaneous adverse events criteria	Type of intervention (preventive or reactive)	Way of administration	Intervention	Control	Dosage	Endpoint	Intervention duration	Follow-up duration/assessment
															occurred, 100 mg minocycline orally once a day; 2% clindamycin and 1% hydrocortisone solution topically twice a day			
Lu et al. [38]	2021	40 (40)	91 (91)	62.9 ±13.0[§] (range: 37-81)	64.9± X[§] (range: 40-	case-control cohort study	afatinib, erlotinib, gefitinib	non-small cell lung cancer (NS	paronychia	NCI-CTCAE v5.0,	preventive	topical	betaxolol 0.25% solution	no treatment	betaxolol 0.25% topically applied to nails twice a day	efficacy	continuous application duri	at baseline and week 1, 2, 4, 8, 12

Study	Year	Patient number of intervention group [ITT (PP)]	Patient number of control group [ITT (PP)]	Age of intervention group [year, median (range)]	Age of control group [year, median (range)]	Type of study	Targeted therapy	Type of malignancy	Type of cutaneous adverse events	Cutaneous adverse events criteria	Type of intervention (preventive or reactive)	Way of administration	Intervention	Control	Dosage	Endpoint	Intervention duration	Follow-up duration/assessment
					91)†			CLC)		VAS score					(For any patients who developed any grade of paronychia in the prevention group or control group, the conventional therapies such as topical antibiotics, topical silver nitrate, or systemic		ng the first 3 months of EGFR-TKI therapy	

Study	Year	Patient number of intervention group [ITT (PP)]	Patient number of control group [ITT (PP)]	Age of intervention group [year, median (range)]	Age of control group [year, median (range)]	Type of study	Targeted therapy	Type of malignancy	Type of cutaneous adverse events	Cutaneous adverse events criteria	Type of intervention (preventive or reactive)	Way of administration	Intervention	Control	Dosage	Endpoint	Intervention duration	Follow-up duration/assessment
															antibiotics were used for the management of the paronychia.)			
Masago et al. [39]	2018	51 (preintervention cohort: 32; postintervention	NA	NI	NI	retrospective cohort study	erlotinib, gefitinib, afatinib	non-small cell lung cancer (NSCLC) with somatic	acneiform rash, dry skin, paronychia, and pruritus	NCI-CTCAE v4.0, DLQI, SKINDE	preventive	oral and topical	multimodal team management – pre-intervention	multimodal team management – post-intervention	multimodal approach with oral minocycline (as needed, when topical agents were uneffective), topical alclometasone dipropionate	efficacy, QoL	continuous during EGFR-TKI therapy.	at week 1, 2, 4, then every 4 weeks after beginning the treatment with the

Study	Year	Patient number of intervention group [ITT (PP)]	Patient number of control group [ITT (PP)]	Age of intervention group [year, median (range)]	Age of control group [year, median (range)]	Type of study	Targeted therapy	Type of malignancy	Type of cutaneous adverse events	Cutaneous adverse events criteria	Type of intervention (preventive or reactive)	Way of administration	Intervention	Control	Dosage	Endpoint	Intervention duration	Follow-up duration/assessment
		cohort: 27)						epidermal growth factor receptor (EGFR) mutations		X-29					(for the face), difluprednate (for the body), betamethasone valerate (for the scalp), and moisturizing agents			EGFR-TKI
Meadows et	2015	10 (9)	NA	58.5 (43-74)*	NA	randomized, double-	sunitinib, bevacizumab,	colorectal, renal,	hand-foot syndrome	NCI-CTCAE	reactive (grade	topical	1% topical sildenafil cream	placebo (contrala	0.5 mL of 1% sildenafil topically to either the right or left	efficacy	7 days	self-evaluation with a patient diary

Study	Year	Patient number of intervention group [ITT (PP)]	Patient number of control group [ITT (PP)]	Age of intervention group [year, median (range)]	Age of control group [year, median (range)]	Type of study	Targeted therapy	Type of malignancy	Type of cutaneous adverse events	Cutaneous adverse events criteria	Type of intervention (preventive or reactive)	Way of administration	Intervention	Control	Dosage	Endpoint	Intervention duration	Follow-up duration/assessment
al. [40]						blind, placebo-controlled (self controlled) pilot study (NCT01219023)	capecitabine	breast, pancreatic cancer, gastrointestinal stromal tumors, angiosar		v4.0	1–3)			teral side)	extremity (hand/foot) and placebo to the opposite extremity, twice a day. Standard emollients was encouraged as part of standard care applying at least approximately 2 hours after			card (not detailed)

Study	Year	Patient number of intervention group [ITT (PP)]	Patient number of control group [ITT (PP)]	Age of intervention group [year, median (range)]	Age of control group [year, median (range)]	Type of study	Targeted therapy	Type of malignancy	Type of cutaneous adverse events	Cutaneous adverse events criteria	Type of intervention (preventive or reactive)	Way of administration	Intervention	Control	Dosage	Endpoint	Intervention duration	Follow-up duration/assessment
								coma							administration of topical sildenafil.			
Melosky et al. [8]	2016	50 (50)	50 (50)	64.9 (for all population)		randomized, three-arm phase III trial	erlotinib	Non-small cell lung cancer	papulopustular/acneiform rash	adapted grading system developed by Perez-	reactive	oral	clindamycin + hidrocortisone	preventive treatment or no treatment	topical clindamycin plus hydrocortisone with or without minocycline at rash occurrence per grade	efficacy	4 weeks	12 weeks

Study	Year	Patient number of intervention group [ITT (PP)]	Patient number of control group [ITT (PP)]	Age of intervention group [year, median (range)]	Age of control group [year, median (range)]	Type of study	Targeted therapy	Type of malignancy	Type of cutaneous adverse events	Cutaneous adverse events criteria	Type of intervention (preventive or reactive)	Way of administration	Intervention	Control	Dosage	Endpoint	Intervention duration	Follow-up duration/assessment
										Soler et al., based on NCI-CTCAE v3.0								
Melosky et	2019	10 (10)	NA	60.65 (49-72)	NA	open-label,	regorafenib	metastatic colo	hand-foot synd	NCI-CTCA	preventive	oral	perindopril	no control	perindopril 4 mg orally once a day	efficacy	21 days of treat	weekly evaluations during

Study	Year	Patient number of intervention group [ITT (PP)]	Patient number of control group [ITT (PP)]	Age of intervention group [year, median (range)]	Age of control group [year, median (range)]	Type of study	Targeted therapy	Type of malignancy	Type of cutaneous adverse events	Cutaneous adverse events criteria	Type of intervention (preventive or reactive)	Way of administration	Intervention	Control	Dosage	Endpoint	Intervention duration	Follow-up duration/assessment
al. [41]						phase II trial (NCT02651415)		rectal cancer	rome	E v4.03							ment per 28-day cycle	the first cycle, then biweekly during the second cycle, at the start of each subsequent cycle, at the end of treatment, and every

Study	Year	Patient number of intervention group [ITT (PP)]	Patient number of control group [ITT (PP)]	Age of intervention group [year, median (range)]	Age of control group [year, median (range)]	Type of study	Targeted therapy	Type of malignancy	Type of cutaneous adverse events	Cutaneous adverse events criteria	Type of intervention (preventive or reactive)	Way of administration	Intervention	Control	Dosage	Endpoint	Intervention duration	Follow-up duration/assessment
																		month after cessation of treatment until death or data cutoff
Naganuma et al. [42]	2019	28 (25)	25 (25)	72.4 ± 12.1 § (range: 34–85) †	68.2 ± 7.6 §; (range: 51 –	prospective, non-randomized controlle	sorafenib	Hepatocellular carcinoma	hand-foot skin reaction, rash, dry skin, eryt	NCI-CTCAE v4.0	preventive	oral	oral nutritional supplement (ONS) (HMB, L-arginine,	historical control	ONS: 1 packet/day; Additional treatment: daily skin care (10% urea cream, cotton	efficacy	12 weeks	12 weeks

Study	Year	Patient number of intervention group [ITT (PP)]	Patient number of control group [ITT (PP)]	Age of intervention group [year, median (range)]	Age of control group [year, median (range)]	Type of study	Targeted therapy	Type of malignancy	Type of cutaneous adverse events	Cutaneous adverse events criteria	Type of intervention (preventive or reactive)	Way of administration	Intervention	Control	Dosage	Endpoint	Intervention duration	Follow-up duration/assessment
					87) †	d trial			hema				L-glutamine)		gloves/socks, etc.)			
Nishino et al. [43]	2021	15 (15) (reactive phase)	6 (6) (reactive phase)	58 (45–78)	65 (52–76)	phase III, open-label, randomized controlled trial (RCT -	erlotinib, afatinib	non-small cell lung cancer (NSCLC)	facial acneiform rash	adapted grading system developed by Scope	reactive (firstly preventive, and if facial acneif	topical	corticosteroid (DOWN-group: ranking-down corticosteroid treatment strategy)	corticosteroid (WEAK-group): ranking-up corticosteroid	100-200 mg minocycline orally once a day for 2 weeks and heparinoid moisturizer topically as preventive therapy; if rash was observed, corticosteroids was added topically	efficacy, compliance	preventive duration: 2 weeks; reactive duration: 8 weeks	biweekly throughout the 10-week study duration

Study	Year	Patient number of intervention group [ITT (PP)]	Patient number of control group [ITT (PP)]	Age of intervention group [year, median (range)]	Age of control group [year, median (range)]	Type of study	Targeted therapy	Type of malignancy	Type of cutaneous adverse events	Cutaneous adverse events criteria	Type of intervention (preventive or reactive)	Way of administration	Intervention	Control	Dosage	Endpoint	Intervention duration	Follow-up duration/assessment
						FAEISS) (UMIN000024113)				et al.	orm rash is developed within 2 weeks, than second			treatment strategies	including ranking-up (WEAK) or ranking-down (DOWN) corticosteroid protocols		(DOWN group de-escalates from strong to weak, WEAK group escalates from	

Study	Year	Patient number of intervention group [ITT (PP)]	Patient number of control group [ITT (PP)]	Age of intervention group [year, median (range)]	Age of control group [year, median (range)]	Type of study	Targeted therapy	Type of malignancy	Type of cutaneous adverse events	Cutaneous adverse events criteria	Type of intervention (preventive or reactive)	Way of administration	Intervention	Control	Dosage	Endpoint	Intervention duration	Follow-up duration/assessment
											registration of trial is started with reactive intervent						weak to strong biweekly)	

Study	Year	Patient number of intervention group [ITT (PP)]	Patient number of control group [ITT (PP)]	Age of intervention group [year, median (range)]	Age of control group [year, median (range)]	Type of study	Targeted therapy	Type of malignancy	Type of cutaneous adverse events	Cutaneous adverse events criteria	Type of intervention (preventive or reactive)	Way of administration	Intervention	Control	Dosage	Endpoint	Intervention duration	Follow-up duration/assessment
											ion)							
Nishizawa et al. [44]	2023	28 (27)	NA	56.5 (41-81)†	NA	nonrandomized, observational (jRCTs031180096)	regorafenib	metastatic colorectal cancer (mCRC)	hand-foot skin reaction (HFSR)	NCI-CTCAE v4.0	preventive	topical	aluminum chloride ointment (30%)	no control	aluminium chloride topically applied one week before regorafenib treatment	efficacy, compliance	12 weeks	weekly during the first cycle, then biweekly thereafter for week 12 or until treatment discontinuation

Study	Year	Patient number of intervention group [ITT (PP)]	Patient number of control group [ITT (PP)]	Age of intervention group [year, median (range)]	Age of control group [year, median (range)]	Type of study	Targeted therapy	Type of malignancy	Type of cutaneous adverse events	Cutaneous adverse events criteria	Type of intervention (preventive or reactive)	Way of administration	Intervention	Control	Dosage	Endpoint	Intervention duration	Follow-up duration/assessment
Okajima et al. [45]	2021	46 (35)	NA	65 (36–83)†; 65 (36-79)*	NA	prospective phase II study (UMIN000016167)	afatinib	advanced non-small cell lung cancer (NSCLC) with EGFR mutation	rash/acne, paronychia, and oral mucositis	NCI-CTCAE (not specified)	Preventive	oral and topical	minocycline, topical medium-class steroids or sodium azulene gargle	no control	• minocycline (100 mg/day) + medium-grade topical steroids for rash/paronychia<br>• sodium azulene gargle (four times daily) for oral mucositis	efficacy	variable, continued until disease progression or until intolerable toxicity	daily for 12 weeks (questionnaire, photography)

Study	Year	Patient number of intervention group [ITT (PP)]	Patient number of control group [ITT (PP)]	Age of intervention group [year, median (range)]	Age of control group [year, median (range)]	Type of study	Targeted therapy	Type of malignancy	Type of cutaneous adverse events	Cutaneous adverse events criteria	Type of intervention (preventive or reactive)	Way of administration	Intervention	Control	Dosage	Endpoint	Intervention duration	Follow-up duration/assessment
																	occurred	
Peng et al. [46]	2020	34 (33)	34 (33)	68.9 ± 10.2 †§	66.4 ± 11.1 †§	randomized, placebo-controlled study	icotinib, gefitinib, erlotinib, osimertinib	non-small cell lung cancer (NSCLC)	xerosis, pruritus	MASCC, NCI-CTCAE v4.03 (xerosis grading	reactive	oral and topical	Zizao Yangrong Granules, a traditional Chinese medicine formulation (5 herbal), combined with topical vitamin	placebo group; (only placebo granules alongside vitamin E	oral Zizao Yangrong Granules taken twice daily with warm water; vitamin E ointment applied externally	efficacy, QoL	4 weeks	at baseline, at week 2 and at week 4

Study	Year	Patient number of intervention group [ITT (PP)]	Patient number of control group [ITT (PP)]	Age of intervention group [year, median (range)]	Age of control group [year, median (range)]	Type of study	Targeted therapy	Type of malignancy	Type of cutaneous adverse events	Cutaneous adverse events criteria	Type of intervention (preventive or reactive)	Way of administration	Intervention	Control	Dosage	Endpoint	Intervention duration	Follow-up duration/assessment
										g); Soft Plus skin tester (Callegari, Italian) (skin			E ointment	ointment)				

Study	Year	Patient number of intervention group [ITT (PP)]	Patient number of control group [ITT (PP)]	Age of intervention group [year, median (range)]	Age of control group [year, median (range)]	Type of study	Targeted therapy	Type of malignancy	Type of cutaneous adverse events	Cutaneous adverse events criteria	Type of intervention (preventive or reactive)	Way of administration	Intervention	Control	Dosage	Endpoint	Intervention duration	Follow-up duration/assessment
										hydration); DLQI								
Ren et al. [9]	2015	439 (432)	432 (432)	51.8 (20.4–86.4)	52.0 (20.2–84.3)	randomized, controlled, open-label trial	sorafenib	Hepatocellular carcinoma	hand-foot skin reaction, rash, dry skin, erythema	NCI-CTCAE v3.0	preventive	topical	urea-based cream	best supportive care	urea: 10% cream, third times a day on the affected area	QoL, compliance	12 weeks	14 weeks

Study	Year	Patient number of intervention group [ITT (PP)]	Patient number of control group [ITT (PP)]	Age of intervention group [year, median (range)]	Age of control group [year, median (range)]	Type of study	Targeted therapy	Type of malignancy	Type of cutaneous adverse events	Cutaneous adverse events criteria	Type of intervention (preventive or reactive)	Way of administration	Intervention	Control	Dosage	Endpoint	Intervention duration	Follow-up duration/assessment
Rerknimitr et al. [47]	2019	14 (14)	14 (14)	50 ±10.8§, 52 (32-62)		randomized, split-faced controlled trial (TCTR20170811002)	afatinib, erlotinib, gefitinib, lapatinib, cetuximab	breast, colorectal, lung and tongue cancer	acneiform papulopustular eruptions	EI, PGA and papulopustular lesion counts	reactive	topical	pulsed-dye laser at 595 nm wavelength	placebo (sham treatment with cryogen spray)	the laser was delivered covering the entire half face (any topical and/or systemic treatments prescribed were allowed, but not detailed)	efficacy, QoL	8 weeks (every two weeks for four consecutive sessions)	at baseline, week 2, week 4, week 6 for the laser therapy, and at week 8 and 10 as follow-ups

Study	Year	Patient number of intervention group [ITT (PP)]	Patient number of control group [ITT (PP)]	Age of intervention group [year, median (range)]	Age of control group [year, median (range)]	Type of study	Targeted therapy	Type of malignancy	Type of cutaneous adverse events	Cutaneous adverse events criteria	Type of intervention (preventive or reactive)	Way of administration	Intervention	Control	Dosage	Endpoint	Intervention duration	Follow-up duration/assessment
Shinohara et al. [49]	2014	18 (17)	18 (16)	Arm A: 65±10*§ Arm B: 68±7*§	NA	two-arm, open-label, randomized phase II trial (UMIN000002016)	sorafenib	metastatic renal cell carcinoma (RCC)	hand-foot skin reaction (HFSR)	NCI-CTCAE v4.0	reactive (grade 1)	topical	Arm A: Hydrocolloid dressing with ceramide (Remoispad®); Arm B: 10% urea cream (other treatments were prohibited)	no control	Arm A: Hydrocolloid dressing applied every 2-3 days Arm B: Urea cream applied 2-3 times per day	efficacy, QoL, compliance	4 weeks (if the grade of HSFR had not worsened)	biweekly during the 4-week study period

Study	Year	Patient number of intervention group [ITT (PP)]	Patient number of control group [ITT (PP)]	Age of intervention group [year, median (range)]	Age of control group [year, median (range)]	Type of study	Targeted therapy	Type of malignancy	Type of cutaneous adverse events	Cutaneous adverse events criteria	Type of intervention (preventive or reactive)	Way of administration	Intervention	Control	Dosage	Endpoint	Intervention duration	Follow-up duration/assessment
Shinohara et al. [48]	2015	44 (44) (preventive)	52 (52) (reactive)	Preventive: 66 (41-82)	reactive: 67 (34-83)	retrospective, comparative study	erlotinib	advanced pancreatic cancer	acneiform rash, xerosis, and paronychia	NCI-CTCAE v4.0	preventive and reactive (grade 2/3)	oral and topical	minocycline, heparinoid emollient, topical steroid	preventive and reactive were compared	• prophylactic group: minocycline orally 200 mg/day from start of treatment, heparinoid emollienst (strong or medium topical steroids after any grade).<br>• reactive group:	efficacy, compliance	first 6 weeks	during the first 6 weeks, but treatment continued throughout the study period based on patient need and

Study	Year	Patient number of intervention group [ITT (PP)]	Patient number of control group [ITT (PP)]	Age of intervention group [year, median (range)]	Age of control group [year, median (range)]	Type of study	Targeted therapy	Type of malignancy	Type of cutaneous adverse events	Cutaneous adverse events criteria	Type of intervention (preventive or reactive)	Way of administration	Intervention	Control	Dosage	Endpoint	Intervention duration	Follow-up duration/assessment
															heparinoid emollients from start of treatment, strong or medium topical steroids (diflupredn ate 0.05% for the body and hydrocortis one butyrate 0.1% for the face)			progression

Study	Year	Patient number of intervention group [ITT (PP)]	Patient number of control group [ITT (PP)]	Age of intervention group [year, median (range)]	Age of control group [year, median (range)]	Type of study	Targeted therapy	Type of malignancy	Type of cutaneous adverse events	Cutaneous adverse events criteria	Type of intervention (preventive or reactive)	Way of administration	Intervention	Control	Dosage	Endpoint	Intervention duration	Follow-up duration/assessment
															after any grade, minocycline orally 200 mg/day only after grade 2/3 cutaneous adverse events.			
Shou et al. [51]	2021	22 (20)	NA	58 (18-76)*	NA	prospective, non-randomized	sorafenib, anlotinib, apatinib, lenvatinib,	multiple, including hepatocellular carci	hand-foot skin reaction (HFSR)	NCI-CTCAE v5.0, NR	reactive (grade 3)	topical	Shouzu Ning Decoction (SND), an herbal Chinese medicine	no control	200 ml of SND decoction with water to a final volume of 1000 mL, applied as a	efficacy, QoL	until the regression of grade 1, max	baseline and continuously (supposedly daily, it is

Study	Year	Patient number of intervention group [ITT (PP)]	Patient number of control group [ITT (PP)]	Age of intervention group [year, median (range)]	Age of control group [year, median (range)]	Type of study	Targeted therapy	Type of malignancy	Type of cutaneous adverse events	Cutaneous adverse events criteria	Type of intervention (preventive or reactive)	Way of administration	Intervention	Control	Dosage	Endpoint	Intervention duration	Follow-up duration/assessment
						analysis	regorafenib	noma, lung cancer, gastric cancer, colorectal cancer, rectal cancer,		S (pain intensity), HF-QoLS			decoction		hand-and-foot soak for 20 minutes, twice daily		2 weeks	variable)

Study	Year	Patient number of intervention group [ITT (PP)]	Patient number of control group [ITT (PP)]	Age of intervention group [year, median (range)]	Age of control group [year, median (range)]	Type of study	Targeted therapy	Type of malignancy	Type of cutaneous adverse events	Cutaneous adverse events criteria	Type of intervention (preventive or reactive)	Way of administration	Intervention	Control	Dosage	Endpoint	Intervention duration	Follow-up duration/assessment
								esophageal cancer, and renal cancer										
Shou et al. [50]	2023	102 (102)	NA	60±12.18 [†§]	NA	retrospective observational study	apatinib, anlotinib, sorafenib, regorafenib, fruqui	non-small cell lung (NSCLC), colorecta	hand-foot skin reaction (HFSR),	NCI-CTCAE v5.0	reactive (grade 2-3)	oral and topical	three main groups: topical steroids + urea ointment; Shouzu Ning	no control	Topical application of 0.05% halometasone (steroid) with 10% urea ointment, or SND alone or with 10%	efficacy, compliance	variable (1-2 weeks)	not detailed

Study	Year	Patient number of intervention group [ITT (PP)]	Patient number of control group [ITT (PP)]	Age of intervention group [year, median (range)]	Age of control group [year, median (range)]	Type of study	Targeted therapy	Type of malignancy	Type of cutaneous adverse events	Cutaneous adverse events criteria	Type of intervention (preventive or reactive)	Way of administration	Intervention	Control	Dosage	Endpoint	Intervention duration	Follow-up duration/assessment
							ntinib, lenvatinib, cabozantinib, sunitinib, axitinib	l, gastric, pancreatic, small-cell lung (SCLC), renal, esophageal, ovar					Decoction (SND); SND + urea ointment. Simultaneously treated with systemic medications (NSAID, Vitamin E)		urea ointment, applied topically. Simultaneously treated with systemic medications including NSAID (28 patients; 0.2–0.4 mg/day celecoxib), and vitamin E (7 patients; 300 mg/day)			

Study	Year	Patient number of intervention group [ITT (PP)]	Patient number of control group [ITT (PP)]	Age of intervention group [year, median (range)]	Age of control group [year, median (range)]	Type of study	Targeted therapy	Type of malignancy	Type of cutaneous adverse events	Cutaneous adverse events criteria	Type of intervention (preventive or reactive)	Way of administration	Intervention	Control	Dosage	Endpoint	Intervention duration	Follow-up duration/assessment
								ian, cervical, thyroid, small bowel cancer, hepatocellular, nasopharyngeal,										

Study	Year	Patient number of intervention group [ITT (PP)]	Patient number of control group [ITT (PP)]	Age of intervention group [year, median (range)]	Age of control group [year, median (range)]	Type of study	Targeted therapy	Type of malignancy	Type of cutaneous adverse events	Cutaneous adverse events criteria	Type of intervention (preventive or reactive)	Way of administration	Intervention	Control	Dosage	Endpoint	Intervention duration	Follow-up duration/assessment
								cholangio-, ampullary, spinal, thymic carcinoma, sarcoma, melanoma										

Study	Year	Patient number of intervention group [ITT (PP)]	Patient number of control group [ITT (PP)]	Age of intervention group [year, median (range)]	Age of control group [year, median (range)]	Type of study	Targeted therapy	Type of malignancy	Type of cutaneous adverse events	Cutaneous adverse events criteria	Type of intervention (preventive or reactive)	Way of administration	Intervention	Control	Dosage	Endpoint	Intervention duration	Follow-up duration/assessment
								carcinoma of unknown origin										
Tian et al. [52]	2017	73 (67)	37 (33)	55 (range: 31-72) *	53 (range: 33-70) *	randomized, double-blind, placebo-controlle	erlotinib, famitinib, gefitinib, sorafenib, sunitinib,	lung, hepatocellular, renal, intestinal, breast canc	acneiform eruption, paronychia, hand-foot	NCI-CTCAE v3.0	reactive	topical	Compound Danxiong Granules (CDG) containing Chinese 5 herbs	placebo (dextrin-based powder)	topical wash for 20 minutes twice a day for 10 days	efficacy	~1,5 weeks (10 days)	at baseline and end of treatment (10 days post-treatment

Study	Year	Patient number of intervention group [ITT (PP)]	Patient number of control group [ITT (PP)]	Age of intervention group [year, median (range)]	Age of control group [year, median (range)]	Type of study	Targeted therapy	Type of malignancy	Type of cutaneous adverse events	Cutaneous adverse events criteria	Type of intervention (preventive or reactive)	Way of administration	Intervention	Control	Dosage	Endpoint	Intervention duration	Follow-up duration/assessment
						d trial	cetuximab	er and soft tissue sarcoma	skin reaction									initiation)
To hyama et al. [53]	2021	25 (21)	NA	NI	NI	retrospective, observational study	afatinib, erlotinib, gefitinib, osimertinib, cetuximab, panitu	non-small cell lung, colorectal, head and neck	xerotic dermatitis	NCI-CTCAE v5.0	reactive	oral and topical	zinc supplementation (Zinc acetate hydrate or polaprezinc) with topical corticost	no control	zinc supplementation started at 50 mg/day in 18 patients, at 25 mg/day followed by an increase to 50 mg/day after 1 month in two	efficacy	more than 2 months	more than 2 months

Study	Year	Patient number of intervention group [ITT (PP)]	Patient number of control group [ITT (PP)]	Age of intervention group [year, median (range)]	Age of control group [year, median (range)]	Type of study	Targeted therapy	Type of malignancy	Type of cutaneous adverse events	Cutaneous adverse events criteria	Type of intervention (preventive or reactive)	Way of administration	Intervention	Control	Dosage	Endpoint	Intervention duration	Follow-up duration/assessment
							mumab	cancer					eroids and/or emollient		patients, and at 34 mg/day (polaprezinc) in one patient			
Vincenzi et al. [54]	2020	Arm 1: 16 (12); Arm 2: 14 (11)	Arm 3: 14 (12)	arm 1: 30 mg: 69 (43-83)†; arm 2: 10 mg: 73.5 (49-81)†	arm 3: 67.0 (35-76)†	randomized, double-blind, placebo-controlled phas	afatinib, erlotinib, gefitinib, lapatinib, cetuximab, panitumumab	solid tumors (e.g., colorectal, lung, head and neck cancer)	pruritus	NRS, VRS; NCI-CTCAE	reactive	oral	arm 1: orvepitant 30 mg; arm 2: orvepitant 10 mg (NK1 receptor antagonist)	arm 3: placebo (tablet)	10 mg or 30 mg orvepitant orally once a day at bedtime for 4 weeks	efficacy, QoL	4 weeks	at baseline, week 1, week 4, week 8

Study	Year	Patient number of intervention group [ITT (PP)]	Patient number of control group [ITT (PP)]	Age of intervention group [year, median (range)]	Age of control group [year, median (range)]	Type of study	Targeted therapy	Type of malignancy	Type of cutaneous adverse events	Cutaneous adverse events criteria	Type of intervention (preventive or reactive)	Way of administration	Intervention	Control	Dosage	Endpoint	Intervention duration	Follow-up duration/assessment
						e II trial												
Wang et al. [56]	2014	59 (51)	59 (50)	54.3 ±10.9*§	57.7± 9.6*§	randomized, double-blind, placebo-controlled trial	gefitinib	non-small cell lung cancer (NSCLC)	acne-like eruption	NCI-CTCAE v3.0	preventive	topical	Evozac Calming Skin Spray (Evaux Laboratories, France), containing lithium-rich thermal spring water	placebo (physiological saline)	applied topically to the face three times a day (morning, noon, and bedtime)	efficacy	4 weeks	at baseline and week 1, 2, 3, 4

Study	Year	Patient number of intervention group [ITT (PP)]	Patient number of control group [ITT (PP)]	Age of intervention group [year, median (range)]	Age of control group [year, median (range)]	Type of study	Targeted therapy	Type of malignancy	Type of cutaneous adverse events	Cutaneous adverse events criteria	Type of intervention (preventive or reactive)	Way of administration	Intervention	Control	Dosage	Endpoint	Intervention duration	Follow-up duration/assessment
Wang et al. [55]	2020	102 (41)[‡]	NA	56 (range: 27-83)	NA	retrospective analysis	alpelisib, in some cases with anti-HER2/3 inhibitors	breast cancer, including hormone receptor-positive (HR+) and HER2	primarily maculopapular rash, with a smaller occurrence of acneiform rash	CTCAE v4.0	preventive and reactive	oral and topical	Preventive: non-sedating antihistamines (Cetirizine, Loratadine).<br><br>Reactive: oral and topical antihistamines (cetirizine,	no control	Preventive oral antihistamines (43 patients):<br>• cetirizine 10 mg or 5 mg; loratadine 10 mg, hydroxyzine 25 mg daily<br><br>Reactive (grade 1-2) (22 patients):	efficacy	1 or 2 weeks (improvements of symptoms)	throughout the duration of alpelisib therapy (variable, range, 0.5–24.8 months) and for 30 days after treatment discontinuation

Study	Year	Patient number of intervention group [ITT (PP)]	Patient number of control group [ITT (PP)]	Age of intervention group [year, median (range)]	Age of control group [year, median (range)]	Type of study	Targeted therapy	Type of malignancy	Type of cutaneous adverse events	Cutaneous adverse events criteria	Type of intervention (preventive or reactive)	Way of administration	Intervention	Control	Dosage	Endpoint	Intervention duration	Follow-up duration/assessment
								amplified subtypes					loratadine), corticosteroids (prednisone 10 or 20mg 3x/day, clobetasol, triamcinolone, fluocinonide, desoximetasone).		• oral antihistamines combined with topical corticosteroids (45.5%);<br>• oral antihistamines as monotherapy (18.2%)<br><br>Reactive (grade 3) (19 patients):			

Study	Year	Patient number of intervention group [ITT (PP)]	Patient number of control group [ITT (PP)]	Age of intervention group [year, median (range)]	Age of control group [year, median (range)]	Type of study	Targeted therapy	Type of malignancy	Type of cutaneous adverse events	Cutaneous adverse events criteria	Type of intervention (preventive or reactive)	Way of administration	Intervention	Control	Dosage	Endpoint	Intervention duration	Follow-up duration/assessment
															• oral antihistamines, topical and systemic steroids (63.2%);<br>• oral antihistamines with topical corticosteroids (15.8%);<br>• systemic steroids as monotherapy (15.8%)			

**Study**	**Year**	**Patient number of intervention group [ITT (PP)]**	**Patient number of control group [ITT (PP)]**	**Age of intervention group [year, median (range)]**	**Age of control group [year, median (range)]**	**Type of study**	**Targeted therapy**	**Type of malignancy**	**Type of cutaneous adverse events**	**Cutaneous adverse events criteria**	**Type of intervention (preventive or reactive)**	**Way of administration**	**Intervention**	**Control**	**Dosage**	**Endpoint**	**Intervention duration**	**Follow-up duration/assessment**
Wong et al. [57]	2010	20 (13)	NA	51.2 (32-71)*	NA	prospective, observational, crossover study (right-left face comparing	erlotinib, cetuximab, panitumumab	non-small cell lung, colorectal, head and neck cancers	papulopustular rash (acneiform eruption), primarily on face	NCI-CTCAE v3.0	reactive (grade ≥2)	topical	Regenecare Wound Gel containing 2% lidocaine, collagen, vitamin E, and aloe vera	left side of face served as untreated control for the first week; then	applied topically to the right side of the face 3-4 times a day for one week; after one week, applied to both sides of the face for up to an additional 5 weeks	efficacy, compliance	6 weeks	weekly evaluations for up to 6 weeks

Study	Year	Patient number of intervention group [ITT (PP)]	Patient number of control group [ITT (PP)]	Age of intervention group [year, median (range)]	Age of control group [year, median (range)]	Type of study	Targeted therapy	Type of malignancy	Type of cutaneous adverse events	Cutaneous adverse events criteria	Type of intervention (preventive or reactive)	Way of administration	Intervention	Control	Dosage	Endpoint	Intervention duration	Follow-up duration/assessment
						study)								treated with gel after crossover				
Wu et al. [58]	2021	135 (107, with adverse events)	NA	62 (21-85) †	NA	phase I dose-escalation and expansio	ulixertinib	advanced solid tumors with BRAF and NR	multiple dermatologic adverse events (dA	CTCAE v4.03	reactive	oral and topical	treatments varied by cutaneous adverse events type: • oral steroid, antibiot	no endpoint	maculopapular rash: • oral prednisone (0.5mg/kg/day for 7–10 days); topical steroids.	efficacy	variable	not exact, adverse events were monitored continuously,

Study	Year	Patient number of intervention group [ITT (PP)]	Patient number of control group [ITT (PP)]	Age of intervention group [year, median (range)]	Age of control group [year, median (range)]	Type of study	Targeted therapy	Type of malignancy	Type of cutaneous adverse events	Cutaneous adverse events criteria	Type of intervention (preventive or reactive)	Way of administration	Intervention	Control	Dosage	Endpoint	Intervention duration	Follow-up duration/assessment
						n trial		AS mutations	Es): acneiform rash, maculopapular rash, rash unspecified, pruritus, dry skin,				ic, pregabalin, antihistamine<br>topical steroid, antibiotic, sunscreen		acneiform rash:<br>• topical antibiotics (clindamycin gel or dapsone gel) two times a day;<br>• oral antibiotics (doxycycline or minocycline) 100 mg twice a day;<br><br>pruritus:			

Study	Year	Patient number of intervention group [ITT (PP)]	Patient number of control group [ITT (PP)]	Age of intervention group [year, median (range)]	Age of control group [year, median (range)]	Type of study	Targeted therapy	Type of malignancy	Type of cutaneous adverse events	Cutaneous adverse events criteria	Type of intervention (preventive or reactive)	Way of administration	Intervention	Control	Dosage	Endpoint	Intervention duration	Follow-up duration/assessment
									photosensitivity, alopecia, erythema multiforme						• topical steroids two times a day in combination with oral antihistamine 4 times a day for pruritus;<br>• short-term oral steroids (prednisone 40mg daily) and concurrent pregabalin			

Study	Year	Patient number of intervention group [ITT (PP)]	Patient number of control group [ITT (PP)]	Age of intervention group [year, median (range)]	Age of control group [year, median (range)]	Type of study	Targeted therapy	Type of malignancy	Type of cutaneous adverse events	Cutaneous adverse events criteria	Type of intervention (preventive or reactive)	Way of administration	Intervention	Control	Dosage	Endpoint	Intervention duration	Follow-up duration/assessment
															50–75mg twice-three times a day for 5 and 10 days<br><br>photosensitivity:<br>topical or oral short-term steroids (with grade 3) and sun protection			
Yamamoto	2022	24 (17)	NA	61 (30-74) †*	NA	single-center,	sunitinib	metastatic renal	hand-foot skin react	NCI-CTCA	preventive	topical	1% bis-glyceryl ascorbate - cream	no control	applied to affected areas three times daily, in	efficacy	6 weeks	during the 6-week interven

Study	Year	Patient number of intervention group [ITT (PP)]	Patient number of control group [ITT (PP)]	Age of intervention group [year, median (range)]	Age of control group [year, median (range)]	Type of study	Targeted therapy	Type of malignancy	Type of cutaneous adverse events	Cutaneous adverse events criteria	Type of intervention (preventive or reactive)	Way of administration	Intervention	Control	Dosage	Endpoint	Intervention duration	Follow-up duration/assessment
et al. [59]						open-label, single-arm, Phase I/II clinical trial		cell carcinoma (mRCC)	ion (HFSR)	E v4.0			(DGA cream), heparinoid, urea cream		conjunction with heparinoid or urea-containing moisturizer			tion period and followed for progression-free survival over longer periods
Yen et al. [60]	2020	35 (35)	NA	62±10.1[§] (range: 35-83)[*]	NA	observational study	afatinib, erlotinib, gefitinib	non-small cell lung cancer	severe paronychia with	NCI-CTCAE	reactive	topical	betaxolol 0.25% ophthalmic solution,	no control	topical application of betaxolol 0.25% solution, applied once	efficacy, compliance	treatment continued for up to	weekly; pain reduction and lesion severity

Study	Year	Patient number of intervention group [ITT (PP)]	Patient number of control group [ITT (PP)]	Age of intervention group [year, median (range)]	Age of control group [year, median (range)]	Type of study	Targeted therapy	Type of malignancy	Type of cutaneous adverse events	Cutaneous adverse events criteria	Type of intervention (preventive or reactive)	Way of administration	Intervention	Control	Dosage	Endpoint	Intervention duration	Follow-up duration/assessment
								(NSCLC)	pyogenic granuloma-like lesions	v4.0			applied topically		daily to affected areas (paronychia with pyogenic granuloma-like lesions)		12 weeks or until complete resolution	were assessed after 1 week, 4 weeks, and 12 weeks, with photographic evidence at each follow-up
Zhang	20	29 (27)	29 (27)	58 ± 14.29[*§]	53 ± 13.	double-blin	afatinib, daco	lung, colorecta	papulopustula	MASCC	reactive	oral and	topical Zhiyang Pingfu	placebo (solu	liquids were applied twice a day for 30	efficacy	2 weeks	at baseline, 7 days,

Study	Year	Patient number of intervention group [ITT (PP)]	Patient number of control group [ITT (PP)]	Age of intervention group [year, median (range)]	Age of control group [year, median (range)]	Type of study	Targeted therapy	Type of malignancy	Type of cutaneous adverse events	Cutaneous adverse events criteria	Type of intervention (preventive or reactive)	Way of administration	Intervention	Control	Dosage	Endpoint	Intervention duration	Follow-up duration/assessment
et al. [61]	22				46*§	d, randomized, placebo-controlled clinical study	mitinib, erlotinib, gefitinib, icotinib, poziotinib, cetuximab	l cancer	r rash, xerosis, paronychia			topical	Liquid containing 5 traditional Chinese medicinal herbs	tion with similar appearance)	minutes, after that with half hour interval 0.1 % hydrocortison butyrate cream; 100 mg minocycline and, if needed, 20 mg methylprednisolone orally were administered for 14 days			and 14 days after treatment initiation

Best supportive care (BSC) refers to the optimal supportive treatment provided to alleviate symptoms and improve the quality of life of the patient without directly treating the underlying disease; **compliance**: compliance to the oncological therapy; **"historical control"** refers to a control group with patients who were treated under similar conditions in the past and were not randomized in the current study; **HFSR:** hand-foot skin reaction; **NCI-CTCAE**: National Cancer Institute - Common Terminology Criteria for Adverse Events; **QoL:** Quality of life; **reactive treatment** is an approach where treatment is initiated only after symptoms or side effects appear, rather than as a preventive measure;

† : data of ITT (intention-to-treat) group

*: data of PP (per-protocol) group

§: mean

‡: patients, who recieved any kind of treatment for cutaneous adverse events caused by alpelisib

Table S2. Summary of the results of studies included only in the systematic review [2,4,5,10-30,32,33,35-41,43-61]

Study	Year	Patient number of intervention group [ITT(PP)]	Patient number of control group [ITT (PP)]	Type of cutaneous adverse events	Type of intervention (preventive/reactive)	Way of administration	Intervention	Control	Endpoints	Value of endpoints
**Studies with preventive interventions**										
Amitay-Laish et al. [11]	2020	arm 1: 31 (21, day 14; 17, day 30); arm 2: 30 (23, day 14; 19, day 30)	arm 3: 30 (25, day 14; 21, day 30)	papulopustular rash	preventive	topical	chloramphenicol 3% + prednisolone 0.5% (arm 1: CHL-PRED) and chloramphenicol 3% (arm 2: CHL) (and sunscreen)	placebo (aqua cream) (arm 3: AQUA)	• efficacy	• incidence of grade ≥3 rash (CTCAE):<br>○ Day 14: CHL-PRED: 0%; CHL: 5% (1/23); AQUA: 20% (5/25)<br>○ Day 30: CHL-PRED: 0%; CHL: 5% (1/19); AQUA: 5% (1/21)<br>• incidence of ≥10 facial papulopustular lesions (NOL):<br>○ Day 14: CHL-PRED: 14% (3/21); CHL: 39% 9/23); AQUA: 48% 12/25)<br>○ Day 30: CHL-PRED: 6% (1/17); CHL: 16% (3/19); AQUA: 43% (9/21)

Study	Year	Patient number of intervention group [ITT(PP)]	Patient number of control group [ITT(PP)]	Type of cutaneous adverse events	Type of intervention (preventive/reactive)	Way of administration	Intervention	Control	Endpoints	Value of endpoints
Chayahara et al. [15]	2019	36 (26)	NA	acneiform rash	preventive	oral and topical	adapalene gel 0.1% (additional minocycline, and moisturizer)	placebo (other half of face)	• efficacy	• the difference in total facial lesion count of rash at 4 weeks (mean): intervention: 12.6, control: 9.8 ($p = 0.12$)<br>• complete control rate of rash (≤5 facial lesions): intervention: 54%, control: 50%<br>• global skin assessment at 4 weeks (mean IGA grade): intervention: 1.9, control: 1.7
Chen J. C. et al. [2]	2020	58 (58)	58 (58)	hand-foot skin reaction, rash, alopecia	preventive	oral	celecoxib	no treatment	• adherence	• dose modification/interruption rate ($p < 0.001$):<br>○ intervention: 15.5% (9/58)<br>○ control: 46.6% (27/58)
Deplanque et al. [4]	2016	73 (21, month 4; 7, month 12)	74 (25, month 4; 11, month 12)	folliculitis, skin lesions, xerosis, paronychia	preventive	oral	doxycycline	placebo	• QoL<br>• adherence	• percentage of patients with DLQI score 0-1 (no effect of skin alterations on QoL):<br>○ at day 14: intervention: 70%, control: 42% ($p = 0.02$)<br>○ at day 28: intervention: 67%, control: 41% ($p = 0.06$)<br>• modification/discontinuation of oncological therapy:

Study	Year	Patient number of intervention group [ITT(PP)]	Patient number of control group [ITT(PP)]	Type of cutaneous adverse events	Type of intervention (preventive/reactive)	Way of administration	Intervention	Control	Endpoints	Value of endpoints
										○ after 4 month: intervention group: 25%, control group: 37%<br>○ whole treatment period: intervention group: 25%, control group: 43%
Fuggetta et al. [18]	2019	34 (34)	NA	rash (interfollicular and follicular-based erythematous papules and pustules)	preventive	topical	1.5% polydatin-based cream (GHIMAS) (with additional SPF 30 UVA/UVB non occlusive sunscreen)	no control (comparison with LUX-Lung 3/6 study)	• efficacy<br>• adherence	• incidence of rash:<br>○ any grade: 41.2% (14/34)<br>○ grade 1: 20.6% (7/34)<br>○ grade 2: 20.6% (7/34)<br>○ grade 3: 0%<br>• discontinuation rate of oncological therapy: 0%
Grande et al. [21]	2013	51 (51)	NA	papulopustular rash, xerosis,	preventive	oral and topical	lymecycline, skin moisturizers and sunscreen	no control	• efficacy<br>• QoL	• incidence of grade ≥2 cutaneous adverse events during the first 3 months of therapy: 27.4% (14/51): 71.4% in the first month, 21.4% in the second month, and 7.2% in the third month<br>• incidence of grade ≥3 events: 0%

Study	Year	Patient number of intervention group [ITT(PP)]	Patient number of control group [ITT(PP)]	Type of cutaneous adverse events	Type of intervention (preventive/reactive)	Way of administration	Intervention	Control	Endpoints	Value of endpoints
				paronychia						• QoL assessment: 0–1: 66.7% (34/51); 2–5: 23.5% (12/51); 6–10: 9.8% (5/51); ≥11: 0% (0/0)
Ichiki et al. [23]	2017	29 (29)	NA	oral mucositis, skin rash, paronychia	preventive	oral and topical	Kampo medicine (TJ-14), minocycline	no control (comparison with LUX-Lung 3/6 study)	• efficacy	• incidence of side effects during the first 4 weeks of treatment:<br>○ any grade oral mucositis: 68.9% (20/29)<br>▪ grade 1: (9/29)<br>▪ grade 2: (10/29)<br>▪ grade 3: 3.4% (1/29)<br>○ any grade skin rash: 44.8% (13/29)<br>▪ grade 1: 41.4% (12/29)<br>▪ grade 2: 3.4% (1/29)<br>○ any grade paronychia: 17.2% (5/29)<br>▪ grade 1: 10.3% (3/29)<br>▪ grade 2: 6.9% (2/29)
Iimura et al. [24]	2018	49 (20); group 1: 12, group 2: 5,	NA	rash (the face and upper body)	preventive	oral and topical	non-steroidal anti-inflammatory drugs (group 1), specificall	no control	• efficacy	• the effect of NSAID/steroid/ acetylsalicylic acid coadministration on rash incidence by Cox proportional hazards model:<br>• NSAID: adjusted hazard ratio [95%CI]: HR = 0.149 [0.180 – 0.976], p = 0.044 (indicating a significant reduction of the risk of developing rash)

Study	Year	Patient number of intervention group [ITT(PP)]	Patient number of control group [ITT (PP)]	Type of cutaneous adverse events	Type of intervention (preventive/reactive)	Way of administration	Intervention	Control	Endpoints	Value of endpoints
		group 3: 3					y loxoprofen; steroid (group 2); acetylsalicylic acid (group 3) Additional basic skin care with sunscreen, moisturizer, and topical 1% hydrocortisone.			• steroid: adjusted hazard ratio [95%CI]: HR = 0.172 [0.023 – 1.311], p = 0.089 (indicating a trend of reduction of the risk of developing rash)<br>• acetylsalicylic acid: adjusted hazard ratio [95%CI]: HR = 2.042 [0.570 – 7.318], p = 0.273 (indicating no significant association with the reduction of skin rash)
Ishikawa et al. [25]	2017	60 (55)	NA	hand-foot skin reaction (HFSR)	preventive	topical	topical steroids (0.05% difluprednate) and	no control, historical compar	• efficacy<br>• adherence	• incidence rate by severity: any grade: 73% (40/55); grade 3: 22% (12/55)<br>• time of onset of grade ≥2: 42% (23/55) in the first cycle, 11% (6/55) in the later cycle;

Study	Year	Patient number of intervention group [ITT(PP)]	Patient number of control group [ITT(PP)]	Type of cutaneous adverse events	Type of intervention (preventive/reactive)	Way of administration	Intervention	Control	Endpoints	Value of endpoints
							20% urea-based cream	ison with CORRECT study (Japanese subgroup)		• delaying of oncological therapy due to HSFR: 33% (18/55)<br>• reducing of oncological therapy due to HSFR: 61% (34/55)<br>• discontinuation of oncological therapy due to HSFR: 0%
Iwasaku et al. [26,27]	2019; 2023	41 (41)	NA	acneiform rash, dry skin, pruritus, paronychia, maculopapular rash, erythema multiforme, HSFR,	preventive	oral and topical	minocycline, heparinoid moisturizer, medium and very strong topical steroids, sunscreen	no control group; historical comparison with ARCHER1050 and ARCH	• efficacy<br>• adherence	• incidence rate of any grade during 8 weeks:<br>○ acneiform rash: grade 1: 41.5% (17/41), grade 2: 24.4% (10/41), grade 3: 2.4% (1/41)<br>○ paronychia: grade 1: 63.4% (26/41), grade 2: 9.8% (4/41), grade 3: 2.4% (1/41)<br>○ pruritus: grade 1: 26.8% (11/41), grade 2: 7.3% (3/41), grade 3: 0%<br>○ rash maculopapular: grade 1: 26.8% (11/41), grade 2: 7.3% (3/41), grade 3: 0%<br>○ erythema multiforme: grade 1: 7.3% (3/41), grade 2: 2.4% (1/41), grade 3: 4.9% (2/41)

Study	Year	Patient number of intervention group [ITT(PP)]	Patient number of control group [ITT(PP)]	Type of cutaneous adverse events	Type of intervention (preventive/reactive)	Way of administration	Intervention	Control	Endpoints	Value of endpoints
				skin infection, eczema				ER1042 trials		o dry skin: grade 1: 63.4% (26/41), grade 2: 4.9% (2/41), grade 3: 0%<br>o HSFR: grade 1: 7.3% (3/41), grade 2: 2.4% (1/41), grade 3: 0%<br>o skin infection: grade 1: 2.4% (1/41), grade 2: 2.4% (1/41), grade 3: 0%<br>o eczema: grade 1: 4.9% (2/41), grade 2: 0%, grade 3: 0%<br>• dose reduction rate of oncological therapy during 8 weeks: 19.5% (8/41);<br>• discontinuation of oncological therapy during 8 weeks: 22.2% (9/41)<br>• interruption of oncological therapy during 8 weeks: 51.2% (21/41)
Jatoi et al. [29]	2008	31 (30,15)	30 (29, 12)	papulopustular rash	preventive	oral	tetracycline	placebo	• efficacy	• any grade rash incidence reported by physician (week 4) (p = 0.61)<br>o intervention group: 70% (16/23)<br>o control group: 76% (22/29)<br>• grade 2 or >50% surface area rash incidence reported by physician (week 4) (p = 0.009)<br>o intervention group: 17% (4/23)

Study	Year	Patient number of intervention group [ITT(PP)]	Patient number of control group [ITT (PP)]	Type of cutaneous adverse events	Type of intervention (preventive/reactive)	Way of administration	Intervention	Control	Endpoints	Value of endpoints
										o control group: 55% (16/29)<br>• any grade rash incidence reported by physician (week 8) (p = 0.84)<br>o intervention group: 87% (13/15)<br>o control group: 84% (16/19)<br>• grade 2 or >50% surface area rash incidence reported by physician (week 8) (p = 0.3)<br>o intervention group: 27% (4/15)<br>o control group: 47% (9/19)<br>• rash coverage with 51–75% of body surface (week 5-8) (p = 0.04)<br>o intervention group: 0%<br>o control group: 19% (3/16)
Lacouture et al. [5]	2016	cohort I: 66 (56); cohort II: 72 (59)	66 (58)	rash, dry skin, dermatitis acneiform, paronych	preventive	oral and topical	doxycycline (cohort I) or alclometasone (cohort II)	placebo	• QoL	• incidence of adverse events in the first 8 weeks of treatment for cohort II:<br>o all grade, n(%): 47 (79.7)<br>o grade ≥2, n (%): 21 (35.6)<br>• QoL assessment (SKINDEX-16), worsening rate (visits):

Study	Year	Patient number of intervention group [ITT(PP)]	Patient number of control group [ITT (PP)]	Type of cutaneous adverse events	Type of intervention (preventive/reactive)	Way of administration	Intervention	Control	Endpoints	Value of endpoints
				ia, rash maculopapular, rash pustular, fissure						○ Cohort I – Placebo: Symptoms: 6/7, Emotions: 6/7, Functioning: 2/7<br>○ Cohort I – Doxycycline: Symptoms: 1/7, Emotions: 1/7, Functioning: 0/7<br>○ Cohort II – Alclometasone: Symptoms: 6/7, Emotions: 5/7, Functioning: 0/7
Li et al. [34]	2022	14 (7)	16 (14)	skin toxicity, paronychia, acneiform rash, rash maculopapular	preventive	oral	traditional Chinese medicine	placebo	• efficacy<br>• QoL	• skin toxicity on day 7, n (%), PP population:<br>○ intervention group: 2 (28.6%)<br>○ control group: 2 (14.3%)<br>• skin toxicity on day 14, n (%), PP population:<br>○ intervention group: 3 (14.3%)<br>○ control group: 8 (57.1%)<br>• changes in ECOG PS scores (before study – after study), PP population:<br>○ intervention group: no changes<br>○ control group: -1.472 (p = 0.165)<br>• changes in FACT-L (before study – after study), PP population:

Study	Year	Patient number of intervention group [ITT(PP)]	Patient number of control group [ITT(PP)]	Type of cutaneous adverse events	Type of intervention (preventive/reactive)	Way of administration	Intervention	Control	Endpoints	Value of endpoints
										○ intervention group: PWB: 1.099 (p = 0.314); SWB: 0.510 (P = 0.629); EWB: 0.141 (p= 893); FWB: -1.310 (p = 0.238); LCS: 0.126 (p = 0.904)<br>○ control group: PWB: -0.665 (p = 0.517); SWB: 2.233 (p = 0.044); EWB: −0.832 (p = 0.421); FWB: 0.384 (p = 0.707); LCS: −1.104 (p = 0.290)<br>• changes in EORTC QLQ-C30, quality of life (before study – after study), PP population:<br>○ intervention group: -1.681 (p = 0.144)<br>○ control group: 1.383 (p = 0.190)<br>• changes in EORTC QLQ-L13, total score (before study – after study), PP population:<br>○ intervention group: 0.664 (p = 0.531)<br>○ control group: 0.0000 (p = 1.0000)
Lu et al. [38]	2021	40 (40)	91 (91)	paronychia	preventive	topical	betaxolol 0.25% solution	no treatment	• efficacy	• cumulative incidence of paronychia over 3 months (p < 0.01):<br>○ intervention group: 20%<br>○ control group: 58.2,

Study	Year	Patient number of intervention group [ITT(PP)]	Patient number of control group [ITT (PP)]	Type of cutaneous adverse events	Type of intervention (preventive/reactive)	Way of administration	Intervention	Control	Endpoints	Value of endpoints
										• number of involved digits (fingernails or toenails) with lesions (p = 0.07):<br>o intervention group: 2.25 digits (range: 1–5)<br>o control group: 3.03 digits (range: 1–7)<br>• paronychia severity (CTCAE grade), mean (p < 0.01):<br>o intervention group: 1.5 (no grade 3 occured)<br>o control group: 2.12<br>• pain severity (VAS score), mean (p < 0.01):<br>o intervention group: 3.125<br>o control group: 6.29
Masago et al. [39]	2018	51 (preintervention cohort: 32; postintervention	NA	acneiform rash, dry skin, paronychia, and pruritus	preventive	oral and topical	multimodal team management – pre-intervention	multimodal team management – post-intervention	• efficacy<br>• QoL	• incidence of cutaneous adverse events in pre-intervention:<br>o acneiform rash: grade 1: 59.3% (19/32); grade 2: 12.5% (4/32); grade 3: 3.1% (1/32)<br>o dry skin: grade 1: 31.3% (10/32); grade 2: 46.9% (15/32); grade 3: 9.4% (3/32);<br>o paronychia: grade 1: 15.6% (5/32); grade 2: 9.4% (3/32); grade 3: 12.5% (4/32);<br>o pruritus: grade 1: 50% (16/32), grade 2: 9.4% (3/32), grade 3: 3.1% (1/32);

Study	Year	Patient number of intervention group [ITT(PP)]	Patient number of control group [ITT (PP)]	Type of cutaneous adverse events	Type of intervention (preventive/reactive)	Way of administration	Intervention	Control	Endpoints	Value of endpoints
		cohort : 27)								• incidence of cutaneous adverse events in post-intervention:<br>o acneiform rash: grade 1: 48.1% (13/27), grade 2: 18.5% (5/27), grade 3: 0% (0/27)<br>o dry skin: grade 1: 40.7% (11/27), grade 2: 33.3% (9/27), grade 3: 0% (0/27);<br>o paronychia: grade 1: 18.5% (5/27), grade 2: 7.4% (2/27), grade 3: 0% (0/27);<br>o pruritus: grade 1: 33.3% (9/27), grade 2: 0%, grade 3: 0% (0/27);<br>• mean (range) DLQI (0-30) of all patients:<br>o pre-intervention: 3.281 (0-19)<br>o post-intervention: 3.556 (0-13)<br>• mean SKINDEX-29 (0-100) of all patients:<br>o pre-intervention: 16.380<br>o post-intervention: 15.517
Melosky et al. [41]	2019	10	NA	hand-foot syndrome	preventive	oral	perindopril	no control (only	• efficacy	• incidence of grade 1-2: 20% (2/10)<br>• incidence of grade 3: 50% (5/10) (worse than historical data)

Study	Year	Patient number of intervention group [ITT(PP)]	Patient number of control group [ITT (PP)]	Type of cutaneous adverse events	Type of intervention (preventive/reactive)	Way of administration	Intervention	Control	Endpoints	Value of endpoints
								historical data)		
Nishizawa et al. [44]	2023	28 (27)	NA	hand-foot skin reaction (HFSR)	preventive	topical	aluminum chloride ointment (30%)	no control	• efficacy, adherence	•incidence of grade 3: 7.4% (2/27)<br>•incidence of all grades: 66.7% (18/27); Grade 1: 22.2%, Grade 2: 37.0%, Grade 3: 7.4%<br>•time to development of HFSR of any grade, median days: median 15 days<br>•treatment discontinuation or dosage reduction rate due to HSFR: 0%<br>•treatment interruption rate due to HSFR: 14% (3/22)
Okajima et al. [45]	2021	46 (35)	NA	rash/acne, paronychia, and oral mucositis	preventive	oral and topical	minocycline, topical medium-class steroid or sodium azulene gargle	no control	• efficacy	• prophylaxis rate of grade ≥3 side effects after 4 weeks: mucositis: 95.6%, paronychia: 100%, rash/acne: 97.8%

Study	Year	Patient number of intervention group [ITT(PP)]	Patient number of control group [ITT (PP)]	Type of cutaneous adverse events	Type of intervention (preventive/reactive)	Way of administration	Intervention	Control	Endpoints	Value of endpoints
Ren et al. [9]	2015	439 (432)	432 (432)	hand-foot skin reaction, rash, dry skin, erythema	preventive	topical	urea-based cream	best supportive care	• QoL<br>• adherence	• mean HFSR symptom score:<br>o intervention group: week 2: 4.8, week 4: 9.3, week 6: 9, week 8: 8.3, week 10: 7.5, week 12: 6.4, week 14: 4.9<br>o control group: week 2: 5.2, week 4: 11.5, week 6: 12.4, week 8: 11.1, week 10: 8.9, week 12: 7.1, week 14: 5.1<br>• mean HFSR daily activity score:<br>o intervention group: week 2: 3.8, week 4: 7.2, week 6: 6.9, week 8: 6.4, week 10: 6.0, week 12: 5.3, week 14: 4.9<br>o control group: week 2: 4.0, week 4: 8.3, week 6: 9.6, week 8: 7.6, week 10: 6.2, week 12: 4.9, week 14: 3.9<br>• dose reductions/interruptions as a result of HFSR ($p = 0.1937$):<br>o intervention group: 9.1%<br>o control group: 11.8%<br>• dose discontinuations as a result of HFSR ($p = 0.4893$):<br>o intervention group: 1.1%

Study	Year	Patient number of intervention group [ITT(PP)]	Patient number of control group [ITT (PP)]	Type of cutaneous adverse events	Type of intervention (preventive /reactive)	Way of administration	Intervention	Control	Endpoints	Value of endpoints
										o control group: 0.7%
Wang et al. [56]	2014	59 (51)	59 (50)	acne-like eruption	preventive	topical	Evozac Calming Skin Spray (Evaux Laboratories, France), containing lithium-rich thermal spring water	placebo (physiological saline)	• efficacy	• difference in the total number of face lesions at Week 4 (mean, range) (p = 0.058):<br>o intervention group: 6.18 (0-63)<br>o control group: 8.02 (0-59)<br>• difference in total number of face lesions in weekly evaluation (mean, range) (Week 1: p = 0.031, Week 2: p=0.088, Week 3: p = 0.022):<br>o intervention group: Week 1: 0.25 (0-4); Week 2: 2.96 (0-21); Week 3: 6.67 (0-59)<br>o control group: Week 1: 1.10 (0-12); Week 2: 5.34 (0-38); Week 3: 12.26 (0-80)<br>• incidence of grade ≥2 rash at Week 4 (p = 0.037):<br>o intervention group: 17.6% (9/51)<br>o control group: 36.0% (18/50)<br>• incidence of rash-associated symptoms at Week 4 (p = 0.017):<br>o intervention group: 13.7% (7/51)<br>o control group: 34.0% (17/50)
Yamamoto	2022	24 (17)	NA	hand-foot skin	preventive	topical	1% bis-glyceryl	no control	• efficacy	• incidence of HSFR within 6 weeks (2 cycles of sunitinib therapy): 11.8% (2/17)

Study	Year	Patient number of intervention group [ITT(PP)]	Patient number of control group [ITT(PP)]	Type of cutaneous adverse events	Type of intervention (preventive/reactive)	Way of administration	Intervention	Control	Endpoints	Value of endpoints
et al. [59]				reaction (HFSR)			ascorbate-cream (DGA cream), heparinoid, urea cream			•development of ≥ grade 1 within 6 weeks (2 cycles of sunitinib therapy): 1.8% (grade 1: 5.9%, grade 2: 5.9%)<br>•development of ≥ grade 2 HFSR within 6 weeks: 5.9% (1/17)<br>•development of ≥ grade 2 HFSR within 3 weeks after the investigational treatment: 5.9% (1/17)
**Studies with preventive and reactive interventions**										
Bavetta et al. [12]	2021	Group A: 20 Group B: 20	NA	papulopustular rash	preventive (group B) and reactive (group A)	oral and topical	polydatin cream, minocycline	no control	• efficacy,<br>• QoL	•efficacy (improvement): 100% (20/20) patients improved with 1.5% polydatin by week 4<br>•mean CTCAE:<br>o group A: week 0: 1.85, week 4: 1.15, week 8: 0.70, week 12: 0.55, week 24: 0.37<br>o group B: week 0: 0, week 4: 0.05, week 8: 0, week 12: 0, week 24: 0<br>•preventive effect in symptom-free patients: 0/20 patients developed rash during 12-week prophylaxis; 1 mild erythema (resolved)<br>•mean DLQI:<br>o group A: week 0: 8.1, week 4: 5.2, week 8: 3.25, week 12: 3.2, week 24: 2.00

Study	Year	Patient number of intervention group [ITT(PP)]	Patient number of control group [ITT (PP)]	Type of cutaneous adverse events	Type of intervention (preventive/reactive)	Way of administration	Intervention	Control	Endpoints	Value of endpoints
										○ group B: week 0: 3.0, week 4: 2.28, week 8: 1.06, week 12: 0.44, week 24: 0.44
Goto et al. [20]	2018	35 (25); group 1: 6. group 2: 19	NA	rash, primarily occurring within a median of 7 days after afatinib initiation, paronychia	preventive and reactive (grade ≥1)	oral and topical	prevention (grade 0) (group 1): minocycline (additional moisturizing agents, sunblock);<br><br>reactive (grade ≥1) (group 2): minocycline, topical corticosteroids	no control	• efficacy<br>• adherence	• incidence of any grade rash: 92% (23/25)<br>• incidence of grade 1 rash in group 1: 4/6<br>• incidence of grade 2 rash in group 2: 3/19<br>• dose reduction rate of oncological therapy because of rash: 0%<br>• dose reduction rate of oncological therapy because of paronychia: 12% (3/25)
Jatoi et al. [28]	2021	61 (61)	55 (55)	hand-foot skin	preventive and	topical	0.05% clobetasol cream,	0.05% clobetasol	• efficacy	• percentage of patients with no HFSR during the first two regorafenib cycles: preventive: 30% (18/61); reactive: 13% (7/55)

Study	Year	Patient number of intervention group [ITT(PP)]	Patient number of control group [ITT(PP)]	Type of cutaneous adverse events	Type of intervention (preventive/reactive)	Way of administration	Intervention	Control	Endpoints	Value of endpoints
		(preventive)	(reactive)	reaction (HFSR)	reactive		applied preemptively (before HFSR development)	cream, applied reactively (after HFSR onset)	• QoL	• percentage of patients during the first regorafenib cycles:<br>o with no HFSR: preventive: 54% (33/61); reactive: 45% (25/55)<br>o with any HSFR: preventive: 46% (28/61) (grade 1: 18% (11/61), grade 2: 18% (11/61), grade 3: 10% (6/61)); reactive: 55% (30/55) (grade 1: 15% (8/55), grade 2: 24% (13/55), grade 3: 11% (6/55))<br>• percentage of patients during the second regorafenib cycles:<br>o with no HFSR: preventive: 33% (20/61); reactive: 15% (8/55)<br>o with any HSFR: preventive: 67% (41/61) (grade 1: 30% (18/61), grade 2: 8% (5/61), grade 3: 3% (2/61)); reactive: 85% (47/55) (grade 1: 43% (18/55), grade 2: 18% (10/55), grade 3: 7% (4/55))

Study	Year	Patient number of intervention group [ITT(PP)]	Patient number of control group [ITT (PP)]	Type of cutaneous adverse events	Type of intervention (preventive/reactive)	Way of administration	Intervention	Control	Endpoints	Value of endpoints
										• quality of life: cycle 2, day 28: mean HFS score was 46 (preventive) vs. 67 (reactive)
Liu Z. et al. [37]	2022	arm A: 46*<br>arm B: 47*	arm C: 46*	acneiform rash, pruritus	preventive and reactive	oral and topical	preventive (arm A): honeysuckle.<br>reactive 1 (arm B): honeysuckle.	reactive 2 (arm C - conventional treatment): minocycline, clindamycin and hydrocortisone	• efficacy	• incidence of any grade rash (p = 0.280 – not significant):<br>o arm A (preventive honeysuckle): 56.5% (26/46)<br>o arm B (reactive honeysuckle): 68.1% (32/47)<br>o arm C (conventional, reactive treatment): 71.7% (33/46)<br>• incidence of grade 1-2-3 rash (p = 0.027 - statistically significant difference in favor of Arm A):<br>o arm A (preventive honeysuckle): grade 1: 32.6% (15/46), grade 2: 17.4% (8/46), grade 3: 4.3% (2/46)<br>o arm B (reactive honeysuckle): grade 1: 36.2% (15/47), grade 2: 34.0% (16/47), grade 3: 4.3% (2/47)<br>o arm C (conventional, reactive treatment): grade 1: 26.1% (12/46), grade 2: 30.4% (14/46), grade 3: 15.2% (7/46)

Study	Year	Patient number of intervention group [ITT(PP)]	Patient number of control group [ITT(PP)]	Type of cutaneous adverse events	Type of intervention (preventive/reactive)	Way of administration	Intervention	Control	Endpoints	Value of endpoints
										• the duration from onset of grade ≥2 rashes relieved to grade 1 or 0: Arm A recovered faster than B and C (explicit data no mentioned)<br>• complete recovery rates:<br>o arm A: 34.8% (8/23 patients)<br>o arm B: 21.7% (5/23 patients)<br>o arm C: 17.4% (4/23 patients)<br>• time to recovery from pruritus (p = 0.016 → significantly faster recovery with Arm A)<br>o arm A: 22 days<br>o arm B: 36 days<br>o arm C: 58 days
Nishino et al. [43]	2021	15 (15) (reactive phase)	6 (6) (reactive phase)	facial acneiform rash	reactive (firstly preventive, and if facial acneif	topical	corticosteroid (DOWN-group: ranking-down corticosteroid	corticosteroid (WEAK-group): ranking-up corticosteroid	• efficacy<br>• adherence	• incidence of grade ≥2 rash after 10 week of reactive treatment: WEAK group: 11.1% (1/9), DOWN group: 16.7% (1/6) (p = 0.8417)<br>• proportion of grade 1-2-3: grade 1: WEAK group: 55.6%, DOWN group: 50%; grade ≥3: 0% in both groups<br>• proportion of continuing EGFR therapies: 66.7% (6/9) in the WEAK group and 50% (3/6) in the DOWN group

Study	Year	Patient number of intervention group [ITT(PP)]	Patient number of control group [ITT(PP)]	Type of cutaneous adverse events	Type of intervention (preventive/reactive)	Way of administration	Intervention	Control	Endpoints	Value of endpoints
					orm rash is developed within 2 weeks, than second registration of trial is started with reactive intervention)		treatment strategy)	treatment strategies		• proportion of switching EGFR therapies: 0%

Study	Year	Patient number of intervention group [ITT(PP)]	Patient number of control group [ITT(PP)]	Type of cutaneous adverse events	Type of intervention (preventive/reactive)	Way of administration	Intervention	Control	Endpoints	Value of endpoints
Shinohara et al. [48]	2015	44 (preventive)	52 (reactive)	acneiform rash, xerosis, and paronychia	preventive and reactive (grade 2/3)	oral and topical	minocycline, heparinoid emollient, topical steroid	preventive and reactive were compared	• efficacy<br>• adherence	In the first 6 weeks:<br>• incidence of any grade acneiform rash: ($p < 0.001$ → *significant reduction)*<br>o preventive minocycline group: 47.7% (21/44)<br>o reactive minocycline group: 80.8% (42/52)<br>• incidence of grade ≥2 acneiform rash: ($p < 0.34$)<br>o preventive minocycline group: 20.5% (9/44)<br>o reactive minocycline group: 28.8% (15/52)<br>• incidence of any grade xerosis: ($p = 0.01$ → *significant reduction)*<br>o preventive minocycline group: 2.3% (1/44)<br>o reactive minocycline group: 19.2% (10/52)<br>• incidence of grade ≥2 xerosis:<br>o preventive minocycline group: 0%<br>o reactive minocycline group: 0%<br>• incidence of any grade paronychia: ($p < 0.51$)<br>o preventive minocycline group: 15.9% (7/44)<br>o reactive minocycline group: 21.2% (11/52)

Study	Year	Patient number of intervention group [ITT(PP)]	Patient number of control group [ITT(PP)]	Type of cutaneous adverse events	Type of intervention (preventive/reactive)	Way of administration	Intervention	Control	Endpoints	Value of endpoints
										• incidence of grade ≥2 paronychia: ($p < 0.58$)<br>o preventive minocycline group: 6.8% (3/44)<br>o reactive minocycline group: 5.8% (3/52)<br>• reduction of oncological therapy: ($p = 0.12$)<br>o preventive minocycline group: 0%<br>o reactive minocycline group: 5.8% (3/52)
Wang et al. [55]	2020	102 (41)‡	NA	primarily maculopapular rash, with a smaller occurrence of acneiform rash	preventive and reactive	oral and topical	Preventive: non-sedating antihistamines (Cetirizine, Loratadine).<br><br>Reactive: oral and topical antihistam	no control	efficacy	• effect of preventive antihistamines: OR for grade 1/2: 0.39 ($p = 0.09$) (43 patients)<br><br>Additional data:<br>• incidence of any grade rash: 40.2% (41/102)<br>• incidence of grade 3 rash: 18.6% (19/102)<br>• reactive treatments for grade 1/2 rash: oral antihistamine ± topical corticosteroid (63.7%, 14/22)<br>o oral antihistamines combined with topical corticosteroids: 45.5% (10/22);<br>o oral antihistamines as monotherapy: 18.2% (4/22)

Study	Year	Patient number of intervention group [ITT(PP)]	Patient number of control group [ITT(PP)]	Type of cutaneous adverse events	Type of intervention (preventive/reactive)	Way of administration	Intervention	Control	Endpoints	Value of endpoints
							ines (cetirizine, loratadine), corticosteroids (prednisone 10 or 20mg 3x/day, clobetasol, triamcinolone, fluocinonide, desoximetasone).			•reactive treatments for grade 3 rash: systemic steroids + oral antihistamines + topical steroids (63.2%, 12/19)<br>o oral antihistamines, topical and systemic steroids (63.2%);<br>o oral antihistamines with topical corticosteroids (15.8%);<br>o systemic steroids as monotherapy (15.8%)
**Studies with reactive interventions**										
Alexandrescu et	2007	11 (10)	NA	acneiform or papular eruption	reactive (grade ≥2)	topical	colloidal oatmeal lotion	no control	•efficacy	• clinical response rate: complete response: 60% (6/10); partial response: 40% (4/10)

Study	Year	Patient number of intervention group [ITT(PP)]	Patient number of control group [ITT(PP)]	Type of cutaneous adverse events	Type of intervention (preventive/reactive)	Way of administration	Intervention	Control	Endpoints	Value of endpoints
al. [10]										
Bidoli et al. [13]	2010	20 (7)	NA	papulopustular, acne-like rash primarily affecting the face, scalp, and upper torso	reactive (grade ≥2)	oral	clindamycin, isotretinoin	no control	•efficacy<br>•adherence	• clinical response (grade 2-3→grade 0-1): 86% (6/7)<br>• time to response: median 14 days (7-20)<br>• dose reduction rate of oncological therapy: 14,3% (1/7)
de Noronha e Menezes et al. [16]	2009	19 (19)	NA	rash (acneiform eruption that was characterized by erythematous	reactive (grade ≥2)	oral and topical	systemic antibiotic (doxycycline, minocycline), systemic isotretinoin and	no control	•efficacy<br>•adherence	• clinical response (within 4 weeks): complete response: 42% (8/19); partial response: 52,6% (10/19); no response: 5,3% (1/19)<br>• discontinuation rate of oncological therapy: 0%

Study	Year	Patient number of intervention group [ITT(PP)]	Patient number of control group [ITT (PP)]	Type of cutaneous adverse events	Type of intervention (preventive /reactive)	Way of administration	Intervention	Control	Endpoints	Value of endpoints
				papular and pustular lesions)			antihistamine, topical antibiotic (nadifloxacin, clindamycin) or combined with benzoyl peroxide, sunscreen, mild skin cleanser, oatmeal cream			
Duman et al. [14]	2011	10 (10)	NA	hand-foot syndrome (HFS)	reactive	oral	Vitamin E	no control	•efficacy<br>•adherence	• clinical response:<br>○ at days 20-25: complete response: 60% (6/10); partial response: 40% (4/10)

Study	Year	Patient number of intervention group [ITT(PP)]	Patient number of control group [ITT(PP)]	Type of cutaneous adverse events	Type of intervention (preventive/reactive)	Way of administration	Intervention	Control	Endpoints	Value of endpoints
										o at days 30-35: complete response: 90% (9/10); no response: 10% (1/10) (due to inappropriate vitamin E usage)<br>o at day 45: complete response: 10% (10/10)<br>• time to response: 10-12 days<br>• discontinuation rate of oncological therapy: 0%
Fernández-Guarino et al. [17]	2006	6 (6)	NA	acneiform rash	reactive (grade ≥2)	oral	doxycycline	no control	•efficacy<br>•adherence	• complete response after 3 weeks: 83.3% (5/6), partial response after 6 weeks: 16.6% (1/6)<br>• discontinuation rate of oncological therapy: 0%
Gerber et al. [19]	2012	49 (49); group 1: 21, group 2: 23, group 3: 5	NA	papulopustular rash	reactive	oral and topical	mometasone furoate, prednicarbate + nadifloxacin, prednicarbate + nadifloxac	no control	•efficacy	•change in rash severity after 3 weeks (ERSS 0->40):<br>o mometasone furoate cream: –18.9 (p = 0.00009)<br>o prednicarbate 0.25% + nadifloxacin 1% cream: –6.1 (p = 0.03)<br>o prednicarbate + nadifloxacin + systemic isotretinoin: –15.4 (p = 0.015)

Study	Year	Patient number of intervention group [ITT(PP)]	Patient number of control group [ITT(PP)]	Type of cutaneous adverse events	Type of intervention (preventive/reactive)	Way of administration	Intervention	Control	Endpoints	Value of endpoints
							in + isotretinoin			
Hwang et al. [22]	2016	52 (46)	NA	rash/desquamation, rash/acne, xerosis, pruritus, paronychia	reactive (grade ≥2)	topical	Epidermal Growth Factor (EGF) ointment (Daewoong Pharmaceuticals), containing 1 ppm of nepidermin	no control	• efficacy, QoL	• improvement of cutaneous adverse events (grade 2, 3, or 4 downgraded to ≤ grade 1 or grade 3 or 4 downgraded to grade 2 and sustained for at least 2 weeks):<br>○ rash/desquamation: 55.7% (p<0.001)<br>○ rash/acne: 44.1% (p<0.001)<br>○ xerosis: 39.4% (p=0.001)<br>○ pruritus: 55.9% (p<0.001)<br>○ paronychia: 50.0% (p=0.018)<br>• change of QoL from baseline (mean, SD; median, range):<br>○ symptoms: -17.60 ± 33.08; -10.00 (–90.00 to 40.00), emotions: -20.34 ± 29.82; -17.14 (–85.71 to 51.43), functioning: -23.04 ± 34.32; -20.00 (–100.00 to 56.00), total: -20.50 ± 29.39; -20.00 (–91.25 to 50.00)
Jornsamer et	2021	8 (8)	8 (8)	paronychia	reactive	topical	red light-emitting	the untreate	• efficacy	• mean difference (95%CI) at week 8:

Study	Year	Patient number of intervention group [ITT(PP)]	Patient number of control group [ITT(PP)]	Type of cutaneous adverse events	Type of intervention (preventive/reactive)	Way of administration	Intervention	Control	Endpoints	Value of endpoints
al. [30]							diode (LED) therapy at 633 nm wavelength, combined with standard treatments (e.g.: Proper nail care, daily iodopovidone solution soaks, topical and oral antibiotics, topical	d extremities serves as control (standard treatment alone)	•QoL	o hemoglobin level (erythema): –0.71(–1.05 to–0.36) (p <0.001)<br>o elevation volume: –0.40 (–0.49 to –0.32) (p <0.001)<br>o severity score: –2.6 (–3.91 to –1.29) (p <0.001)<br>o pain score: –1.2 (–2.40 to 0.00) (p <0.05)<br>o patient satisfaction: mean difference: +2 (intervention group: 2.6 ± 1.2, control group: 0.6 ± 1.5) (p <0.001)

Study	Year	Patient number of intervention group [ITT(PP)]	Patient number of control group [ITT(PP)]	Type of cutaneous adverse events	Type of intervention (preventive/reactive)	Way of administration	Intervention	Control	Endpoints	Value of endpoints
							corticosteroids, nail surgery, and phenol chemical matricectomy – not detailed)			
Kim E. J. et al. [32]	2020	29 (29)	NA	erythematous maculopapular rash with pruritus	reactive; (grade ≥2)	oral	prednisolone (additional antihistamines could be used)	no control	•efficacy/adherence	•treatment success rate (defined as maintenance of imatinib for more than 15 weeks after completion of the intervention period): 75.8% (22/29)
Kim Y. S. et al. [33]	2020	Arm 2: 26 (25); Arm 3: 27 (27)	Arm 1: 27 (23)	hand-foot syndrome, acneiform rash, dry skin,	reactive (grade ≥2)	topical	epidermal growth factor (EGF) ointment: EGF 1 ppm (arm	placebo (arm 1)	•efficacy<br>•QoL	•response rate (reduction from grade ≥2 to grade≤ 1 or from grade≥3 to grade 2 and lasting for at least 2 weeks) (p = .042):<br>○ EGF 1 ppm: 61.5% (16/26)<br>○ EGF 20 ppm: 77.8% (21/27)<br>○ placebo: 44.4% (12/27)

Study	Year	Patient number of intervention group [ITT(PP)]	Patient number of control group [ITT(PP)]	Type of cutaneous adverse events	Type of intervention (preventive/reactive)	Way of administration	Intervention	Control	Endpoints	Value of endpoints
				pruritus, paronychia			2); EGF 20 ppm (arm 3)			•Quality of Life (QoL) – Skindex-16: improvement (mean, SD; median, range)<br>o EGF 1 ppm: symptoms: −11.2 ± 16.; −8.3 (−45.8 - 20.8), emotions: −14.3 ± 17.2; −10.7 (−50.0 - 21.4), functioning: −8.6 ± 16.9; 0.0 (−60.0 - 6.7), composite score: −11.7 ± 14.2; −7.3 (−40.6 - 16.7)<br>o EGF 20 ppm: symptoms: −14.8 ± 19.1; −10.4 (−83.3 - 4.2), emotions: −24.8 ± 25.1; −17.9 (−83.3 - 7.1), functioning: −12.9 ± 18.4; −6.7 (−70.0 - 6.7), composite score: −18.6 ± 17.7; −12.5 (−62.5 - 5.2)<br>o placebo: symptoms: −6.9 ± 21.4; −8.3 (−50.0 - 50.0), emotions: −6.3 ± 12.4; −3.6 (−31.0 - 11.9), functioning: −2.2 ± 12.1; 0.0 (−30.0 - 30.0), composite score: −5.2 ± 8.6; −2.6 (−27.1 - 7.3)
Li et al. [35]	2013	26 (26)	NA	hand-foot skin reaction (HFSR)	reactive (grade ≥1)	topical	Topical heparin Z ointment (500 IU/1 g),	no control	•efficacy, adherence	• response rate:<br>o grade 1: 67% complete response (improved to grade 0)

Study	Year	Patient number of intervention group [ITT(PP)]	Patient number of control group [ITT(PP)]	Type of cutaneous adverse events	Type of intervention (preventive/reactive)	Way of administration	Intervention	Control	Endpoints	Value of endpoints
							combined with urea-based cream			○ grade 2: 83% improvement (improved with one grade)<br>○ grade 3: 100% improvement (improved with one grade)<br>• dose reduction rate of oncological therapy: 15,4% (4/26)
Liu T. H. et al. [36]	2022	11 (11)	NA	paronychia	reactive;	topical	surgical partial matricectomy with electrocautery ablation of the germinal matrix, combined with paraffin and	conservative treatment effectiveness: topical beta-blockers, topical povidone–iodine, topical	• efficacy	• paronychia-related wound pain score mean: ($p < 0.001$)<br>○ interventional group: 1.00 ± 0.00<br>○ control group: 2.94 ± 0.87

Study	Year	Patient number of intervention group [ITT(PP)]	Patient number of control group [ITT (PP)]	Type of cutaneous adverse events	Type of intervention (preventive /reactive)	Way of administration	Intervention	Control	Endpoints	Value of endpoints
							framycetin dressing	antibiotic ointment, and steroid ointment		
Melosky et al. [8]	2015	50 (50)	50 (50)	papulopustular/acneiform rash	reactive	oral	clindamycin + hidrocortisone	preventive treatment or no treatment	•efficacy	• overall incidence of all grades of rash (n, %):<br>o intervention (reactive – arm 2): 42 (84%)<br>o control (arm 3): 41 (82%)<br>• maximal rash grade (n, %) (p = 0.092):<br>o intervention (reactive – arm 2): grade 0/1: 28 (56%); grade 2: 18 (36%); grade 3: 4 (8%)<br>o control (arm 3): grade 0/1: 28 (56%); grade 2: 8 (16%); grade 3: 14 (28%)
Meadows et al. [40]	2015	10 (9)	NA	hand-foot syndrome	reactive (grade 1–3)	topical	1% topical sildenafil cream	placebo (contralateral side)	•efficacy	•at least one grade improvement: 0% (0/9)
Peng et al. [46]	2020	34 (33)	34 (33)	xerosis, pruritus	reactive	oral and	Zizao Yangrong Granules,	placebo group; (only	•efficacy	• change in xerosis (patient number):<br>o two weeks' treatment: (p = 0.000)

Study	Year	Patient number of intervention group [ITT(PP)]	Patient number of control group [ITT(PP)]	Type of cutaneous adverse events	Type of intervention (preventive/reactive)	Way of administration	Intervention	Control	Endpoints	Value of endpoints
						topical	a traditional Chinese medicine formulation (5 herbal), combined with topical vitamin E ointment	placebo granules alongside vitamin E ointment)	•QoL	▪ intervention: CR: 4, PR: 24, NR: 5, RR: 84.80%<br>▪ placebo: CR: 4, PR: 19, NR: 10, RR: 69.70%<br>o four weeks' treatment: (p = 0.027)<br>▪ intervention: CR: 10, PR: 18, NR: 5, RR: 84.80%<br>▪ placebo: CR: 5, PR: 20, NR: 8, RR: 75.80%<br>• change in pruritus (patient number):<br>o two weeks' treatment: (p = 0.000)<br>▪ intervention: CR: 13, PR: 8, NR: 8, RR: 72.40%<br>▪ placebo: CR: 4, PR: 14, NR: 13, RR: 58.10%<br>o four weeks' treatment: (p = 0.269)<br>▪ intervention: CR: 15, PR: 8, NR: 6, RR: 79.30%<br>▪ placebo: CR: 7, PR: 12, NR: 12, RR: 61.30%<br>• QoL (DLQI) score between two groups (mean):<br>o before treatment: 0.058<br>o two weeks' treatment: 0.016<br>o four weeks' treatment: 0.045
Rerknimitr	2019	14 (14)	14 (14)	acneiform	reactive	topical	pulsed-dye laser at	placebo (sham	•efficacy	• change in Erythema Index (mean) (week 0 → week 10): p < 0.001 - significant

Study	Year	Patient number of intervention group [ITT(PP)]	Patient number of control group [ITT(PP)]	Type of cutaneous adverse events	Type of intervention (preventive/reactive)	Way of administration	Intervention	Control	Endpoints	Value of endpoints
et al. [47]				papulopustular eruptions			595 nm wavelength	treatment with cryogen spray)	•QoL	○ intervention group: -7.2 (23.5 → 16.3)<br>○ control group: –2.98 (23.49 → 20.51)<br>• change in Lesion Count (mean) (week 0 → week 10): p > 0.05 – non-significant<br>○ intervention group: –42.17 (58.07 → 15.9)<br>○ control group: –35.52 (57.78 → 22.26)<br>• change in Physician Global Assessment (mean) (week 0 → week 10): p < 0.001 - significant<br>○ intervention group: 0 → 2.32 (moderate change)<br>○ control group: 0 → 1.17 (mild change)<br>• Patient Satisfaction (score 0-4) (week 10): p = 0.009 - significant<br>○ intervention group: 4 (IQR 3–4) – strong satisfaction<br>○ control group: 2 (IQR 2–3) – mild satisfaction
Shinohara et al. [49]	2014	18 (17)	18 (16)	hand-foot skin reaction (HFSR)	reactive (grade 1)	topical	arm A: Hydrocolloid dressing	no control	•efficacy<br>•QoL	• incidence of grade 2 or 3 in the first 4 weeks:<br>○ arm A: hydrocolloid dressing: 29.4% (5/17)<br>○ arm B: 10% urea cream: 68.8% (11/16)

Study	Year	Patient number of intervention group [ITT(PP)]	Patient number of control group [ITT(PP)]	Type of cutaneous adverse events	Type of intervention (preventive/reactive)	Way of administration	Intervention	Control	Endpoints	Value of endpoints
							with ceramide (Remois pad®); arm B: 10% urea cream (other treatments were prohibited)		•adherence	• adherence with oncological therapy:<br>o arm A: 65% (11/17)<br>o arm B: 31% (5/16)<br>• QoL assessments (partial QoL: pain assessment os the soles of feet - VAS score after 4 weeks): (p = 0.05)<br>o arm A: hydrocolloid dressing: median VAS: 2 (0–7)<br>o arm B: 10% urea cream: median VAS: 5 (1–10)
Shou et al. [51]	2021	22 (20)	NA	hand-foot skin reaction (HFSR)	reactive (grade 3)	topical	Shouzu Ning Decoction (SND), an herbal Chinese medicine decoction	no control	•efficacy<br>•QoL	• overall clinical regression: 85% (17/20)<br>o grade 3 → grade 2: 41.2% (10/17)<br>o grade 3 → grade 1: 58.8% (7/17)<br>o no improvement: 15% (3/20)<br>• QoL (HF-QoLS): significant improvement ($p < 0.01$)<br>o before treatment: 53.80 ± 4.16<br>o after treatment: 26.75 ± 11.76

Study	Year	Patient number of intervention group [ITT(PP)]	Patient number of control group [ITT(PP)]	Type of cutaneous adverse events	Type of intervention (preventive/reactive)	Way of administration	Intervention	Control	Endpoints	Value of endpoints
Shou et al. [50]	2023	102 (102)	NA	hand-foot skin reaction (HFSR),	reactive (grade 2-3)	oral and topical	three main groups: topical steroids + urea ointment; Shouzu Ning Decoction (SND); SND + urea ointment. Simultaneously treated with systemic medications (NSAID, Vitamin E)	no control	• efficacy<br>• adherence	• treatment response rate (clinical improvement from grade 2–3 to grade 1 and grade 3 to grade 2):<br>o topical steroids + urea ointment: 37.14% (13/35)<br>o SND: 65% (13/20)<br>o SND + urea ointment: 75% (9/12)<br>• discontinuation of oncological treatment (due to treatment failure of HFSR management): 41.18% (42/102)

Study	Year	Patient number of intervention group [ITT(PP)]	Patient number of control group [ITT(PP)]	Type of cutaneous adverse events	Type of intervention (preventive/reactive)	Way of administration	Intervention	Control	Endpoints	Value of endpoints
Tian et al. [52]	2017	73 (67)	37 (33)	acneiform eruption, paronychia, hand-foot skin reaction	reactive	topical	Compound Danxiong Granules (CDG) containing Chinese 5 herbs	placebo (dextrin-based powder)	• efficacy	• total effective rate (reduction in at least one grade) (p<0.0001):<br>o intervention: 77.61% (52/67)<br>o placebo: 27.27% (9/33)<br>• total effective rate on different types of skin lesions (reduction in at least one grade) (p<0.0001):<br>o HSFR<br>▪ intervention: 95.45% (21/22)<br>▪ placebo: 27.27% (3/11)<br>o acneiform eruption<br>▪ intervention: 69.23% (18/26)<br>▪ placebo: 30.78% (4/13)<br>o paronychia<br>▪ intervention: 68.42% (13/19)<br>▪ placebo: 22.22% (2/9)<br>• complete response rate:<br>o all patients with cutaneous adverse events:<br>▪ intervention: 17.91% (12/67)<br>▪ placebo: 0% (0/33)<br>o hand–foot skin reaction (p<0.0001):

Study	Year	Patient number of intervention group [ITT(PP)]	Patient number of control group [ITT(PP)]	Type of cutaneous adverse events	Type of intervention (preventive/reactive)	Way of administration	Intervention	Control	Endpoints	Value of endpoints
										▪ intervention: 31.82% (7/22)<br>▪ placebo: 0% (0/11)<br>o acneiform rash (p = 0.039):<br>▪ intervention: 11.54% (3/26)<br>▪ placebo: 0% (0/13)<br>o paronychia (p = 0.042):<br>▪ intervention: 10.53% (2/19)<br>▪ placebo: 0% (0/9)
Tohyama et al. [53]	2021	25 (21)	NA	xerotic dermatitis	reactive	oral and topical	zinc supplementation (Zinc acetate hydrate or polaprezinc) with topical corticosteroids and/or emollient	no control	• efficacy	• improvement rate: 76.2% (16/21)

Study	Year	Patient number of intervention group [ITT(PP)]	Patient number of control group [ITT(PP)]	Type of cutaneous adverse events	Type of intervention (preventive/reactive)	Way of administration	Intervention	Control	Endpoints	Value of endpoints
Vincenzi et al. [54]	2020	arm 1: 16 (12); arm 2: 14 (11)	arm 3: 14 (12)	pruritus	reactive	oral	arm 1: orvepitant 30 mg; arm 2: orvepitant 10 mg (NK1 receptor antagonist)	placebo (tablet)	•efficacy<br>•QoL	•change of NRS score at week 4 (mean (SD); median (range)):<br>o orvepitant 30 mg: −2.78 (2.64); −2.75 (−6.3 to 3.0)<br>o orvepitant 10 mg: −3.04 (3.06); −2.00 (−8.3 to 1.1)<br>o placebo: −3.21 (1.77); −2.50 (−6.3 to 0.0)<br>•VRS improvement rate:<br>o orvepitant 30 mg: week 1: 57.1% (8/14), week 4: 76.9% (10/13), week 8: 75% (9/12)<br>o orvepitant 10 mg: week 1: 64.3% (9/14), week 4: 80.0% (8/10), week 8: 81.8% (9/11)<br>o placebo: week 1: 64.3% (9/14), week 4: 78.6% (11/14), week 8: 81.8% (9/11)<br>•SKINDEX-16 change from baseline (mean, SD):<br>o week 1:<br>▪ orvepitant 30 mg: symptoms: −12.56 (22.25); emotions: −44.73 (16.35); functioning: −13.33 (16.69)

Study	Year	Patient number of intervention group [ITT(PP)]	Patient number of control group [ITT(PP)]	Type of cutaneous adverse events	Type of intervention (preventive/reactive)	Way of administration	Intervention	Control	Endpoints	Value of endpoints
										▪ orvepitant 10 mg: symptoms: 1.24 (25.05); emotions: −21.52 (27.80); functioning: −12.05 (29.08)<br>▪ placebo: symptoms: −2.88 (18.50); emotions: −21.29 (18.20) −1.28 (13.51); functioning:<br>o week 4:<br>▪ orvepitant 30 mg: symptoms: −8.29 (25.06); emotions: −32.60 (24.96); functioning: 2.05 (26.51)<br>▪ orvepitant 10 mg: symptoms: −9.09 (36.94); emotions: −31.96 (36.34); functioning: −11.51 (36.07)<br>▪ placebo: symptoms: −7.69 (29.56); emotions: −25.69 (26.54); functioning: −1.80 (20.40)<br>o week 8:<br>▪ orvepitant 30 mg: symptoms: −8.09 (23.60); emotions: −35.37 (30.88); functioning: −0.83 (28.04)<br>▪ orvepitant 10 mg: symptoms: −8.71 (36.19); emotions: −37.23 (28.13); functioning: −22.73 (25.64)

Study	Year	Patient number of intervention group [ITT(PP)]	Patient number of control group [ITT(PP)]	Type of cutaneous adverse events	Type of intervention (preventive/reactive)	Way of administration	Intervention	Control	Endpoints	Value of endpoints
										▪ placebo: symptoms: −4.02 (33.30); emotions: −36.86 (26.32); functioning: −5.00 (16.17)
Wong et al. [57]	2010	20 (13)	NA	papulopustular rash (acneiform eruption), primarily on face	reactive (grade ≥2)	topical	Regenecare Wound Gel containing 2% lidocaine, collagen, vitamin E, and aloe vera	left side of face served as untreated control for the first week; then treated with gel after crossover	•efficacy<br>•adherence	•improvement in pruritus at week 1 (patient self-reported):<br>o intervention: 69% (9/13), worsen: 8% (1/13), no difference: 23% (3/13) (p = 0.01 / 0.006)<br>o control: no significant improvement<br>•improvement in pain:<br>o intervention: 54% (7/13), worsen: 8% (1/13), no difference: 38% (5/13) (p = 0.12 / 0.16)<br>o control: no significant improvement<br>•dose modification of oncological therapy: 31% (4/13)
Wu et al. [58]	2021	135 (107, with	NA	multiple dermatologic	reactive	oral and	treatments varied by cutaneous	no endpoint	•efficacy	•not explicit data<br>•acneiform rash:

Study	Year	Patient number of intervention group [ITT(PP)]	Patient number of control group [ITT (PP)]	Type of cutaneous adverse events	Type of intervention (preventive /reactive)	Way of administration	Intervention	Control	Endpoints	Value of endpoints
		adverse events)		adverse events (dAEs): acneiform rash, maculopapular rash, rash unspecified, pruritus, dry skin, photosensitivity, alopecia, erythema multiforme		topical	adverse events type: • oral steroid, antibiotic, pregabalin, antihistamine topical steroid, antibiotic, sunscreen			○ Treated with topical steroids + topical antibiotics (± oral doxycycline/minocycline)<br>○ 93% (14/15) received oral antibiotics<br>○ Most responded well; some (5/13) required dose reduction<br>• maculopapular rash:<br>○ 38% (5/13) treated with short-term oral steroids (prednizon 0.5 mg/kg/day for 7-10 day); some needed ulixertinib dose reduction<br>○ Others (8/13) treated only effectively with topical steroids<br>• pruritus:<br>○ Treated with topical steroids (twice a day) + oral antihistamines (four times a day)<br>○ In 2 cases: oral steroids (40 mg/day) + pregabalin (50–75 mg two or three times a day for 5–10 nap) led to resolution
Yen et al. [60]	2020	35 (35)	NA	severe paronychia with	reactive	topical	betaxolol 0.25% ophthalmi	no control	•efficacy	•complete resolution after 12 weeks: 97.1% (34/35)<br>•partial resolution after 12 weeks: 2.9% (1/35)

Study	Year	Patient number of intervention group [ITT(PP)]	Patient number of control group [ITT (PP)]	Type of cutaneous adverse events	Type of intervention (preventive/reactive)	Way of administration	Intervention	Control	Endpoints	Value of endpoints
				pyogenic granuloma-like lesions			c solution, applied topically		•adherence	•reduction in CTCAE paronychia severity score after 4 weeks: from 2.29 to 0.63 at 4 weeks ($p = 5.55 \times 10^{-16}$)<br>•pain reduction (VAS scale) within 1 week: from 7.06 to 2.26 ($p = 6.11 \times 10^{-25}$)<br>•time to fissure healing: all patients recovered; 3 within 3 days, the rest within 1 week<br>•discontinuation or dose reduction of oncological therapy: 0%
Zhang et al. [61]	2022	29 (27)	29 (27)	papulopustular rash, xerosis, paronychia	reactive	oral and topical	Topical Zhiyang Pingfu Liquid containing 5 traditional Chinese medicinal herbs.	placebo (solution with similar appearance)	•efficacy	•effective rate of rash (p = 0.040):<br>o intervention group: 81.48% (22/27)<br>o control group: 55.56% (15/27)<br>•pruritus improvement rate (p = 0.033):<br>o intervention group: 100.00% (27/27)<br>o control group: 82.61% (22/27)<br>•paronychia improvement rate (p > 0.05):<br>o intervention group: 76.92% (20/26)<br>o control group: 68.42% (18/26)<br>•xerosis improvement rate (p > 0.05):<br>o intervention group: 80.77% (21/26)

Study	Year	Patient number of intervention group [ITT(PP)]	Patient number of control group [ITT (PP)]	Type of cutaneous adverse events	Type of intervention (preventive /reactive)	Way of administration	Intervention	Control	Endpoints	Value of endpoints
										○ control group: 70.37% (19/26)

Adherence: adherence to the oncological therapy; **CHL:** chloramphenicol; **CR:** complete respone (cured); **DLQI:** dermatology life quality index; **EI:** erythema index; **EORTC-QLQ C30/L13:** European Organization for the Research and Treatment of Cancer Quality of Life Questionnaire-Lung Cancer; **ERSS or WoMoScore**: EGFRI induced rash severity score; **EWB:** emotional well-being; **FACT-L:** Functional Assessment of Cancer Therapy-Lung; **FWB:** functional well-being; **HFSR:** hand-foot skin reaction; **HR:** hazard ratio; **IGA:** Investigator's Global Assessment scale; **LCS:** Lung Cancer Subscale; **MASCC:** multinational association of supportive care in cancer grading system; **NCI-CTCAE:** National Cancer Institute - Common Terminology Criteria for Adverse Events; **NOL**: number of lesions; **NSAID:** non-steroidal anti-inflammatory drug; **NR:** no response; **NRS:** Patient-reported Numerical Rating Scale; **PGA:** physician global assessment; **PP:** per protocol; **PR:** partial response (side effect are reduced by 1 grade); **PRED:** prednisolone; **PWB:** physical well-being; **SND:** Shouzu Ning Decoction; **SWB:** social/family well-being; **RR:** response rate; **QoL:** Quality of life; **VAS:** visual analogue scale; **VRS:** Verbal Rating Scale

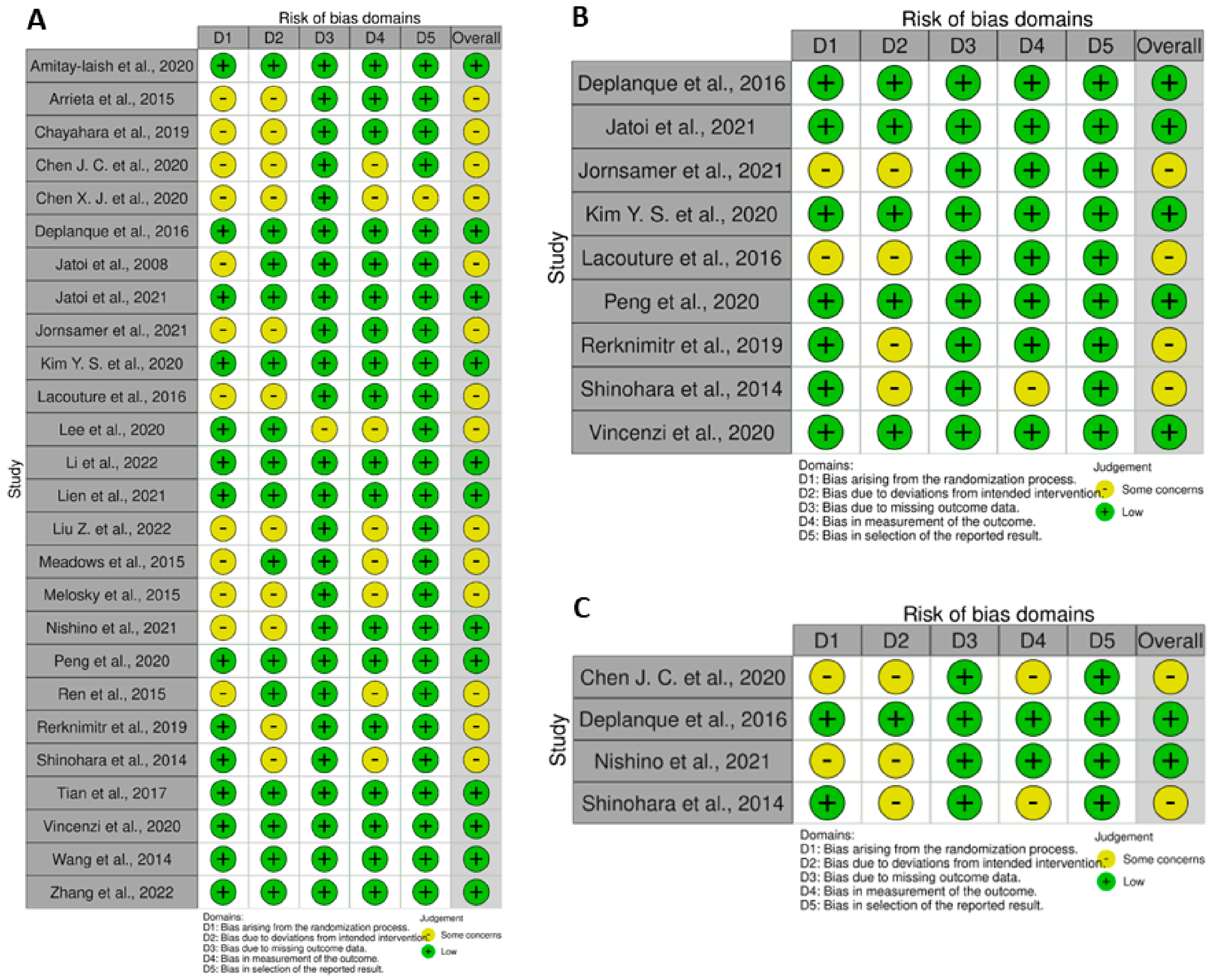

Figure S2. Risk of bias assessment with RoB2 tool of randomized controlled trials of interventions for outcomes of (A) efficacy, (B) quality of life, (C) adherence to oncological therapy

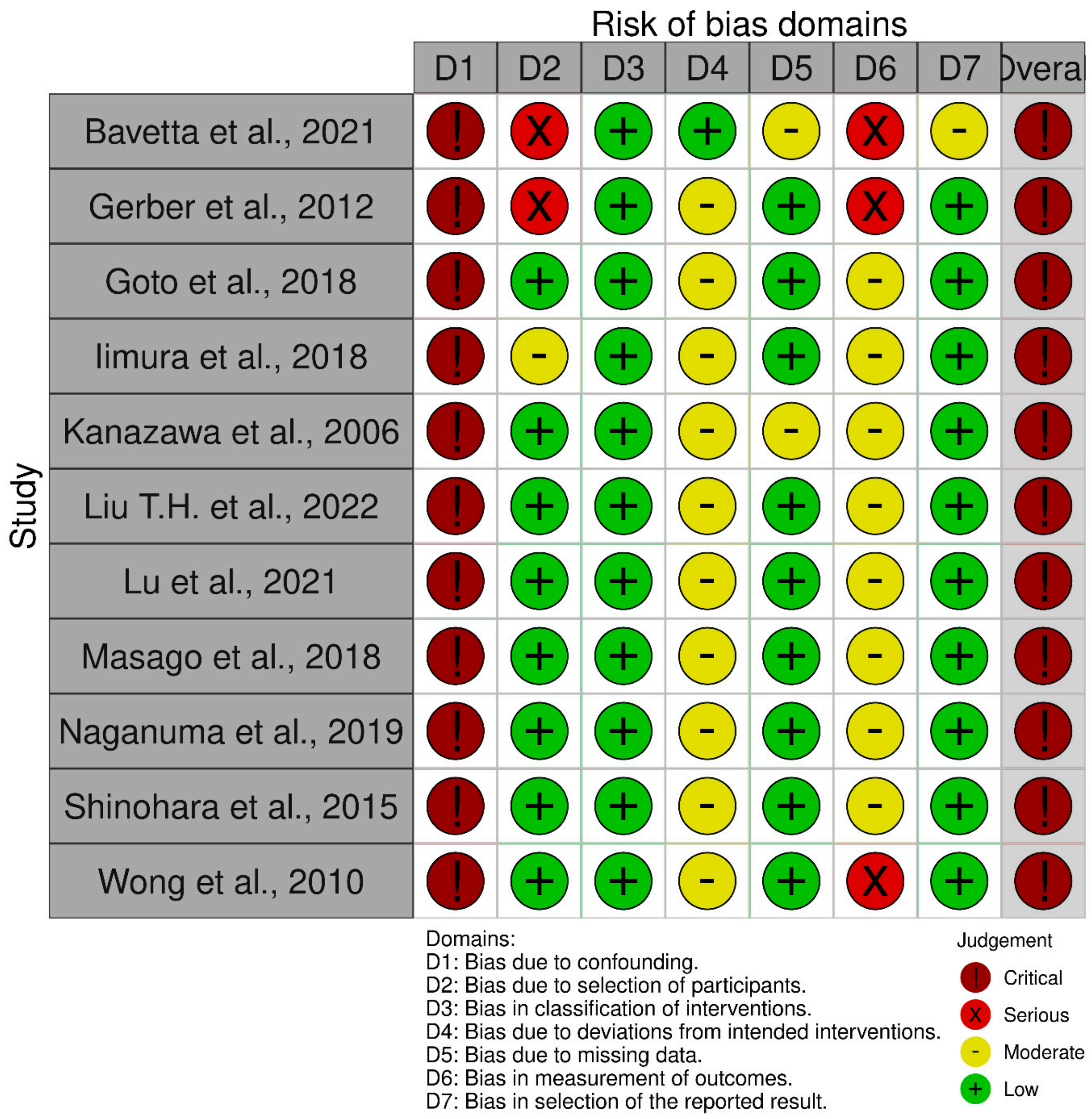

Figure S3. Risk of bias assessment with ROBINS-I tool of non-randomized more-arm studies

Table S3. Risk of bias assessment with MINORS tool of non-randomized single-arm studies

Studies	A clearly stated aim	Inclusion of consecutive patients	Prospective collection of data	Endpoints appropriate to the aim of the	Unbiased assessment of the study	Follow-up period appropriate to the aim	Loss to follow up less than 5%	Prospective calculation of the study size	Overall score
Alexandrescu et al., 2007	2	0	0	2	0	2	2	0	8
Bidoli et al., 2010	2	0	0	2	0	2	2	0	8
De Noronha E Menezes et al., 2009	2	0	0	2	0	2	2	0	8
Duman et al., 2011	2	0	0	2	0	2	2	0	8
Fernández-Guarino et al., 2006	2	0	0	2	0	2	2	0	8
Fuggetta et al., 2019	2	0	1	2	0	2	2	0	9
Grande et al., 2013	2	0	2	2	0	2	2	2	12
Hwang et al., 2016	2	0	2	2	0	2	1	2	11
Ichiki et al., 2017	2	0	2	2	0	2	2	2	12
Ishikawa et al., 2017	2	0	0	2	0	2	2	0	8
Iwasaku et al., 2019;2023	2	0	2	2	0	2	2	2	12
Kim E. J. et al., 2020	2	0	2	2	0	2	2	2	12
Li et al., 2013	2	0	0	2	0	2	2	0	8
Melosky et al., 2019	2	0	2	2	0	2	1	2	11

Nishizawa et al., 2023	2	0	2	2	1	2	2	2	13
Okajima et al., 2021	2	0	2	2	0	2	2	2	12
Shou et al., 2021	2	0	0	2	0	2	2	0	8
Shou et al., 2023	2	0	0	2	0	2	1	0	7
Tohyama et al., 2021	2	0	0	2	0	2	1	0	7
Wang et al., 2020	2	0	0	2	0	2	1	0	7
Wu et al., 2021	2	0	2	2	0	2	1	0	9
Yamamoto et al., 2022	2	0	2	2	0	2	2	2	12
Yen et al., 2020	2	0	0	2	0	2	2	0	8

Table S4. GRADE assessment for controlled studies

Prevention and treatment compared to no prevention, no treatment, placebo for cutaneous adverse events induced by the kinase inhibitory oncological targeted therapy

Patient or population: cutaneous adverse events induced by the kinase inhibitory oncological targeted therapy

Setting:

Intervention: prevention and treatment

Comparison: no prevention, no treatment, placebo

Outcomes	№ of participants (studies) Follow-up	Certainty of the evidence (GRADE)	Relative effect (95% CI)	Anticipated absolute effects	
				Risk with no prevention, no treatment, placebo	Risk difference with prevention and treatment
Incidence in grade 1-4 adverse events (systemic preventions) (Incidence) assessed with: Odds ratio	567 (5 RCTs)	⊕◯◯◯ Very low	**OR 0.70** (0.48 to 1.02)	730 per 1000	**76 fewer per 1000** (165 fewer to 4 more)
Incidence in grade 1-4 adverse events (topical preventions) (Incidence) assessed with: Odds ratio	1343 (5 RCTs)	⊕◯◯◯ Very low	**OR 0.55** (0.38 to 0.80)	691 per 1000	**139 fewer per 1000** (232 fewer to 50 fewer)
Incidence in grade 3-4 adverse events (systemic preventions) (Incidence) assessed with: Odds ratio	567 (5 RCTs)	⊕◯◯◯ Very low	**OR 0.24** (0.14 to 0.41)	144 per 1000	**105 fewer per 1000** (121 fewer to 79 fewer)
Incidence in grade 3-4 adverse events (topical preventions) (Incidence) assessed with: Odds ratio	1231 (4 RCTs)	⊕◯◯◯ Very low	**OR 0.74** (0.54 to 1.00)	70 per 1000	**17 fewer per 1000** (31 fewer to 0 fewer)

***The risk in the intervention group** (and its 95% confidence interval) is based on the assumed risk in the comparison group and the **relative effect** of the intervention (and its 95% CI).

CI: confidence interval; **OR:** odds ratio

Prevention and treatment compared to no prevention, no treatment, placebo for cutaneous adverse events induced by the kinase inhibitory oncological targeted therapy

Patient or population: cutaneous adverse events induced by the kinase inhibitory oncological targeted therapy

Setting:

Intervention: prevention and treatment

Comparison: no prevention, no treatment, placebo

Outcomes	№ of participants (studies) Follow-up	Certainty of the evidence (GRADE)	Relative effect (95% CI)	Anticipated absolute effects	
				Risk with no prevention, no treatment, placebo	Risk difference with prevention and treatment

GRADE Working Group grades of evidence
High certainty: we are very confident that the true effect lies close to that of the estimate of the effect.
Moderate certainty: we are moderately confident in the effect estimate: the true effect is likely to be close to the estimate of the effect, but there is a possibility that it is substantially different.
Low certainty: our confidence in the effect estimate is limited: the true effect may be substantially different from the estimate of the effect.
Very low certainty: we have very little confidence in the effect estimate: the true effect is likely to be substantially different from the estimate of effect.

1. Arrieta, O.; Vega-González, M.T.; López-Macías, D.; Martínez-Hernández, J.N.; Bacon-Fonseca, L.; Macedo-Pérez, E.O.; Ramírez-Tirado, L.A.; Flores-Estrada, D.; de la Garza-Salazar, J. Randomized, open-label trial evaluating the preventive effect of tetracycline on afatinib induced-skin toxicities in non-small cell lung cancer patients. *Lung cancer (Amsterdam, Netherlands)* **2015**, *88*, 282-288, doi:10.1016/j.lungcan.2015.03.019.
2. Chen, J.C.; Wang, J.C.; Pan, Y.X.; Yi, M.J.; Chen, J.B.; Wang, X.H.; Fu, Y.Z.; Zhang, Y.J.; Xu, L.; Chen, M.S.; et al. Preventive effect of celecoxib in sorafenib-related hand-foot syndrome in hepatocellular carcinoma patients, a single-center, open-label, randomized, controlled clinical phase III trial. *American journal of cancer research* **2020**, *10*, 1467-1476.
3. Chen, X.; Jiang, Y.; Zhang, Y.; Dai, W.; Fan, R.; Weng, X.; He, P.; Yan, F.; Guo, Y. Effect of ozone oil for prevention and treatment of sorafenib-induced hand-foot skin reactions: A randomized controlled trial. J. South. Med. Univ. **2020**, *40*, 1488-1492, doi:10.12122/j.issn.1673-4254.2020.10.15.
4. Deplanque, G.; Gervais, R.; Vergnenegre, A.; Falchero, L.; Souquet, P.J.; Chavaillon, J.M.; Taviot, B.; Fraboulet, G.; Saal, H.; Robert, C.; et al. Doxycycline for prevention of erlotinib-induced rash in patients with non-small-cell lung cancer (NSCLC) after failure of first-line chemotherapy: a randomized, open-label trial. *Journal of the American Academy of Dermatology* **2016**, *74*, 1077-1085, doi:10.1016/j.jaad.2016.01.019.
5. Lacouture, M.E.; Keefe, D.M.; Sonis, S.; Jatoi, A.; Gernhardt, D.; Wang, T.; Doherty, J.P.; Giri, N.; Nadanaciva, S.; O'Connell, J.; et al. A phase II study (ARCHER 1042) to evaluate prophylactic treatment of dacomitinib-induced dermatologic and gastrointestinal adverse events in advanced non-small-cell lung cancer. *Annals of oncology* **2016**, *27*, 1712-1718, doi:10.1093/annonc/mdw227.
6. Lee, Y.S.; Jung, Y.K.; Kim, J.H.; Cho, S.B.; Kim, D.Y.; Kim, M.Y.; Kim, H.J.; Seo, Y.S.; Yoon, K.T.; Hong, Y.M.; et al. Effect of urea cream on sorafenib-associated hand-foot skin reaction in patients with hepatocellular carcinoma: a multicenter, randomised, double-blind controlled study. *European journal of cancer (Oxford, England : 1990)* **2020**, *140*, 19-27, doi:10.1016/j.ejca.2020.09.012.
7. Lien, R.Y.; Tung, H.H.; Wu, S.L.; Hu, S.H.; Lu, L.C.; Lu, S.F. Validation of the prophylactic efficacy of urea-based creams on sorafenib-induced hand-foot skin reaction in patients with advanced hepatocellular carcinoma: a randomised experiment study. *Cancer reports* **2021**, doi:10.1002/cnr2.1532.
8. Melosky, B.; Anderson, H.; Burkes, R.L.; Chu, Q.; Hao, D.; Ho, V.; Ho, C.; Lam, W.; Lee, C.W.; Leighl, N.B.; et al. Pan Canadian Rash Trial: a Randomized Phase III Trial Evaluating the Impact of a Prophylactic Skin Treatment Regimen on Epidermal Growth Factor Receptor-Tyrosine Kinase Inhibitor-Induced Skin Toxicities in Patients With Metastatic Lung Cancer. *Journal of clinical oncology* **2016**, *34*, 810-815, doi:10.1200/JCO.2015.62.3918.
9. Ren, Z.; Zhu, K.; Kang, H.; Lu, M.; Qu, Z.; Lu, L.; Song, T.; Zhou, W.; Wang, H.; Yang, W.; et al. Randomized controlled trial of the prophylactic effect of urea-based cream on sorafenib-associated hand-foot skin reactions in patients with advanced hepatocellular carcinoma. *Journal of clinical oncology* **2015**, *33*, 894-900, doi:10.1200/JCO.2013.52.9651.
10. Alexandrescu, D.T.; Vaillant, J.G.; Dasanu, C.A. Effect of treatment with a colloidal oatmeal lotion on the acneform eruption induced by epidermal growth factor receptor and multiple tyrosine-kinase inhibitors. *Clin Exp Dermatol* **2007**, *32*, 71-74, doi:10.1111/j.1365-2230.2006.02285.x.
11. Amitay-Laish, I.; Prag-Naveh, H.; Ollech, A.; Davidovici, B.; Leshem, Y.A.; Snast, I.; Popovtzer, A.; Purim, O.; Flex, D.; David, M.; et al. Prophylactic Topical Treatment for EGFR Inhibitor-Induced Papulopustular Rash: a Randomized Clinical Trial. *Dermatology (Basel, Switzerland)* **2021**, *237*, 988-994, doi:10.1159/000511869.
12. Bavetta, M.; Silvaggio, D.; Campione, E.; Sollena, P.; Formica, V.; Coletta, D.; Graziani, G.; Romano, M.C.P.; Roselli, M.; Peris, K.; et al. The effects of association of topical polydatin improves the preemptive systemic treatment on EGFR inhibitors cutaneous adverse reactions. *Journal of Clinical Medicine* **2021**, *10*, 1-8, doi:10.3390/jcm10030466.
13. Bidoli, P.; Cortinovis, D.L.; Colombo, I.; Crippa, A.; Cicchiello, F.; Villa, F.; Cazzaniga, M.E.; Altomare, G. Isotretinoin plus clindamycin seem highly effective against severe erlotinib-induced skin rash in advanced non-small cell lung cancer. *J Thorac Oncol* **2010**, *5*, 1662-1663, doi:10.1097/JTO.0b013e3181ec1729.
14. Bozkurt Duman, B.; Kara, B.; Oguz Kara, I.; Demiryurek, H.; Aksungur, E. Hand-foot syndrome due to sorafenib in hepatocellular carcinoma treated with vitamin E without dose modification; a preliminary clinical study. *J buon* **2011**, *16*, 759-764.
15. Chayahara, N.; Mukohara, T.; Tachihara, M.; Fujishima, Y.; Fukunaga, A.; Washio, K.; Yamamoto, M.; Nakata, K.; Kobayashi, K.; Takenaka, K.; et al. Adapalene Gel 0.1% Versus Placebo as Prophylaxis for Anti-Epidermal Growth Factor Receptor-Induced Acne-Like Rash: a Randomized Left-Right Comparative Evaluation (APPEARANCE). *Oncologist* **2019**, *24*, 885-e413, doi:10.1634/theoncologist.2019-0156.
16. de Noronha e Menezes, N.M.; Lima, R.; Moreira, A.; Varela, P.; Barroso, A.; Baptista, A.; Parente, B. Description and management of cutaneous side effects during erlotinib and cetuximab treatment in lung and colorectal cancer patients: a prospective and descriptive study of 19 patients. *Eur J Dermatol* **2009**, *19*, 248-251, doi:10.1684/ejd.2009.0650.
17. Fernández-Guarino, M.; Pérez García, B.; Aldanondo Fernández de la Mora, I.; García-Millán, C.; Garrido López, P.; Jaén Olasolo, P. [Treatment of acneiform rash by epidermal growth factor inhibitors with oral tetracyclines]. *Actas Dermosifiliogr* **2006**, *97*, 503-508, doi:10.1016/s0001-7310(06)73451-2.
18. Fuggetta, M.P.; Migliorino, M.R.; Ricciardi, S.; Osman, G.; Iacono, D.; Leone, A.; Lombardi, A.; Ravagnan, G.; Greco, S.; Remotti, D.; et al. Prophylactic Dermatologic Treatment of Afatinib-Induced Skin Toxicities in Patients with Metastatic Lung Cancer: A Pilot Study. *Scientifica (Cairo)* **2019**, *2019*, 9136249, doi:10.1155/2019/9136249.
19. Gerber, P.A.; Meller, S.; Eames, T.; Buhren, B.A.; Schrumpf, H.; Hetzer, S.; Ehmann, L.M.; Budach, W.; Bölke, E.; Matuschek, C.; et al. Management of EGFR-inhibitor associated rash: a retrospective study in 49 patients. *Eur J Med Res* **2012**, *17*, 4, doi:10.1186/2047-783x-17-4.
20. Goto, A.; Ozawa, Y.; Koda, K.; Akahori, D.; Koyauchi, T.; Amano, Y.; Kakutani, T.; Sato, Y.; Hasegawa, H.; Matsui, T.; et al. Clinical impact of minocycline on afatinib-related rash in patients with non-small cell lung cancer harboring epidermal growth factor receptor mutations. *Respir Investig* **2018**, *56*, 179-183, doi:10.1016/j.resinv.2017.11.009.
21. Grande, R.; Narducci, F.; Bianchetti, S.; Mansueto, G.; Gemma, D.; Sperduti, I.; Trombetta, G.; Angelini, F.; Gamucci, T. Pre-emptive skin toxicity treatment for anti-EGFR drugs: evaluation of efficacy of skin moisturizers and lymecycline. A phase II study. *Support Care Cancer* **2013**, *21*, 1691-1695, doi:10.1007/s00520-012-1715-1.
22. Hwang, I.G.; Kang, J.H.; Oh, S.Y.; Lee, S.; Kim, S.H.; Song, K.H.; Son, C.; Park, M.J.; Kang, M.H.; Kim, H.G.; et al. Phase II trial of epidermal growth factor ointment for patients with Erlotinib-related skin effects. *Supportive care in cancer* **2016**, *24*, 301-309, doi:10.1007/s00520-015-2783-9.
23. Ichiki, M.; Wataya, H.; Yamada, K.; Tsuruta, N.; Takeoka, H.; Okayama, Y.; Sasaki, J.; Hoshino, T. Preventive effect of kampo medicine (hangeshashin-to, TJ-14) plus minocycline against afatinib-induced diarrhea and skin rash in patients with non-small cell lung cancer. *Onco Targets Ther* **2017**, *10*, 5107-5113, doi:10.2147/ott.S145613.
24. Iimura, Y.; Shimomura, H.; Yasu, T.; Imanaka, K.; Ogawa, R.; Ito, A.; Suzuki, K.; Yamaguchi, G.; Kawasaki, N.; Konaka, C. NSAIDs may prevent EGFR-TKI-related skin rash in non-small cell lung cancer patients *Int J Clin Pharmacol Ther* **2018**, *56*, 551-554, doi:10.5414/cp203323.
25. Ishikawa, H.; Hamauchi, S.; Tanaka, R.; Shino, M.; Yamazaki, K. Effectiveness of topical steroid therapy for prevention of Regorafenib associated Hand-foot Skin Reaction. *Journal of Applied Pharmacy* **2017**, *9*, doi:10.21065/1920-4159.1000248.
26. Iwasaku, M.; Uchino, J.; Chibana, K.; Tanzawa, S.; Yamada, T.; Tobino, K.; Uchida, Y.; Kijima, T.; Nakatomi, K.; Izumi, M.; et al. Prophylactic treatment of dacomitinib-induced skin toxicities in epidermal growth factor receptor-mutated non–small-cell lung cancer: A multicenter, Phase II trial. *Cancer Medicine* **2023**, *12*, 15117-15127, doi:10.1002/cam4.6184.
27. Iwasaku, M.; Uchino, J.; Yamada, T.; Chihara, Y.; Shimamoto, T.; Tamiya, N.; Kaneko, Y.; Kiyomi, F.; Takayama, K. Rationale and design of a phase II study to evaluate prophylactic treatment of dacomitinib-induced dermatologic adverse events in epidermal growth factor receptor-mutated advanced non-small cell lung cancer (SPIRAL-Daco study). *Transl Lung Cancer Res* **2019**, *8*, 519-523, doi:10.21037/tlcr.2019.08.03.
28. Jatoi, A.; Ou, F.S.; Ahn, D.H.; Zemla, T.J.; Le-Rademacher, J.G.; Boland, P.; Ciombor, K.K.; Jacobs, N.L.; Pasche, B.; Cleary, J.M.; et al. Preemptive Versus Reactive Topical Clobetasol for Regorafenib-Induced Hand-Foot Reactions: a Preplanned Analysis of the ReDOS Trial. *Oncologist* **2021**, *26*, 610-618, doi:10.1002/onco.13730.
29. Jatoi, A.; Rowland, K.; Sloan, J.A.; Gross, H.M.; Fishkin, P.A.; Kahanic, S.P.; Novotny, P.J.; Schaefer, P.L.; Johnson, D.B.; Tschetter, L.K.; et al. Tetracycline to prevent epidermal growth factor receptor inhibitor-induced skin rashes: results of a placebo-controlled trial from the North Central Cancer Treatment Group (N03CB). *Cancer* **2008**, *113*, 847-853, doi:10.1002/cncr.23621.
30. Jornsamer, C.; Theerawattanawit, C.; Wichayachakorn, C.; Panchaprateep, R.; Kerr, S.J.; Asawanonda, P.; Rerknimitr, P. Red light emitting diode as an adjuvant treatment for epidermal growth factor receptor inhibitors-induced paronychia. *Journal of dermatological treatment* **2022**, *33*, 1990-1994, doi:10.1080/09546634.2021.1927950.
31. Kanazawa, S.; Yamaguchi, K.; Kinoshita, Y.; Muramatsu, M.; Komiyama, Y.; Nomura, S. Aspirin reduces adverse effects of gefitinib. *Anticancer Drugs* **2006**, *17*, 423-427, doi:10.1097/01.cad.0000203385.45163.76.
32. Kim, E.J.; Ryu, M.H.; Park, S.R.; Beck, M.Y.; Lee, W.J.; Lee, M.W.; Kang, Y.K. Systemic Steroid Treatment for Imatinib-Associated Severe Skin Rash in Patients with Gastrointestinal Stromal Tumor: A Phase II Study. *Oncologist* **2020**, *25*, e1785-e1793, doi:10.1634/theoncologist.2019-0953.
33. Kim, Y.S.; Ji, J.H.; Oh, S.Y.; Lee, S.; Huh, S.J.; Lee, J.H.; Song, K.H.; Son, C.H.; Roh, M.S.; Lee, G.W.; et al. A Randomized Controlled Trial of Epidermal Growth Factor Ointment for Treating Epidermal Growth Factor Receptor Inhibitor-Induced Skin Toxicities. *Oncologist* **2020**, *25*, e186-e193, doi:10.1634/theoncologist.2019-0221.
34. Li, C.L.; Hsia, T.C.; Yang, S.T.; Chao, K.C.; Tu, C.Y.; Chen, H.J.; Li, C.H. Efficacy of Prophylactic Traditional Chinese Medicine on Skin Toxicity of Afatinib in EGFR Mutation-Positive Advanced Lung Adenocarcinoma: a Single-Center, Prospective, Double-Blinded, Randomized-Controlled Pilot Trial. *Integrative cancer therapies* **2022**, *21*, 15347354221086663, doi:10.1177/15347354221086663.
35. Li, J.R.; Yang, C.R.; Cheng, C.L.; Ho, H.C.; Chiu, K.Y.; Su, C.K.; Chen, W.M.; Wang, S.S.; Chen, C.S.; Yang, C.K.; et al. Efficacy of a protocol including heparin ointment for treatment of multikinase inhibitor-induced hand-foot skin reactions. *Support Care Cancer* **2013**, *21*, 907-911, doi:10.1007/s00520-012-1693-3.
36. Liu, T.H.; Wu, S.Y.; Tsai, S.L.; Wu, C.W.; Hou, C.C.; Lai, C.Y.; Tzeng, Y.S. Effective and enduring surgical treatment for targeted therapy-related paronychia: A retrospective study. *Medicine (United States)* **2022**, *101*, E31208, doi:10.1097/MD.0000000000031208.
37. Liu, Z.; Tian, T.; Wang, B.; Lu, D.; Ruan, J.; Shan, J. Reducing Acneiform Rash Induced by EGFR Inhibitors With Honeysuckle Therapy: a Prospective, Randomized, Controlled Study. *Frontiers in pharmacology* **2022**, *13*, 835166, doi:10.3389/fphar.2022.835166.
38. Lu, C.W.; Wang, T.Y.; Yen, C.F.; Chen, K.H.; Wu, C.E.; Wang, C.L.; Hsu, P.C.; Fang, Y.F.; Li, S.H.; Ko, H.W.; et al. Using betaxolol for the prevention of paronychia induced by epidermal growth factor receptor inhibitors: a case–control cohort study. *International Journal of Dermatology* **2021**, *60*, 179-184, doi:10.1111/ijd.15099.
39. Masago, K.; Imamichi, F.; Masuda, Y.; Ariga, N.; Fujitomi, K.; Fukumine, Y.; Hatakenaka, K.; Fujita, S.; Katakami, N. Team Management of Skin Rash Associated with Use of Epidermal Growth Factor Receptor-Tyrosine Kinase Inhibitors. *Asia Pac J Oncol Nurs* **2018**, *5*, 430-434, doi:10.4103/apjon.apjon_33_18.
40. Meadows, K.L.; Rushing, C.; Honeycutt, W.; Latta, K.; Howard, L.; Arrowood, C.A.; Niedzwiecki, D.; Hurwitz, H.I. Treatment of palmar-plantar erythrodysesthesia (PPE) with topical sildenafil: a pilot study. *Supportive care in cancer* **2015**, *23*, 1311-1319, doi:10.1007/s00520-014-2465-z.
41. Melosky, B.L.; Lim, H.J.; Davies, J.M.; Gill, S.; Kollmannsberger, C.K.; Ho, M.Y.; Vandt, S.A.; Renouf, D.J. A phase II trial of the effect of perindopril on hand–foot skin reaction (HFSR) incidence and severity in patients receiving regorafenib for refractory mCRC. *Cancer Chemotherapy and Pharmacology* **2019**, *83*, 411-417, doi:10.1007/s00280-018-3738-x.
42. Naganuma, A.; Hoshino, T.; Ohno, N.; Ogawa, Y.; Murakami, T.; Horiguchi, S.; Uehara, D.; Suzuki, Y.; Hatanaka, T.; Kudo, T.; et al. β-Hydroxy-β-methyl Butyrate/L-Arginine/L-Glutamine Supplementation for Preventing Hand-Foot Skin Reaction in Sorafenib for Advanced Hepatocellular Carcinoma. *In Vivo* **2019**, *33*, 155-161, doi:10.21873/invivo.11452.
43. Nishino, K.; Fujiwara, Y.; Ohe, Y.; Saito, R.; Miyauchi, E.; Kobayashi, T.; Nakai, Y.; Takahashi, T.; Shibata, T.; Hamaguchi, T.; et al. Results of the non-small cell lung cancer part of a phase III, open-label, randomized trial evaluating topical corticosteroid therapy for facial acneiform dermatitis induced by EGFR inhibitors: stepwise rank down from potent corticosteroid (FAEISS study, NCCH-1512). *Supportive care in cancer* **2021**, *29*, 2327-2334, doi:10.1007/s00520-020-05765-7.
44. Nishizawa, A.; Shinozaki, E.; Wakatsuki, T.; Satoh, T.; Yamazaki, N.; Oyamada, S.; Ariyoshi, K.; Kihara, K.; Tsuboi, M.; Yamaguchi, K. Efficacy of aluminum chloride in severe regorafenib-associated hand-foot skin reactions: a single-arm trial. *BMC Cancer* **2023**, *23*, 401, doi:10.1186/s12885-023-10864-9.
45. Okajima, M.; Miura, S.; Watanabe, S.; Tanaka, H.; Ito, K.; Ishida, T.; Makino, M.; Iwashima, A.; Matsumoto, N.; Sato, K.; et al. A prospective phase II study of multimodal prophylactic treatment for afatinib-induced adverse events in advanced non-small cell lung cancer (Niigata Lung Cancer Treatment Group 1401). *Transl Lung Cancer Res* **2021**, *10*, 252-260, doi:10.21037/tlcr-20-649.
46. Peng, Y.M.; Duan, H.; Zhang, J.; Sun, C.; Zhang, X.; Shen, W.; Zheng, S.; Tan, K.; Jiang, X.; Li, J.; et al. Application of Zizao Yangrong Granules for Treating Targeted Drugs-Related Skin Xerosis: a Randomized Double-Blinded Controlled Study. *Integrative cancer therapies* **2020**, *19*, 1534735420924832, doi:10.1177/1534735420924832.
47. Rerknimitr, P.; Suphankong, Y.; Panchaprateep, R.; Kerr, S.J.; Asawanonda, P. Pulsed-dye laser as an adjuvant treatment for papulopustular eruptions from epidermal growth factor receptor inhibitors, a randomized blinded split-faced controlled trial. *Lasers in surgery and medicine* **2019**, *51*, 584-591, doi:10.1002/lsm.23080.
48. Shinohara, A.; Ikeda, M.; Okuyama, H.; Kobayashi, M.; Funazaki, H.; Mitsunaga, S.; Shimizu, S.; Ohno, I.; Takahashi, H.; Ichida, Y.; et al. Efficacy of prophylactic minocycline treatment for skin toxicities induced by erlotinib plus gemcitabine in patients with advanced pancreatic cancer: a retrospective study. *Am J Clin Dermatol* **2015**, *16*, 221-229, doi:10.1007/s40257-015-0116-x.
49. Shinohara, N.; Nonomura, N.; Eto, M.; Kimura, G.; Minami, H.; Tokunaga, S.; Naito, S. A randomized multicenter phase II trial on the efficacy of a hydrocolloid dressing containing ceramide with a low-friction external surface for hand-foot skin reaction caused by sorafenib in patients with renal cell carcinoma. *Annals of oncology : official journal of the european society for medical oncology* **2014**, *25*, 472-476, doi:10.1093/annonc/mdt541.
50. Shou, L.; Chen, J.; Shao, T.; Zhang, Y.; Zhao, S.; Chen, S.; Shu, Q. Clinical characteristics, treatment outcomes, and prognosis in patients with MKIs-associated hand-foot skin reaction: a retrospective study. *Support Care Cancer* **2023**, *31*, 375, doi:10.1007/s00520-023-07830-3.
51. Shou, L.; Shao, T.; Zhao, F.; Chen, S.; Chen, Q.; Shu, Q. The Efficacy and Safety of the Shouzu Ning Decoction Treatment for Multi-Kinase Inhibitors-Associated Severe Hand-Foot Skin Reaction. *Cancer Manag Res* **2021**, *13*, 45-53, doi:10.2147/cmar.S285002.
52. Tian, A.; Zhou, A.; Bi, X.; Hu, S.; Jiang, Z.; Zhang, W.; Huang, Z.; Shi, H.; Yang, B.; Chen, W. Efficacy of Topical Compound Danxiong Granules for Treatment of Dermatologic Toxicities Induced by Targeted Anticancer Therapy: A Randomized, Double-Blind, Placebo-Controlled Trial. *Evidence-based Complementary and Alternative Medicine* **2017**, *2017*, doi:10.1155/2017/3970601.
53. Tohyama, M.; Sakaguchi, C.; Nishina, T.; Hyodo, I. Possible involvement of zinc deficiency in epidermal growth factor receptor inhibitor-induced xerotic dermatitis. *J Dermatol* **2021**, *48*, 1579-1583, doi:10.1111/1346-8138.16049.
54. Vincenzi, B.; Trower, M.; Duggal, A.; Guglielmini, P.; Harris, P.; Jackson, D.; Lacouture, M.E.; Ratti, E.; Tonini, G.; Wood, A.; et al. Neurokinin-1 antagonist orvepitant for EGFRI-induced pruritus in patients with cancer: A randomised, placebo-controlled phase II trial. *BMJ Open* **2020**, *10*, doi:10.1136/bmjopen-2019-030114.
55. Wang, D.G.; Barrios, D.M.; Blinder, V.S.; Bromberg, J.F.; Drullinsky, P.R.; Funt, S.A.; Jhaveri, K.L.; Lake, D.E.; Lyons, T.; Modi, S.; et al. Dermatologic adverse events related to the PI3Kα inhibitor alpelisib (BYL719) in patients with breast cancer. *Breast Cancer Res Treat* **2020**, *183*, 227-237, doi:10.1007/s10549-020-05726-y.

56. Wang, Y.; Yang, Y.; Xu, J.; Yu, J.; Liu, X.; Gao, R.; Zhang, L. Randomized double-blind trial of prophylactic topical Evozac® calming skin spray for gefitinib-associated acne-like eruption. *OncoTargets and therapy* **2014**, *7*, 1261-1266, doi:10.2147/ott.s65961.
57. Wong, S.F.; Lindgren, A.; Mummaneni, M.; Byun, T.; Vasko, C.; Arenos, R.; Alexson, E.; Osann, K. A prospective crossover pilot study to evaluate the use of a topical wound gel in patients with cutaneous toxicity caused by epidermal growth factor receptor inhibitors. *Journal of Supportive Oncology* **2010**, *8*, 202-208, doi:10.1016/j.suponc.2010.09.003.
58. Wu, J.; Liu, D.; Offin, M.; Lezcano, C.; Torrisi, J.M.; Brownstein, S.; Hyman, D.M.; Gounder, M.M.; Abida, W.; Drilon, A.; et al. Characterization and management of ERK inhibitor associated dermatologic adverse events: analysis from a nonrandomized trial of ulixertinib for advanced cancers. *Invest New Drugs* **2021**, *39*, 785-795, doi:10.1007/s10637-020-01035-9.
59. Yamamoto, K.; Nishiyama, S.; Kunisada, M.; Iida, M.; Ito, T.; Ioroi, T.; Makimoto, H.; Omura, T.; Harada, K.; Fujisawa, M.; et al. Safety and Efficacy of Bis-Glyceryl Ascorbate as Prophylaxis for Hand-Foot Skin Reaction: A Single-Arm, Open-Label Phase I/II Study (DGA Study). *Oncologist* **2022**, *27*, e384-e392, doi:10.1093/oncolo/oyab067.
60. Yen, C.F.; Hsu, C.K.; Yang, H.S.; Lee, C.N.; Chi, C.C.; Chung, W.H.; Wang, C.L.; Pang, J.S.; Wang, C.W.; Ko, Y.S.; et al. Treatment of epidermal growth factor receptor inhibitor-induced severe paronychia with pyogenic granuloma-like lesions with topical betaxolol: an open-label observation study. *Int J Dermatol* **2020**, *59*, 326-332, doi:10.1111/ijd.14730.
61. Zhang, J.; Lu, X.; Zhang, X.; Tan, K.; Li, J.; Cui, H. Topical Zhiyang Pingfu Liquid for Moderate to Severe Skin Rash Associated With EGFRIs: a double-blinded randomized controlled trial. *Integrative cancer therapies* **2022**, *21*, 15347354221140409, doi:10.1177/15347354221140409.
